# Evolution and functional divergence of *NLRP *genes in mammalian reproductive systems

**DOI:** 10.1186/1471-2148-9-202

**Published:** 2009-08-14

**Authors:** Xin Tian, Géraldine Pascal, Philippe Monget

**Affiliations:** 1 Physiologie de la Reproduction et des Comportements, UMR 6175 INRA-CNRS-Université François Rabelais de Tours-Haras Nationaux, 37380 Nouzilly, France

## Abstract

**Background:**

NLRPs (Nucleotide-binding oligomerization domain, Leucine rich Repeat and Pyrin domain containing Proteins) are members of NLR (Nod-like receptors) protein family. Recent researches have shown that *NLRP *genes play important roles in both mammalian innate immune system and reproductive system. Several of *NLRP *genes were shown to be specifically expressed in the oocyte in mammals. The aim of the present work was to study how these genes evolved and diverged after their duplication, as well as whether natural selection played a role during their evolution.

**Results:**

By using *in silico *methods, we have evaluated the evolution and functional divergence of *NLRP *genes, in particular of mouse reproduction-related *Nlrp *genes. We found that (1) major *NLRP *genes have been duplicated before the divergence of mammals, with certain lineage-specific duplications in primates (*NLRP7 *and *11*) and in rodents (*Nlrp1*, *4 *and *9 *duplicates); (2) tandem duplication events gave rise to a mammalian reproduction-related *NLRP *cluster including *NLRP2*, *4*, *5*, *7*, *8*, *9*, *11*, *13 *and *14 *genes; (3) the function of mammalian oocyte-specific *NLRP *genes (*NLRP4*, *5*, *9 *and *14*) might have diverged during gene evolution; (4) recent segmental duplications concerning *Nlrp4 *copies and vomeronasal 1 receptor encoding genes (*V1r*) have been undertaken in the mouse; and (5) duplicates of *Nlrp4 *and *9 *in the mouse might have been subjected to adaptive evolution.

**Conclusion:**

In conclusion, this study brings us novel information on the evolution of mammalian reproduction-related *NLRPs*. On the one hand, *NLRP *genes duplicated and functionally diversified in mammalian reproductive systems (such as *NLRP4*, *5*, *9 *and *14*). On the other hand, during evolution, different lineages adapted to develop their own *NLRP *genes, particularly in reproductive function (such as the specific expansion of *Nlrp4 *and *Nlrp9 *in the mouse).

## Background

The innate immune system is an ancestral and ubiquitous system of defense against microbial infection and other potential threats to the host. The first mammalian molecules shown to be involved in innate immune recognition of, and defense against, microbial pathogens are the Toll-like receptors (TLRs), which constitute the main sensors for detection of extracellular microbes [1]. Recent research has shown that Nod-like receptors (NLRs) act as intracellular regulators of bacterial-induced inflammation [2-4].

The NLR protein family is separated into several subfamilies based on the different N-terminal effecter domains [5]. The NLRP subfamily (known also as NALP family) is a new identified NLR group characterized by a PYRIN domain. Initially, research on the function of NLRP proteins focused on their roles in apoptotic and inflammatory signaling pathways via the formation of a large signaling platform (named inflammasome) and the activation of caspases in innate immunity [4,6,7]. Interestingly, more and more researches have recently revealed that some *NLRP *genes play roles in mammalian reproduction. For instance, the mouse *Nlrp5 *(known also as *MATER*) was one of the first identified mammalian maternal effect genes, *i.e*. it encodes mRNA required for successful development of a fertilized oocyte [8]. In particular, the targeted invalidation of mouse oocyte-specific *Nlrp5 *leads to the sterility of females due to a blockage at the two-cell stage in the development of embryos [8]. *In vitro *knockdown experiments in mouse fertilized eggs also revealed that a decrease of mouse germ-cell-specific *Nlrp14 *transcripts results in an arrest of development between the one-cell and eight-cell stages of the embryos [9,10]. In the mouse, exclusive duplications of *Nlrp4 *and *Nlrp9 *have been detected with specific expression profiles restricted to the oocyte [9], whereas the mouse *Nlrp4a *and the cattle *NLRP9 *have also been reported to be expressed in the testis [11,12]. In humans, the mutations of *NLRP7 *are found to cause recurrent hydatidiform moles, spontaneous abortions, stillbirths and intrauterine growth retardation [13,14]. Additionally, the recent expression analyses of *NLRP *genes in the human and the rhesus macaque (*Macaca mulatta*) have shown that most if not all *NLRP *genes are expressed in primate gametes and early embryos, suggesting a role of *NLRPs *in primate pre-implantation development [15,16].

Overall, it seems that the *NLRP *genes play roles not only in innate immunity but also in the reproductive system of mammals. Moreover, *Nalp5 *and *Nalp14 *invalidation data in the mouse suggests that there is no functional compensation of each of these oocyte-specific genes. In this context, one question is to study how these reproduction-related *NLRP *genes evolved and diverged in function after their duplication, as well as whether natural selection played a role during their evolution.

The availability of several completely sequenced vertebrate genomes allows us to use a phylogenomic approach to identify the *NLRP *orthologues from different mammals and to evaluate the evolutionary features of *NLRPs *in different lineages. In this paper, we focus on the evolution and functional divergence of reproduction-related *NLRPs*, as well as lineage-specific expansion of *Nlrp4 *and *Nlrp9 *in rodents. The aim of the present work is to provide new information in understanding how *NLRP *genes evolve in reproductive system and how different mammals adapted to develop their own *NLRP *gene copies particularly in reproductive biology.

## Results

### Phylogeny and syntenic comparison of *NLRPs*

A total of 83 mammalian NLRP amino acid sequences with chicken NLRP3-like as outgroup were used for the reconstruction of phylogenetic trees. A total of 122 positions are included in the final dataset.

**Figure 1:**
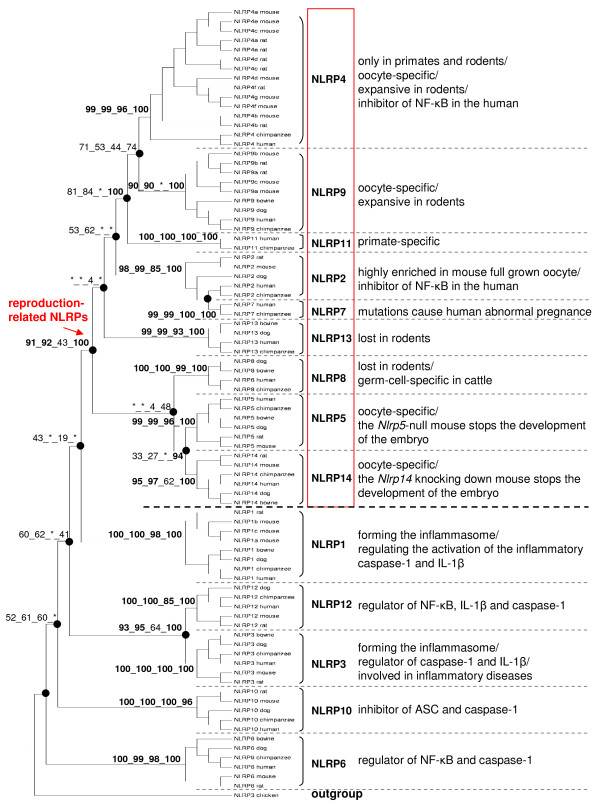
**Consensus phylogenetic tree of NLRPs reconstructed by the fusion of four separate methods (NJ, ME, MP and ML)**. A total of 83 NLRP protein sequences from 6 mammalian species (cattle, dog, human, chimpanzee, mouse and rat) are used with chicken as outgroup. A total of 122 amino acid sites are included in the analyses. The bootstrap values are labeled on the main branches for the four different methods (followed the order of NJ, ME, MP and ML methods). The symbol * means that the branch is not supported by the corresponding method.

In the consensus phylogenetic tree (Figure 1), each NLRP protein, except for NLRP2, is shown to be monophyletic by all four methods with high bootstrap values. NLRP2 encompasses the primate-specific NLRP7, suggesting the origin of *NLRP7 *from a duplication of the *NLRP2/7 *ancestor in primates. NLRP11 is also primate-specific and might be related to the reproduction-related NLRP4 and 9. NLRP4 proteins are only identified in primates and rodents, indicating the potential birth of the ancestral *NLRP4 *gene before the divergence between primates and rodents. Otherwise, the other *NLRP *genes seem to have duplicated before the divergence of mammals.

In rodents, three *Nlrp *genes show lineage-specific duplications including *Nlrp1*, *Nlrp4 *and *Nlrp9 *(Figure 1). More specifically, *Nlrp1 *exclusively duplicated in the mouse, *Nlrp9 *duplicated independently in the rodents after the separation of mouse and rat, whereas the duplication of *Nlrp4 *is likely to have occurred both before and after this separation.

Unexpectedly, *NLRP8 *and *NLRP13 *seem to be universal in mammals except for rodents. We speculate that these two genes might have been lost during evolution of the rodents. Unfortunately, our tBLASTn search (using the human *NLRP8 *and *13 *respectively as queries against the mouse genome) failed to find any trace of pseudogene of neither *NLRP8 *nor *NLRP13 *within the mouse conserved syntenic region. We found that, on the human chromosome 19, these two genes are located side by side between *NLRP4 *and *NLRP5*, whereas the mouse *Nlrp4 *gene has been expansively duplicated and the duplicates are located around *Nlrp5 *on the mouse chromosome 7. In rodents, the biological significance of the probable loss of *NLRP8 *and *13 *on one hand, and the parallel expansion of *Nlrp4 *on the other hand, remains unknown.

Remarkably, our phylogenetic analyses identified a well supported (bootstrap values >90% by ME, NJ and ML methods) reproduction-related clade including nine NLRP proteins: NLRP2, 4, 5, 7, 8, 9, 11, 13 and 14 (Figure 1). All these *NLRP *genes have been shown to be expressed in the human oocyte and pre-implantation embryos [16]. *NLRP4*, *5*, *8*, *9 *and *14 *are exclusively expressed in germ-cells, especially in the oocyte of mammals [8-12,17,18]. The mutations of *NLRP7 *are associated with abnormal embryo development in the human [13,14]. The expression of mouse *Nlrp2 *has been found to be highly enriched in fully grown oocytes but diminished in the 2-cell embryos upon embryo genome activation [19]. In both the human [16] and the rhesus macaque [15], *NLRP11 *and *13 *have been found specifically or preferentially expressed in the oocyte, with a similar expression pattern to other oocyte-specific *NLRP *genes, *i.e*., enriched in maturing oocytes and then progressively diminished in embryos, indicating an exclusive maternal origin of these transcripts. Unfortunately, the biological study of these two genes is not sufficient to address their putative roles in reproduction.

**Figure 2:**
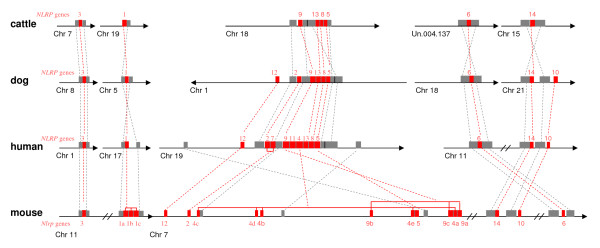
**Syntenic comparison of *NLRP *genes among the cattle, the dog, the human and the mouse**. The *NLRP *genes are marked in red, the orthologues are indicated by discontinuous lines. The order of *NLRPs *in each genome is listed as following: **The cattle: ***NLRP3 *(chr 7); *NLRP1 *(chr 19); *NLRP9, 13, 8 *and *5 *(chr 18); *NLRP6 *(Un.004.137); *NLRP14 *(chr 15). **The dog: ***NLRP3 *(chr 8); *NLRP1 *(chr 5); *NLRP12, 2, 9, 13, 8 *and *5 *(chr 1); *NLRP6 *(chr 18); *NLRP14 *and *10 *(chr 21). **The human: ***NLRP3 *(chr 1); *NLRP1 *(chr 17); *NLRP12, 2, 9, 13, 8 *and *5 *(chr 19); *NLRP6*, *NLRP14 *and *10 *(chr 11). **The mouse: ***Nlrp3, 1a, 1b *and *1c *(chr 11); *Nlrp12, 2, 4c, 4d, 4b, 9b, 4e, 5, 9c, 4a, 9a, 14, 10 *and *6 *(chr 7).

Moreover, this reproduction-related clade is also supported by the syntenic analysis (Figure 2). In the human genome, all the reproduction-related *NLRP *genes, except for *NLRP14*, are tandemly located on the chromosome 19q13.42, suggesting that multiple tandem duplication events might have given birth to these genes. The similar arrangements of the reproduction-related *NLRPs *are also found in cattle and dog genomes (Figure 2). In the mouse genome, the reproduction-related *Nlrps *(except for *Nlrp14*) are not located side by side, but interrupted by some other types of genes, especially and mainly by certain *V1r *genes (discussed below) on chromosome 7A1 and 7A3. Although *NLRP14 *is not located in the syntenic region encompassing other reproduction-related *NLRPs *in all the studied mammalian genomes (involving cattle, dog, human, chimpanzee, mouse and rat), our phylogenetic result and the published expression profile show that this gene is close to other reproduction-related *NLRPs*. This suggests that early (before the divergence of mammals) genomic rearrangements might have resulted in the separation of *NLRP14 *from its relatives.

We identified the other *NLRP *members (*NLRP1, 3, 6, 10 *and 12) as non-reproduction-related *NLRP *genes, because they are not in the reproduction-related clade in the phylogenetic trees and ubiquitously expressed. These genes have been shown to participate in inflammatory and immune responses by regulating the activation of other signaling factors, such as NF-κB, caspase-1 and IL-1β [20-24]. In contrast, little is known about the function of the reproduction-related NLRP proteins in inflammation and immunity, except that the human NLRP2, 4 and 7 may be able to inhibit IL-1β and/or NF-κB [25-28], and thus may contribute to modulate the inflammatory response. It is known that inflammation and bacterial infection can cause infertility, ectopic pregnancy and abortion [29]. Thus, NLRP proteins might play roles both in innate immunity and in reproductive biology.

### Functional divergence

In the reproduction-related *NLRP *cluster referred by the phylogenetic analysis above, there are several oocyte-specific *NLRP *genes including *NLRP4, 5, 8, 9 *and *14*. To investigate whether functional divergence have occurred following the duplications of these oocyte-specific *NLRPs*, four oocyte-expressed *NLRP *genes (*NLRP4*, *5*, *9 *and *14*) were estimated for functional divergence by using the DIVERGE program [30]. *NLRP8*, which was not found in rodents, was therefore not included in this analysis in order to compare the level of divergence across all candidate species.

After alignment and removal of the sites with gaps, a total of 650 amino acid sites are included in the analysis. Pairwise comparisons of each of the four oocyte-expressed *NLRP *gene clusters (Additional file 1) were carried out and the rate of amino acid evolution at each sequence position was estimated. As shown in Table 1, functional divergence is significant between each comparison (*θ *> 0 with *p *< 0.001) by both MFE and MLE methods, indicating that site-specific altered selective constraints should contribute to the functional evolution of the oocyte-specific *NLRP *genes after their duplication.

**Table 1:** Pairwise comparison of functional divergence of mouse oocyte-expressed *Nlrp *genes

Gene cluster	MFE *θ*	MLE *θ*	*p *value	RFD No.	Cutoff
*Nlrp4/9*	0.65 ± 0.07	0.61 ± 0.05	< 0.0001	91	0.78
*Nlrp4/14*	0.96 ± 0.08	0.89 ± 0.06	< 0.0001	118	0.94
*Nlrp4/5*	0.68 ± 0.08	0.67 ± 0.06	< 0.0001	61	0.84
*Nlrp9/14*	0.76 ± 0.08	0.65 ± 0.06	< 0.0001	59	0.80
*Nlrp5/9*	0.64 ± 0.09	0.58 ± 0.07	< 0.0001	53	0.75
*Nlrp5/14*	0.49 ± 0.10	0.45 ± 0.07	< 0.0001	33	0.70

*θ*, coefficient of functional divergence; *MFE*, Model-Free Method; *MLE*, Maximum-Likelihood Estimation under two-state model; *p *value, significance level calculated by the method of Fisher's transformation on z scale, and from chi square on LRT; *RFD No*., number of residues with predicted functional divergence;-*cutoff*, the minimal posterior probability for amino acids causing functional divergence, established empirically by progressively removing the highest scoring residues from the alignment until *θ *dropped to zero.

Furthermore, the important amino acid residues, responsible for functional divergence, were predicted by calculating the site-specific profile based on posterior analysis for all pairs of clusters with functional divergence. In order to extensively reduce false positive, cutoff values, *i.e*., the minimal posterior probabilities for RFD (Residues with predicted Functional Divergence) were established empirically by progressively removing the highest scoring residues from the alignment until *θ *dropped to zero. As shown in Table 1, the least RFD (33 residues, covering 5.1% of a total of 650 aligned sites) are observed between *NLRP5 *and *NLRP14*, and the most RFD (118 residues, covering 18.2% of a total of 650 aligned sites) are observed between *NLRP4 *and *NLRP14*. In general, the RFD are detected in all three functional domains (NACHT, PYRIN and LRRs) of NLRP proteins, implying that shifted functional constrains might have acted on each protein domain.

### Segmental duplications concerning *Nlrp4 *and *V1r *genes in the mouse genome

**Figure 3:**
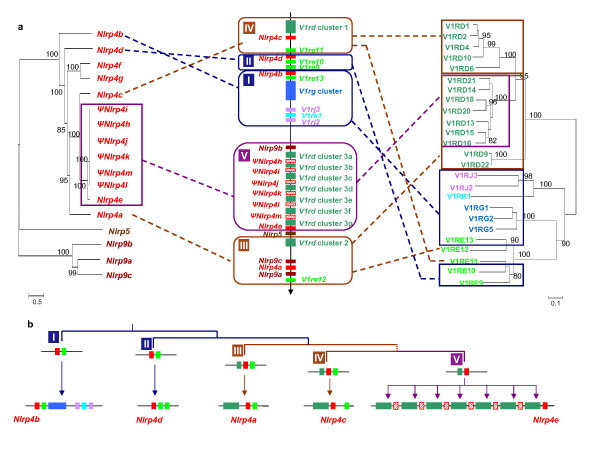
**Segmental duplications of *Nlrp4 *duplicates and *V1r *genes in the mouse genome**. Figure 3a Comparison of gene relationships in the phylogenetic tree and their genomic position. Left: Phylogenetic tree of mouse *Nlrp4 *duplicates (including pseudogenes) and its closely related *Nlrp5 *and *Nlrp9 *genes. A total of 439 nucleotide sites are included in the final dataset. Right: Phylogenetic tree of 25 mouse *V1r *proteins whose encoding genes are located in the same genomic region as *Nlrp4 *genes. A total of 271 amino acid sites are included in the final dataset. The statistical confidence of each branch was estimated by the bootstrap method with 1000 replications, and only the values ≥ 80% are labeled in the trees. Middle: Genomic organization of related *Nlrp *and *V1r *genes. Five putative regions (I - V) might be concerned in segmental duplication. Figure 3b A possible scenario of segmental duplication deduced from synteny combining phylogeny: Genomic region I (involving *Nlrp4b*) and region II (involving *Nlrp4d*) might have been resulted from an early duplication, and the subsequence duplication events have given birth to region III (involving *Nlrp4a*) and region IV (involving *Nlrp4c*). Region V might have been duplicated later, and in this genomic region, other more recent segmental duplications (concerning pseudogenized *Nlrp4 *copy and *V1rd *genes) have been undertaken several times. We note that the order of duplication events in this potential scenario is mainly based on the topology of *Nlrp4 *phylogenetic tree. There might be other alternative itinerary to explain the present complex pattern of this genomic region.

In the mouse, there are seven *Nlrp4 *gene copies (named from *Nlrp4a *to *4g*) which are specifically expressed in oocytes [9,11]. Our lab has also found the expression of *Nlrp4a *in the testis by RT-PCR [11]. *Nlrp4a-4e *genes are located on chromosome 7A, but *Nlrp4f *and *4g *are located on chromosomes 13B3 and chromosome 9 respectively. By the tBLASTn method, we identified six additional putative pseudogenes on chromosome 7A (named here *ΨNlrp4h-4m*, Figure 3a). Based on the phylogenetic tree (Figure 3a), multiple duplications of mouse *Nlrp4 *could be depicted as follows. *Nlrp4b *might be the earliest *Nlrp4 *copy in the mouse genome with an orthologue present in the rat genome, suggesting an origin before the separation of mouse and rat. The duplication of ancestral *Nlrp4b *is supposed to have given rise to *Nlrp4d*, and the subsequent duplications might have resulted in *Nlrp4f/4g *and *Nlrp4a/4c*. Subsequent chromosomal rearrangements might have caused the independent locations of *Nlrp4f *and *Nlrp4g *with other *Nlrp4 *duplicates. All the putative *Nlrp4h-4m *pseudogene sequences show high identities with each other as well as with *Nlrp4e*. In fact, in all the pseudogenes, a common frameshift of 1-bp deletion was detected at the position corresponding to 358aa of *Nlrp4e *and it caused a subsequent prematural stop codon at the position of 434aa, suggesting that the pseudogenization had happened before duplications of these copies.

In the mouse genome, *Nlrp4 *duplicates (except for *Nlrp4f *and *4g*) are located between 6.00 Mb and 27.32 Mb on the chromosome 7. Interestingly, all these *Nlrp4 *duplicates are found next to the vomeronasal 1 receptor encoding genes (*V1r*), such as *V1rd *and *V1re *genes (Figure 3a). The phylogeny and genomic location of these *V1r *genes reveal that *V1r *genes might have a similar duplication model to their neighbors, *Nlrp4 *genes (Figure 3a). Thus, one may hypothesize that *Nlrp4 *may have not duplicated one by one, but were rather involved in segmental duplications encompassing several different genes, such as *V1r *genes.

Vomeronasal organ, which is well developed in rodents in comparison with human, detects pheromones and other chemical signals implicated in innate reproductive and social behaviours between the members of the same species [31,32]. The *V1r *gene family is supposed to have emerged during the divergence of placental mammals (80-110 MYA), but many subfamilies including *V1rd *and *V1re *originated only in rodents after their separation from the primates [33], which is consistent with the rodent-specific duplication of *Nlrp4*. Thus, the phylogenetic relationship and genomic location (Figure 3a) of these *V1r *genes, that are located in the segmental duplication region, could help us to better understand the segmental duplications. Overall, as shown in Figure 3b, a presumed itinerary of segmental duplication was deduced by combining phylogeny with genomic location of both *Nlrp4 *duplicates and *V1r *genes. Although we can not confirm the order of duplication events by the present data, we can state that segmental duplication events characterize the evolution of this genomic region.

### Positive selection on *Nlrp4 *and *Nlrp9 *paralogues in the mouse

Several examples have shown that genes in duplication blocks could be maintained by positive selection [34-37]. Here, we implemented PAML4 [38] to investigate the model of selective force acting on mouse *Nlrp4 *and *Nlrp9 *duplicates during their evolution.

By using Site Models, estimates of the parameter values under M2a and M8 (Table 2) indicate that a fraction of sites (about 5-6%) may be under positive selection in both datasets (*Nlrp4*_mouse and *Nlrp9*_mouse). LRTs of all comparisons (including M1a versus M2a, M7 versus M8 and M8a versus M8) are significant (*p *< 0.001) for both datasets (Table 2), implying that selective forces varied among amino acid sites and that the mouse *Nlrp4 *and *9 *genes might have been subjected to positive selection after their expansion. Furthermore, the comparison of model MEC (AICc = 15913.11 for *Nlrp4*_mouse and AICc = 14196.22 for *Nlrp9*_mouse) versus model M8a (AICc = 16003.68 for *Nlrp4*_mouse and AICc = 14293.43 for *Nlrp9*_mouse) also supports positive selection in these two gene clusters. The AICc scores of MEC are lower than those of M8a, indicating that MEC better fits the data, *i.e*. positive selection is significant.

**Table 2:** Parameter estimates and likelihood scores for site models in PAML

model	*l*	Estimates of parameters	2Δ*l*	Positively selected sites (BEB)
*Nlrp4*_mouse				

M1a	-7999.63	ρ_0 _= 0.33		Not allowed
M2a	-7986.58	ρ_0 _= 0.28,(ρ_1 _= 0.67), ρ_s _= 0.05 ***ω*_s _= 3.93**	26.09* (M2a vs M1a)	2 sites > 95% 133C, 677V
M7	-7999.85	*p *= 0.04, *q *= 0.02		Not allowed
M8a	-7999.55	ρ_0 _= 0.34,(ρ_1_= 0.66), *p *= 0.81, *q *= 6.69	27.56* (M8 vs M7)	Not allowed
M8	-7986.07	ρ_0 _= 0.94,ρ_s _= 0.06, *p *= 0.20, *q *= 0.09, ***ω*_s _= 3.64**	26.98* (M8 vs M8a)	4 sites > 95% 133C, 153S, 657N, 677V

*Nlrp9*_mouse				

M1a	-7140.29	ρ_0 _= 0.36043		Not allowed
M2a	-7107.99	ρ_0 _= 0.28, ρ_1 _= 0.66, ρ_s _= 0.06, ***ω*_2 _= 10.17**	64.60* (M2a vs M1a)	10 sites > 95%, 2 sites > 99% 106H, 123 E, 356F, 360L, **378R**, 513E, **536V**, 571V, 661L, 948A
M7	-7140.98	*p *= 0.02, *q *= 0.01		Not allowed
M8a	-7140.29	ρ_0 _= 0.36,(ρ_1 _= 0.64), *p *= 0.01, *q *= 98.97	65.99* (M8 vs M7)	Not allowed
M8	-7107.99	ρ_0 _= 0.94, ρ_s _= 0.06, *p *= 0.01, *q *= 0.01, ***ω*_s _= 10.20**	64.60* (M8 vs M8a)	11 sites > 95%, 6 sites > 99% 106H, **123 E**, 278L, 356F, **360L**, **378R**, 513E, **536V**, **571V**, 661L, **948A**

NOTE. - Log-likelihood values (*l) *are given for each model. The value *ω*_S _is the average d_N_/d_S _ratio for sites under positive selection in the models M2a and M8, p and q are the shape parameters for the beta distribution of *ω *in M7 and M8. ρ_0_, ρ_1_, and ρ_S _are the proportions of codons subject to purifying, neutral, and positive selection, respectively. * *p *value is significant at 0.001. Predicted positively selected sites with posterior probabilities > 95% are listed, and the sites with *p *> 99% are in boldface. Site numbers and amino acids refer to the reference sequences *Nlrp4a *(NP_766484) and *Nlrp9a *(NP_001041684) for the two datasets, respectively.

Subsequently, we wanted to know if the target of positive selection had changed during evolution in these two gene clades. We compared the potential sites subjected to positive selection between the two datasets (Table 2). In *Nlrp4*_mouse dataset, 2 sites (133C and 677V) are identified as positively selected sites at *p *> 95% level by both M2a and M8 models, and 2 more sites (153S and 657N) are identified only by the M8 model. In *Nlrp9*_mouse dataset, 10 sites (106H, 123 E, 356F, 360L, 378R, 513E, 536V, 571V, 661L and 948A) are identified as potential targets of positive selection by both M2a and M8 models, and 1 more sites (278L) is identified only by the M8 model. We note that no common sites are shared by the two datasets as positively selected sites at *p *> 95% level, suggesting a dramatic shift in the target of positive selection between the *Nlrp4 *and *Nlrp9 *genes in the mouse. When the 3D structures or models of these proteins are available in the future, we could investigate whether these positively selected sites are located in functionally pivotal regions.

## Discussion

### Independent evolution of reproduction-related *NLRP *genes in different mammalian lineages

Phylogenetic analyses show that *NLRP *genes have been originated and duplicated before the divergence of mammals. Certain genes are well conserved during their evolution, such as *NLRP3 *(82% identity between the human and the mouse), whereas many genes involved in reproduction have rapidly evolved resulting in higher sequence divergence among lineages, such as *NLRP5 *(50% identity between the human and the mouse) and *NLRP14 *(62% identity between the human and the mouse). The pairwise estimates of evolutionary divergence between the reproduction-related paralogues are ranged from 61% to 75% (except that the pairwise distance between *NLRP2 *and its recent primate-specific duplicate, *NLRP7*, is 45%), which is obviously higher than the one between the non-reproduction-related paralogues (ranged from 43% to 69%). Moreover, gene duplication and/or gene loss is found to have occurred independently in different mammalian lineages. On the one hand, *Nlrp4 *(originated before the divergence of primates and rodents) and *Nlrp9 *have been extensively duplicated in rodents, and other lineage-specific gene duplication events concern the specific duplication of *Nlrp1 *(three paralogues) in the mouse as well as the unique origin of *NLRP7 *and *NLRP11 *in primates. On the other hand, *Nlrp8 *and *Nlrp13 *are seemed to be lost from the genomes of rodents. Interestingly, the major gene duplication and gene loss events are found to be associated with the reproduction-related genes (such as *NLRP4*, *7*, *8*, *9*, *11 *and *13*), implying that reproduction-related genes have undertaken a fast and diverged evolution among different mammalian lineages. Given note that major reproduction-related *NLRP *genes are germ-cell specific, whereas the other non-reproduction-related genes are usually expressed in multiple tissues from a single organism. Recent large-scale gene expression studies have shown that the tissue specificity of genes is correlated positively with gene evolution rates [39-41]. Furthermore, our result reinforces the hypothesis that reproduction-related genes are most highly divergent and evolve more rapidly than genes expressed in other organs [42-45].

### Functional divergence of the oocyte-specific genes

Gene duplication is thought to be the essential source of gene novelty, with new or altered functions, as shown by widespread existence of gene families. Among *NLRP *genes, there are at least four oocyte specifically expressed in mammals, including *NLRP4*, *5*, *9 *and *14*. These genes have been found to be restricted expressed in oocytes and early embryos [9,15], suggesting their important roles in oogenesis and preimplantation embryo development. Additionally, their temporal expression in oocytes and degradation during preimplantation development coincide with the timing of gene expression transition from maternal to zygotic control, indicating their roles as maternal effect genes.

In the present work, by using an *in silico *method (with the help of the program DIVERGE), we demonstrate that the functional divergence could have occurred between each pair of oocyte-specific *NLRP *genes. This result is supported by certain published experimental data. For example, the knock-out of mouse *Nlrp5 *induced female infertility due to a blockage of early embryonic development [8]; and the injection of siRNA against *Nlrp14 *into fertilized eggs resulted in arrested development of embryos between 1-cell and 8-cell stages [9]. These results suggest that the expression of other oocyte-specific *NLRP *genes is not able to compensate the absence of neither *NLRP5 *nor *NLRP14 *genes. Additionally, recent research has shown that *NLRP14 *is also expressed in the testis and its mutation might cause spermatogenic failure in the human [46]. Thus, we presume that the expressional divergence of *NLRP5 *and *NLRP14 *during the development of mouse germ-cells and embryos may lead to functional specialization. On the other hand, target invalidation/inhibition of *NLRP5 *and *14 *did not reveal any deleterious effect on ovarian folliculogenesis but rather in early embryonic development, suggesting other genes might play the similar roles during folliculogenesis and meiotic maturation. For these latter functions, plausible candidates could be other oocyte-specific *NLRP *genes, such as *NLRP4 *and *NLRP9*. To investigate this possibility, further functional studies such as knock-out models or other targeted inhibition experiments on these genes should be carried out.

Gene duplication is thought to be a major driving force in enabling the evolution of tissue specialization. By using microarray gene expression data from mammals [47], fruit flies and yeast [48], it has recently shown that as gene family size enlarges, there is a general trend for paralogous genes with decreased breadth and increased specificity of expression. In particular, by studying the relationship between gene family size and expression breadth of 1249 protein families in the mouse, Freilich and his colleagues [49] have recently demonstrated that duplicates that arose through post-multicellularity duplication events show a tendency to become more specifically expressed, supporting the view suggested by the subfunctionalization model [50]. In our case, especially with the evidence that *NLRP5 *and *NLRP14 *genes are significantly involved in different developmental stages of embryos, we hypothesize that the functional divergence of oocyte-specific *NLRP *duplicates might be derived by the expression specialization of duplicate genes, which could provide the mammals reproductive advantages such as in adaptation.

### Segmental duplication in the mouse genome

The phylogenetic research indicates the rodent-specific expansion of *Nlrp4 *and *NLRP9*, and the furthermore genomic analyses find that these *Nlrp *duplicates, especially *Nlrp4*, have been duplicated together with other genes, such as *V1r *copies. This segmental duplication is not rare during the mouse and human genomic evolution. It has recently revealed that the segmental duplication constitutes about 5% of mouse genome, and its distribution is in a highly non-random fashion [51]. Interestingly, the duplication blocks account for 32% of the first 50 Mb of the chromosome 7, where the segmental duplications concerning *Nlrp4 *and *V1r *genes are located. *Nlrp9 *has been also duplicated in this region, but its duplication scenario is not clear and might be due to subsequent recombination.

As found in the human [52], the segmental duplication regions in the mouse might also be "hot spots" for the occurrence of non-allelic homologous recombination, leading to genomic mutations such as deletion, duplication, inversion or translocation [53-55]. Thus, these instable regions might have a biological significance in form of genome evolution [56,57].

### Adaptive diversification after gene duplications in *Nlrp4 *and *Nlrp9*

In the mouse, *Nlrp4 *and *Nlrp9 *are specifically extensive in gene copies. By using Site-Models implemented in PAML4, we evaluated the variation of selective pressure acting on *Nlrp4 *and *Nlrp9 *duplicates, respectively. Our result shows that positive selection is significant in both datasets, which consistent with other reports referring that the rapid divergence of the reproductive genes may be driven by positive selection [43,58]. In particular, in the mouse, several gene cluster concerning lineage-specific expansion of reproduction-related genes have been found to be under adaptive evolution (positive selection), such as the *Sva *(seminal vesicle autoantigen) gene cluster [59], the *Prame *(or oogenesin) gene cluster [60], the *Psg *(pregnancy-specific glycoprotein) gene cluster [61], the *Rhox4 *gene cluster [62] and the *Svs *(seminal vesicle secretion proteins) gene cluster [63]. The lineage-independent expansion and subsequently rapid evolution of such genes might contribute to speciation or adaptation, such as genetic barriers between species and hybrid incompatibilities, or provide the species specification in reproductive processes such as sperm competition, host immunity to pathogens, and manipulation of female/male reproductive physiology and behavior. In the case of *NLRP4 *and *9 *genes, their biological functions are still unclear, so we can not evaluate the significance of their specific duplications in the reproduction of the mouse. However, the similar evolutionary mechanism driven by positive selection suggests that after duplication, these *NLRP *gene copies diverged and acquired abilities (probably by subfunctionalization) to adapt the new environment. In the subsequent research, the adaptive evolution of the other reproduction-related *NLRP *genes could be further evaluated. Hopefully, when the biological functions of these *NLRP *genes are elucidated, the nature of the selective pressure acting on *NLRPs *will be better understood.

Interestingly, the evolution of *V1r *genes, the other genes involved in the segmental duplication with *Nlrp4 *and *9*, is also driven by positive selection in rodents [33]. V1R proteins are thought to be responsible for the detection of pheromones that induce innate reproductive behaviors between members of the same species [31,32]. Thus, it is presumed that the adaptive evolution of the *V1r *genes might play an important role in reinforcing pre-zygotic barriers among species of rodents [33].

## Conclusion

*NLRP *genes have originated and duplicated mainly before the divergence of mammals. During evolution, *NLRPs*, especially the reproduction-related *NLRP *genes, have undergone a fast and independent diversification in different mammalian lineages. The expansion of reproduction-related *NLRP *genes has been associated with functional divergence after duplication, suggesting that each *NLRP *oocyte-specific gene might play an essential role in oogenesis and early preimplantation embryo development. The mouse-specific expansion of *Nlrp4 *and *Nlrp9*, concerning segmental duplication events, has been driven by positive evolution. Our founding suggests that the duplication and functional divergence of *NLRP *might provide mammals advantages in reproductive biology.

## Methods

### Molecular phylogenetic and syntenic analyses

The protein sequences of all the 14 known human NLRPs were retrieved from the GenBank http://www.ncbi.nlm.nih.gov/. The NLRP proteins from other species were searched by PSI-BLAST [64] with human NLRP protein sequences as queries against the protein databases (NCBI: RefSeq protein databases) of chimpanzee (*Pan troglodytes*), dog (*Canis familiaris*), cattle (*Bos taurus*), mouse (*Mus musculus*), rat (*Rattus norvegicus*) and chicken (*Gallus gallus*). The predicted coding sequences of the best hit proteins were retrieved when the hits presented more than 80% in length to be aligned with the query sequence (with E values < 10^-100^). These settings could effectively detect the potential NLRP members from different species but avoid involving the relative NLR proteins from other protein subfamilies. After removal of redundant sequences, the initial data set (Additional file 2) for NLRP phylogenetic studies includes 83 protein sequences from 6 mammals and 1 sequence from chicken.

Analyses of the orthologous and paralogous relationships among different species were carried out by combining the phylogenetic reconstruction of the gene family with the syntenic comparison. Multiple sequence alignments were performed using the Clustal W algorithm [65] and then manually edited. The alignment was submitted as Online Additional file 3. All alignment gap sites were eliminated before phylogenetic analyses. The phylogenetic trees were reconstructed with Neighbor Joining (NJ), Minimum-Evolution (ME) and Maximum Parsimony (MP) methods implemented in MEGA4 [66], as well as with Maximum Likelihood (ML) method implemented in PhyML [67]. The consensus phylogenetic tree [68] was generated by the fusion of the independent trees reconstructed by the four methods. In all analyses, chicken NLRP protein (Q5F3J4) was treated as the root of all the mammalian NLRPs. The bootstrap values [69] were estimated with 1000 replications. The syntenic comparison is based on Ensembl http://www.ensembl.org/index.html utilities such as "orthologue prediction" and "view syntenic regions".

### Test for functional divergence of oocyte-specific *NLRP *genes

The program DIVERGE [30] was used to estimate type I functional divergence [70,71] between oocyte-expressed *NLRP *paralogues (*NLRP4*, *5*, *9 *and *14*). Type I sites represent amino acid residues conserved in one clade (gene cluster) but highly variable in another, suggesting that these residues have been subjected to different functional constraints. Statistically, this functional divergence between two clades is measured by the coefficient of functional divergence, *θ*, ranging from 0 to 1. A null hypothesis of *θ *= 0 indicates that the evolutionary rate is virtually the same between two duplicate genes at each site [70,71]. If the null hypothesis was rejected, a site-specific profile was then used to predict the critical amino acid residues most likely to be responsible for the detected functional divergence.

A set of 33 protein sequences from mammalian NLRP4, 5, 9 and 14 is included in the analysis. Sequences of rat NLRP4d, 9a and 14 were excluded from the alignment because of their shorter lengths. Before the use of DIVERGE, our sequence dataset was examined to satisfy the conditions recommended by Gu et Vander Velden (2002), which permit us to obtain higher efficiency of detecting functional divergence-related residues: 1) each cluster has more than four amino acid sequences; (2) all pairwise sequence identities are <90%; and (3) multiple alignment is reliable. The phylogenetic tree used for DIVERGE was reconstructed by MEGA4 [66] with ME method. The coefficients of functional divergence (*θ*) between gene clusters are calculated by Model-Free Estimation (MFE) and Maximum-Likelihood Estimation under two-state model (MLE). To detect amino acid residues reflecting functional divergence, all four reproduction-related *NLRP *gene clades were compared to each other.

### Investigation of mouse specific segmental duplications

In order to investigate the predicted segmental duplications concerning *Nlrp4 *duplicates on the mouse chromosome 7, a tBLASTn search [64] was implemented by using the mouse *Nlrp4a *(the longest *Nlrp4 *sequence) as a query against the mouse genome. This method permitted us to identify both expressed *Nlrp4 *genes and traces of the pseudogenes. Then we combined phylogenetic analyses and genomic mapping to identify the segmental duplication [35]. The phylogenetic tree of mouse *Nlrp4 *genes and pseudogenes was reconstructed by using nucleotide sequences instead of protein sequences. The phylogenetic tree was also reconstructed for vomeronasal 1 receptor proteins (V1R), because their encoding genes are located paralleling to *Nlrp4 *duplicates in the mouse genome and they are presumed to have duplicated together with *Nlrp4*. The accession numbers of the V1R protein sequences used here are listed in Additional file 4. The genomic organization of all the concerned genes and pseudogenes in the mouse is inferred from Ensembl (release 49).

### Evolutionary analyses

To examine whether the duplicates of *Nlrp4 *and *Nlrp9 *in the mouse have been submitted to adaptive evolution, an analysis of variation in selective pressure following gene duplication events was carried out with the CODEML program implemented in PAML4 [38]. The alignments were resulted from Clustal W and PAL2NAL [72]. The shorter mouse *Nlrp4g *was excluded in order to obtain more informative sites.

Two datasets named *Nlrp4*_mouse (including 7 sequences) and *Nlrp9*_mouse (including 3 sequences) were investigated for different Site Models [73] implemented in PAML4. In this study, we employed three pairs of models including M1a (NearlyNeutral: 0< ω_0 _<1 and ω_1 _= 1) versus M2a (PositiveSelection: 0< ω_0 _<1, ω_1 _= 1 and ω_s _>1) [73], M7 (beta: 0< ω <1) versus M8 (beta and *ω*: 0< ω <1 and ω_s _>1) [74], and M8a (beta &*ω*_s _= 1: 0< ω <1 and ω_s _= 1) versus M8 [75]. LRTs (Likelihood Ratio Tests) were used to test for significant differences in the fit of the models incorporating selection relative to their (nested) counterparts that did not allow positive selection [73]. These tests provide a useful series of metrices for interpreting the significance of the results and a degree of protection against false positives [76]. Bayes empirical Bayes (BEB) method [77] implemented in PAML4 was used to estimate posterior probabilities of selection on each codon. Furthermore, a newly described model MEC [78] was also employed on the Selecton Server [79] to compare with the results from other models for positive selection. The advantage of the MEC model over the other models used here is that by treating specifically each amino-acid replacement, *Ka *is computed differently. So under the MEC model, a position with radical replacements will obtain a higher *Ka *value than a position with more moderate replacements [78].

## Authors' contributions

XT performed the main data collection and analyses. GP helped to guide in the bioinformatic analyses. PM conceived the study and helped to guide in the general analyses. All coauthors participated in manuscript preparation and review.

## Supplementary Material

Additional file 1 **The phylogenetic tree of oocyte-expressed *NLRPs *for DIVERGE analyses**. The figure shows the phylogenetic tree of oocyte-specific *NLRPs *derived from amino acid sequences for DIVERGE analyses. Click here for file

Additional file 2 **The *NLRP *sampling for all the analyses**. The table shows the *NLRP *sampling (including the gene symbol, their genomic location and the accession number) for all the analyses. Click here for file

Additional file 3 **The Clustal W alignment of 84 NLRP protein sequences for phylogenetic analyses**. The data shows the alignment result of 84 NLRP protein sequences used for phylogenetic analyses (Figure 1). Click here for file

Additional file 4 **The mouse *V1r *sampling used for phylogeny**. The Table shows the mouse *V1r *sampling used for phylogenetic analysis (Figure 3). Click here for file
