# Supplementary material for: Evolution and functional divergence of NLRP genes in mammalian reproductive systems
Source: BMC Evol Biol. 2009 Aug 14;9:202. doi: 10.1186/1471-2148-9-202 (PMC2735741; doi:10.1186/1471-2148-9-202)
Supplement: Additional file 1 — The phylogenetic tree of oocyte-expressed NLRPs for DIVERGE analyses. The figure shows the phylogenetic tree of oocyte-specific NLRPs derived from amino acid sequences for DIVERGE analyses. [file 1471-2148-9-202-S1.pdf]

Additional file 1: The phylogenetic tree of oocyte-specific *NLRPs* for DIVERGE analyses

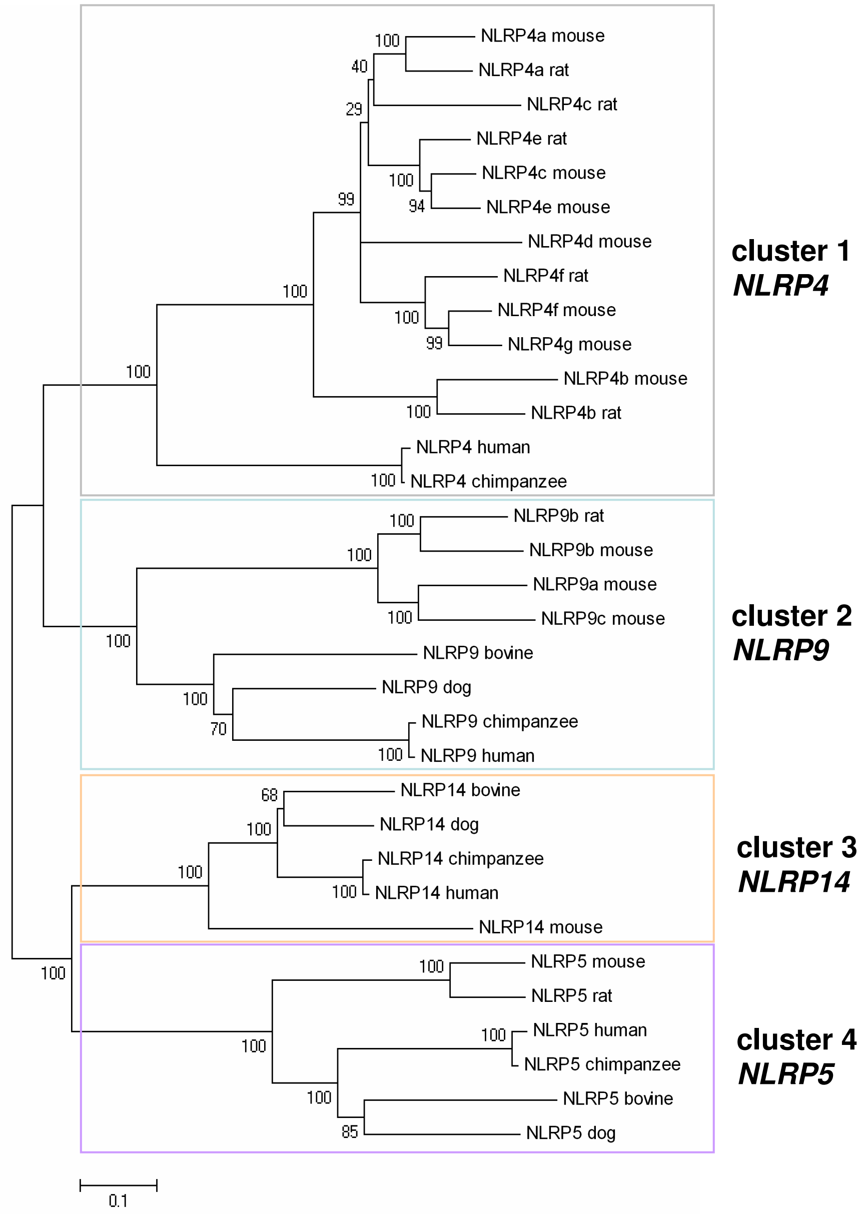

Figure S1 Phylogenetic tree of oocyte-specific *NLRPs* derived from amino acid sequences for DIVERGE analyses: Four oocyte-specific *NLRP* genes (*NLRP4*, *NLRP9*, *NLRP14* and *NLRP5*) are grouped into four clusters (cluster 1-4). Each gene (cluster) forms a monophyly with 100% boostrap value.
