# Supplementary material for: Evolution and functional divergence of NLRP genes in mammalian reproductive systems
Source: BMC Evol Biol. 2009 Aug 14;9:202. doi: 10.1186/1471-2148-9-202 (PMC2735741; doi:10.1186/1471-2148-9-202)
Supplement: Additional file 2 — The NLRP sampling for all the analyses. The table shows the NLRP sampling (including the gene symbol, their genomic location and the accession number) for all the analyses. [file 1471-2148-9-202-S2.pdf]

Additional file 2: the *NLRP* sampling for all the analyses

Species	Gene Symbol	Genomic Location	Protein Accession Number (*Gene Accession Number)*
human (*Homo sapiens*)	*NLRP1*	17p13.2	NP_127497
	NLRP2	19q13.42	NP_060322
	NLRP3	1q44	NP_004886
	NLRP4	19q13.42	NP_604393
	NLRP5	19q13.42	NP_703148
	NLRP6	11p15	NP_612202
	NLRP7	19q13.42	NP_996611
	NLRP8	19q13.42	NP_789781
	NLRP9	19q13.42	NP_789790
	NLRP10	11p15.4	NP_789791
	NLRP11	19q13.42	NP_659444
	NLRP12	19q13.41	NP_653288
	NLRP13	19q13.42	NP_789780
	NLRP14	11q23.3	NP_789792
chimpanzee (*Pan troglodytes*)	*NLRP1*	17	XP_001167230
	NLRP2	19	XP_001175071
	NLRP3	1	ENSPTRP00000003727
	NLRP4	19	XP_524404
	NLRP5	19	XP_001139299
	NLRP6	11	XP_521701
	NLRP7	19	XP_512902
	NLRP8	19	XP_512922
	NLRP9	19	ENSPTRP00000019820
	NLRP10	11	ENSPTRP00000005809
	NLRP11	19	XP_524402
	NLRP12	19	XP_524387
	NLRP13	19	XP_524405
	NLRP14	11	XP_521822
mouse (*Mus musculus*)	*Nlrp1a*	11B4	NP_001004142
	Nlrp1b	11B4	NP_001035786
	Nlrp1c	11B4	NP_001034323
	Nlrp2	7A1	NP_808358
	Nlrp3	11B1.3	NP_665826
	Nlrp4a	7A3	NP_766484 (*NM_172896)
	Nlrp4b	7A1	NP_766069 (*NM_172481)
	Nlrp4c	7A1	NP_113566 (*NM_031389)
	Nlrp4d	7A1	NP_001481360 (*XM_001481310)
	Nlrp4e	7A3	NP_001004194 (*NM_001004194)
	Nlrp4f	13B3	NP_780499 (*NM_175290)
	Nlrp4g	9	NP_001004145 (*NM_001004145)
	ΨNlrp4h	7A3	(*XR_035705)
	ΨNlrp4i	7A3	(*XR_034113)
	ΨNlrp4j	7A3	(*XR_034766)
	ΨNlrp4k	7A3	(*XR_035707)
	ΨNlrp4l	7A3	(*XR_035708)
	ΨNlrp4m	7A3	(*XR_034805)
	Nlrp5	7A3	NP_035990 (*NM_011860)

species	*Gene Symbol*	Genomic Location	Protein Sequence
mouse (*Mus musculus*)	*Nlrp6*	7F5	NP_001074858
	Nlrp9a	7A3	NP_001041684 (*NM_001048219)
	Nlrp9b	7A3	NP_918947 (*NM_194058)
	Nlrp9c	7A3	NP_001036077 (*NM_110142612)
	Nlrp10	7E3	NP_780741
	Nlrp12	7A1	Q08EE9
	Nlrp14	7E3	NP_001002894
Rat (*Rattus norvegicus*)	*Nlrp1*	10q24	XP_340836
	Nlrp2	1	XP_001070923
	Nlrp3	10q22	XP_220513
	Nlrp4a	1q12	NP_001099690
	Nlrp4b	1q21	XP_344857
	Nlrp4c	7q11	XP_234957
	Nlrp4d	11q12	ENSRNOP00000051463
	Nlrp4e	1q12	XP_574350
	Nlrp4f	1q12	XP_001076792
	Nlrp5	1q12	NP_001100944
	Nlrp6	1q41	XP_577848
	Nlrp9a	1q12	XP_218250
	Nlrp9b	1q12	XP_218248
	Nlrp10	1q33	NP_001099761
	Nlrp12	1q12	XP_218181
	Nlrp14	1q33	ENSRNOP00000040262
bovine (*Bos taurus*)	*NLRP1*	19	ENSBTAP00000027232
	NLRP3	7	XP_581687
	NLRP5	18	NP_001007815
	NLRP6	Un.004.137	XP_581339
	NLRP8	18	XP_582571
	NLRP9	18	NP_001019835
	NLRP13	18	XP_608304
	NLRP14	15	ENSBTAP00000002954
dog (*Canis familiaris*)	*NLRP1*	5	ENSCAFP00000022816
	NLRP2	1	ENSCAFP00000003831
	NLRP3	8	XP_848377
	NLRP5	1	XP_533576
	NLRP6	18	ENSCAFP0000009685
	NLRP8	1	XP_853947
	NLRP9	1	XP_533578
	NLRP10	21	XP_854074
	NLRP12	1	ENSCAFP00000003989
	NLRP13	1	XP_541402
	NLRP14	21	XP_542482
chicken (*Gallus gallus*)	*NLRP3*	5	Q5F3J4

UN: genomic location is unknown. * gene sequences used in the analysis of mouse segmental duplication (Fig. 2a).
