# Supplementary material for: Evolution and functional divergence of NLRP genes in mammalian reproductive systems
Source: BMC Evol Biol. 2009 Aug 14;9:202. doi: 10.1186/1471-2148-9-202 (PMC2735741; doi:10.1186/1471-2148-9-202)
Supplement: Additional file 3 — The Clustal W alignment of 84 NLRP protein sequences for phylogenetic analyses. The data shows the alignment result of 84 NLRP protein sequences used for phylogenetic analyses (Figure 1). [file 1471-2148-9-202-S3.pdf]

Additional file 3: Clustal W alignment of 84 NLRP protein sequences for phylogenetic analyses

```
#mega
!Title ;
!Format DataType=Protein indel=-;

#NLRP4a_mouse "gi|27370372|ref|NP_766484.1| NLR family, pyrin domain containing 4A [Mus musculus]"
------------------------------------------------------------
------------------------------------------------------------
------------------------MASFFSDFGLMWYLEELNKKEFVKFKEFLKQEVLQL
GLKQVS-------WTEVKKASRQDLASLLLKHYEEKPAWDMTFRFFQKINRKDLIERAKR
EIDG--------------------------------------------------------
-------------------------------------------------CPKLYRAHMKT
KMTHDSSRAFTISIQNFLKEKFTEDDYDCFENLFQSKGTESKPQV--VFLSGGAGVGKTL
MLKRLMLAWIESPVFLHKFSYIFYFCCREVK-QLKTASLAELISREWPGPSAPIEEILSK
PEKLLFIIDSLEGMECDLF----KWESELCDNCTEKQPVNVLLSSLLRRKMLPESSLLIS
ATPESFEKMENRIEYTHVKIIKGLKERNIKMSFHRLFQDRN---------RAHEAFSLVR
ENEQLFTVCQVPVLCWMVATCLKEEIEKGRDPVSICRCTTSLYTTHIFNLFIPQNAH--S
PSKKSQDQLQGLCSLAAEGMWT-DTFVFGEEALRRNGIMDSDIPILLDIGMLINIRE-SE
KSYIFLHPSVQEVCAAIFYLLKSHVDHPSQEVKS---------IEKLMFA-FLKKVKVQW
IFFGSFIFGLLHESEQKKLEAFFGHQLSQEIKRQLYQCLETISGN---------------
-----------EELQEQIDGMKLFYCLFEMDDDTFLVEAMNCME----------------
-QINFVAKDYSDVIVAA-HCLKHCFTLKKLSFSTQN---------VLSGAQEHS------
-----YMERLLTAWNHICSVFIISKDIQELRMKDTNLSESAFSVLYNNLKYH------NY
TLNVLVANNVFFVCEKYLFFELIQNC-NLQHLNLSLTILSH-SDVKLLCDVLSQAECNIE
ELV---------------------VAACSISSDDCKVFASVLISNKTLKHLNLASNTL-D
KGIASLCKSLCHPDCILEHLVLANCSLNEKCWDYLSEVLRRNKTLSHLDISSNDLKDEGL
KVL---------------------------------------------------------
-------------CRALTLPDSVLKSLSLRHCLITISGCQDLAEVLRNNQNLVSLQVSNN
KLEDTGVKLLCDAIKHPNCHLEDLGLEACELTGACCEDLASTFTQCKTLWAMNLLKNALD
YNGLVVLCEALKQQTCATYVLGLQITDFDTETQAFLVAEQEKNPCLRILSSL--------
------------------------------------------------------------
------------------------------------------------------------
------------------------------------------------------------
------------------------------------------------------------
-----------------

#NLRP4b_mouse "gi|27369660|ref|NP_766069.1| NLR family, pyrin domain containing 4B [Mus musculus]"
------------------------------------------------------------
------------------------------------------------------------
------------------------MASLFSDFGFIWYWKELNKIEFMYFKELLIHEILQM
GLKQIS-------WTEVKEASREDLAILLVKHCDGNQAWDTTFRVFQMIGRNVITNRATG
EIAA--------------------------------------------------------
-------------------------------------------------HSTIYRAHLKE
KLTHDCSRKFNISIQN-----FFQDEYDHLENLLVPNGTENNPKM--VVLQGVAGIGKTI
LLKNLMIVWSEGLVFQNKFSYIFYFCCHDVK-QLQTASLADLISREWPSPSAPMEEILSQ
PEKLLFIIDSLEGMEWNVT----QQDSQLCYNCMEKQPVNVLLSSLLRKKILPESSLLIS
TSCETFKDLKDWIEYTNVRTITGFKENNINMCFHSLFQDRN---------IAQEAFSLIR
ENEQLFTVCQAPVVCYMVATCLKNEIESGKDPVSICRRTTSLYTTHILNLFIPHNAQ--N
PSNNSEDLLDNLCFLAVEGMWT-DISVFNEEALRRNGIMDSDIPTLLDIGILEQSRE-SE
NSYIFLHPSVQEFCAAMFYLLHSEMDHSCQGVYF---------IETFLFT-FLNKIKKQW
VFLGCFFFGLLHETEQEKLEAFFGYHLSKELRRQLFLWLELLLDTL--------------
-----------HPDVKKINTMKFFYCLFEMEEEVFVQSAMNCRE----------------
-QIDVVVKGYSDFIVAA-YCLSHGSALTDFSISAQN---------VLNEELGQ-------
------RGKLLILWHQICSVFLRNKDIKTLRIEDTIFNEPVFKIFYSYLKNS------SC
ILKTLVAYNVSFLCDKRLFLELIQSY-NLEELYLRGTFLSH-SDVEMLCDILNQAECN--
------------------------------------------------------------
----------------IRILDLANCSLCEHSWDYLSDVLRQNKSLRYLNISYNNLKDEGL
```

```
KAL---------------------------------------------------------
-------------CRALTLPNSALHSLSLEACQLTGACCKDLASTFTRYKCLRRINLAKN
SLGFSGLFVLCKAMKDQTCTLYELKLRMADFDSDSQEFLLSEMERNKILSIENGV-----
------------------------------------------------------------
------------------------------------------------------------
------------------------------------------------------------
------------------------------------------------------------
------------------------------------------------------------
-----------------

#NLRP4c_mouse "Q3TKR3|NAL4C_MOUSE NACHT, LRR and PYD domains-containing protein
4C - Mus musculus"
------------------------------------------------------------
------------------------------------------------------------
------------------------MASFFSDFGLMWYLEELNKKEFMKFKEFLKQEILQL
RLKQIS-------WTEVKKASREDLANLLLKHYEEKKAWDMTFKIFQKMNRKDLMERAGR
EIAG--------------------------------------------------------
-------------------------------------------------HSKLYQAHLKK
KLTHDYARKFNIKVQDFSKQKFTQDDYDRFENFLISKVTAKKPHM--VFLQGAAGIGKSL
MLTKLMLAWSEGMVFQNKFSYIFYFCCQDVK-KMKRASLAELISKEWPKTSAPIEDILSQ
PEKLLFVIDNLEVMECDMS----ERESELCDTCTEKQPVRILLSSLLRRKMLPKSSFLIS
ATPETFEKMEGRVECTNVKIVTGFNESNIKMYFRSLFQDKT---------KTQEIFSLVK
ENQQLFTVCQVPVLCWMVATCLKKEIEKGRDLVSVCRRTTSLYTTHIFNLFIPQSAQ--Y
PSKESQAQLQSLCSLAAEGMWT-DTFVFGEEALRRNGIMDSDIPTLLDVRILEKSKK-SE
KSYIFLHPSIQEVCAAIFYLLKSHMDHPSQDVKS---------IEALIFT-FLKKVKVQW
IFFGSFIFGLLHESEQKKLEAFFGHQLSQEIKRQLYQCLETISGN---------------
-----------EELQEQVDGMKLFYCLFEMDDEAFLAQAMNCME----------------
-QINFVAKDYSDVIVAA-HCLQHCSTLKKLSLSTQN---------VLSEGQEHS------
-----YTEKLLMCWHHMCSVLISSKDIYILQVKNTNLNETASLVLYSHLMYP------SC
TLKALVVNNVTFLCDNRLFFELIQNQ-CLQHLDLNLTFLSH-GDVKLLCDVLSQEECNIE
KLM---------------------VAACNLSPDDCKVFASVLISSKMLKHLNLSSNNL-D
KGISSLSKALCHPDCVLKNLVLVNCSLSEQCWDYLSEVLRRNKTLNHLDISSNDLKDEGL
KVL---------------------------------------------------------
-------------CGALSLPDSVLKSLSVRYCLITTSGCQDLAEVLRKNQNLRNLQVSNN
KIEDAGVKLLCDAIKHPNCHLENIGLEACALTGACCEDLASAFTHCKTLWGINLQENALD
HSGLIVLFEALKQQQCTLHVLGLRITDFDKETQELLMAEEEKNPHLSILSSV--------
------------------------------------------------------------
------------------------------------------------------------
------------------------------------------------------------
------------------------------------------------------------
-----------------

#NLRP4d_mouse "Q66X24|Q66X24_MOUSE NALP-beta (Fragment) - Mus musculus"
------------------------------------------------------------
------------------------------------------------------------
------------------------MASFFSDVGLMWYLEELNRKEFIKFKEFLKQEILQL
GLKQIS-------WTEVKKASRKDLDNLQLKHYGEKQTWDMTFNIFQKLNKKELIEGAER
EIAG--------------------------------------------------------
-------------------------------------------------HPKLYRAHLKT
KVTHYGFRAFNISIQDFFRKEFTKDDFDYFEDLFLSKRTEMKPHVREVFLYGVAGIGKTL
LLKKFMFAWLEGLVIQDKFSYIFYFCCQDLK-QLKTASLADLISSECPNPSAPIEEILSQ
PEKLLFIIDSLEWIECDLS----EQESELCDNCMEKKPVNIQISSLLRGKILPQSSLLIS
ATPR-FEKMQDMIECTDVKIIKGFKESNINMYFHIMIRDNN---------KVHEAFLVR-
ENEQLFTICQAPVLCWMVITCLKNEIEKGRDPVSICQSTTSLYTTHIFNLFIPQSAQ--Y
PSKESQAQLQSLCSLATEGMWT-GTFMFSEEALRRNGTMDSDIPTLLDIGMLHDG-----
--------------------------HPSQHVKC---------IETLMFM-FLKKVKVQW
VFWG-FIFGLLHRALEEKLEIFFGYSLSKKIKDKLCQCLKTISRN---------------
-----------KSLQEEIDGMK-FFCLFELDDEAFLVQAMNCME----------------
-QIRFVTKDYSDIIIAA-YCLKHCSKLKKLSFSTEN---------VLIEELDQS------
-----YMEKVLDCWNHMCSGFTRSKDIKVLQIKDTHFKEQVFQVLFESLRYP------SF
TLKELMVNNVTFYDGEHMFFELIQNC-SLECLNLLSSLLSP-SDVELLCKILIQSECKME
```

K-----------------------VAHCKLSPDDCKSFAFVLMRSKSLKDLNLACNNL-G
QGISSLCKALCHQDCILKYLVLNNCSLSEQCWDYLSDVLSQNKTLRHLEISVNDLKDEGL
KIL---
-------------CKALTVLES----LSMVHCFITSSGCQDLAEVLRKKQKLKCLDVSNN
KLEDSGVKLLCEAMEHPNCHLEDLGLDDCDITGASSENLSSAFIQCKALKNINLNGNVFE
VSEMVFSDMLKQQKHSRLIFG-LRITDFDNESPAFLVFEEKKN-----------------

#NLRP4e_mouse "gi|51921379|ref|NP_001004194.1| NLR family, pyrin domain containing 4E [Mus musculus]"

------------------------MASFFSDFGLMWYLEELNKKEFMKFKELLQQEILQL
GLKHIS-------WTEVKKASREDLANLLLKHYEEKKAWDMTFKIFQKMNRQDLMERAGR
EIAG--
---HSKLYQVHLKK
KLTHDYARKFNIKDQDSLKQKFTQDDCDHFGNLLISKATGKKPHM--VFLQGVAGIGKSL
MLTKLMLAWSEGMVFQNKFSYIFYFCCQDVK-KMKRASLAELISKEWPNASAPIEEILSQ
PEKLLFVIDNLEVMECDMS----EWESELCDDCMEKQPVNILMSSLLRRKMLPESSFLVS
ATPETFEKIEDRIECTNVKMMAGFNESSIKVYFYSLFQDRN---------RTQEIFSLVR
ENEQLFSVCQVPVLCWMVATCLKKEIEKGRDPVSICRRITSLYTTYIFNLFIPHSAQ--Y
PSKKSQDQLQGLCSLAAEGMWT-DTFVFAEEALRRNGIMDSDISTLLDVRILEKSKE-SE
KSYIFLHPSIQEVCAAIFHLLKSHVDHPSQDVKS---------IEALIFT-FLKKVKIQW
IFFGSFIFGLLHESEQKKLEAFFGHQLSQEIKRQLYQCLETISGN---------------
-----------KELQEQIDGMKLFYCLFEMDDEAFLPQVMNCME----------------
-QIKFVAKDYSDVIVAA-HSLQHCSTLKKLSLSTQN---------ILSEGQEHS------
-----YTEKLLICWHHVCSVLTSSKDIHVLQVKDTNFNERAFLVLYSHLKYP------SC
ILKVLEVNNVTLLCDNRLLFELIQNQ-RLQLLNLSLTFLSH-NDVKLLCDVLNQAECNIE
KLM---------------------VADCNLSPDDCKVFVSVLISSKMLKHLNLSSNNL-D
KGISSLSKALCHPDCVLKNLVLAKCSLSEECWHYLSEVLRRNKTLTHLDISFNDLKDEGL
KVL---
-------------CGALTLPDSVLISLSVRYCLITTSGCQDLAEVLRNNQNLRNLQISNN
KIEDAGVKLLCDAIKHPNCHLENIGLEACALTGACCEDLASSFTHCKTLLGINLQENALD
HSGLVALFEAMKQQQCTVNLRGLRITDFDKETQEFLMAEKEKNPYLSI------------

#NLRP4f_mouse "gi|140972011|ref|NP_780499.3| NALP, kappa [Mus musculus]"

------------------------MASFISDFGLIWYLRELNKKEFMKFKDFLIQEILEL
KLKQVS-------STKVKKASREDLANLLLKCGE-NQAWDMTFRILQKINRKDLTERATG
AIVG--
---NPNLYRDHLKK
KLTHDCPKKFNVRIQDFIKETFIQNDYDAFENLLISKGTERKPHM--VFLKGMAGVGKTL
MLKNLMLAWSKGLVFQNKFSYAFYFCCQDVK-QLKTASLAELISREWPSPSAPIEEILSQ
PEKLLFIIDSLEGMEWDLT----KQESELCDDCMEKQPVSTLLSSLLRRKMLPESSLLLS
TTPETFEKMEDRIQCTDVKTATAFDERSMKIYFHRLFQDRK---------RAQEAFSLVR
ENKQLFTICQVPLLCWMVATCLKEEIEKGGDPVSLCRRTTSLYTTHIFSLFIPQSAQ--Y
PSKKSQDQLQGLCSLAAEGMWT-DTFVFGKEALRRNGIFDSDIPTLLDIGMLGKIRE-FE
NSYIFLHPSVQEVCAAIFYMLKRHVEHPSQDVKN---------IETVLFM-FLKKVKTQW
IFLGCFIFGLLQKSEQEKLGVFFGHRLSKNIHHKLYQCLETLSGN---------------
-----------AELQEQIDGMRLFSCLFEMEDEAFLVKAMNCMQ----------------
-QINFVAKNYSDFIVAA-YCLKHCSTLKKLSFSTEN---------VLNE-GDQS------

-----YMEELLICWNNMCSVFVRSKDIQELRIKDTNFNEPAIRVLYESLKYP------SF
TLNKLVANNVSFG-DNHVLFELIQNS-SLQYLDLSCSFLSH-NEVKLLCDILNQAECNIE
KLM---------------------IAHCKLSPDDCKIFGSILMSSKSLKVLNLASNNL-N
QGISSLCKALCHPHCTLEYLVLSNCSLSEQCWDYLSEVLRQNKTLSHLDISSNDLKDEGL
KIL---
-------------CRSLILPYCVLESLCLSCCGITERGCQDLAEVLKNNQNLKYLHVSYN
KLKDTGVMLLCDAIKHPNCHLKDLQLEACEITDASNEELCYAFMQCETLQTLNLMGNAFE
VSRMVFFPRF--

#NLRP4g_mouse "Q3UWL9|Q3UWL9_MOUSE In vitro fertilized eggs cDNA, RIKEN full-length enriched library, clone:7420493B02 product:similar to PAN2 protein - Mus musculus"

------------------------MASFISDFGLMWYLKELNKKEFIKFKEFLIQEILKL
KLKQIS-------WTEVKKASREDLANLLLKCYEENQAWDMTFNILQKINRKDLTERATE
EIADS---
--SPLGLTDSGNPKLYRDHLKK
KLTHDCSKKFNVRIQDFIKEIFIQNDCDTFENLLISKGTEKKPHM--VFLQGMAGIGKTM
MLKNLMLAWSKGLVFQNKFSYTFYFCCRDVK-QLKTASLAELISREWPSPSAPIEEILSQ
PEKLLFIIDSLEGMECDLT----KQESELCDNCMEKQPVSILLSSLLTRKMLPESSFLLS
TTPETFEKMEDRILCTDVKTATAFDERSIKIYFHRLFQDKI---------RAQEAFSLVR
ENEQLFTICQVPLLCWMVATCLKEEIEKGGDPVSVCRHITSLYTTHILNLFIPQSAQ--Y
PSKKSQDQLQGLCSLAAEGMWT-DTFVFGEEALRRNGILDSDIPTLLDIGMLGKIRE-FE
NYYIFLHPSVQEVCAAIFYLLKSHVDHPSQDVKS---------IETVLFM-FLKKVKTQW
IFLGCFIFGLLQKSEQEKLVVFFGRRLSQKIQHKLYQCLETISGN---------------
-----------AELQEQIDGMKLFCCLSEIEDEAFLVKVMNCMQ----------------
-QINFVAKNYSDLILAA-YCLKHCSTLKKLSFSTQN---------VLNEKGNQR------
-----CMKKLIICWNDMCSVFVRSKDIQVLQIKDTSFNEPAIRILYEYLKYP------SF
TLNKLVANNVHFFGDNHAFFELIQNC-SLQYLDLGCSFLTH-SEVKLLCDVLNQAECNIE
KLV---------------------VSHCKLSPDDCKIFGSVLMSSKTLKVLNWAYNNL-N
QGISLLCKAWCHPDCILEYLVLANCSLSEQCWDYLSEVLRQNKTLSHLDISSNDLKDKGL
KIL---
-------------CRALTLPYCALKSLYLNNCQITARGCQDLAKVLRNNQNLKCLHISNN
KLKDAGLMLLCKAIKHPNCHLEDLRLEACEITGASNEDLRYAFMQCETLQMINLMGNALE
IVDY--

#NLRP4_human "gi|19745162|ref|NP_604393.1| NLR family, pyrin domain containing 4 [Homo sapiens]"

-----------------------MAASFFSDFGLMWYLEELKKEEFRKFKEHLKQMTLQL
ELKQIP-------WTEVKKASREELANLLIKHYEEQQAWNITLRIFQKMDRKDLCMKVMR
ERTG--
---YTKTYQAHAKQ
KFSRLWSSKSVTEIHLYFEEEVKQEECDHLDRLFAPKETGKQPRT--VIIQGPQGIGKTT
LLMKLMMAWSDNKIFRDRFLYTFYFCCRELR-ELPPTSLADLISREWPDPAAPITEIVSQ
PERLLFVIDSFEELQGGLN----EPDSDLCGDLMEKRPVQVLLSSLLRKKMLPEASLLIA
IKPVCPKELRDQVTISEIYQPRGFNESDRLVYFCCFFKDPK---------RAMEAFNLVR
ESEQLFSICQIPLLCWILCTSLKQEMQKGKDLALTCQSTTSVYSSFVFNLFTPEGAE--G
PTPQTQHQLKALCSLAAEGMWT-DTFEFCEDDLRRNGVVDADIPALLGTKILLKYGE-RE

SSYVFLHVCIQEFCAALFYLLKSHLDHPHPAVRC---------VQELLVANFEKARRAHW
IFLGCFLTGLLNKKEQEKLDAFFGFQLSQEIKQQIHQCLKSLGER---------------
-----------GNPQGQVDSLAIFYCLFEMQDPAFVKQAVNLLQ----------------
-EANFHIIDNVDLVVSA-YCLKYCSSLRKLCFSVQN---------VFKKEDEHSS-----
-----TSDYSLICWHHICSVLTTSGHLRELQVQDSTLSESTFVTWCNQLRHP------SC
RLQKLGINNVSFSGQSVLLFEVLFYQPDLKYLSFTLTKLSR-DDIRSLCDALNYPAGNVK
ELA---------------------LVNCHLSPIDCEVLAGLLTNNKKLTYLNVSCNQL-D
TGVPLLCEALCSPDTVLVYLMLAFCHLSEQCCEYISEMLLRNKSVRYLDLSANVLKDEGL
KTL---
-------------CEALKHPDCCLDSLCLVKCFITAAGCEDLASALISNQNLKILQIGCN
EIGDVGVQLLCRALTHTDCRLEILGLEECGLTSTCCKDLASVLTCSKTLQQLNLTLNTLD
HTGVVVLCEALRHPECALQVLGLRKTDFDEETQALLTAEEERNPNLTITDDCDTITRVEI

#NLRP4_chimpanzee "gi|114679221|ref|XP_524404.2| PREDICTED: NACHT, leucine rich repeat and PYD containing 4 isoform 4 [Pan troglodytes]"

---MTLHL
ELKQIP-------WTEVKKASREELANLLIKHYEEQQAWNITLRIFQKMDRKDLCMKVMR
ERTG--
---YTKTYQAHAKQ
KFSRLWSSKSVTEIHLYFEEEVKQEECDHLDRLFAPKETGKQPRT--VIIQGPQGIGKTT
LLMKLMMAWSDNKIFRDRFLYTFYFCCRELR-ELPPTSLADLISREWPDPAAPITEIVSQ
PERLLFVIDSFEELQGSLN----EPDSDLCGDLMEKQPVQVLLSSLLRKKMLPEASLLIA
VKPVCPKELQDQVTISEIYQPRGFNESDRLVYFCCFFKDPK---------RAMEAFNLVR
ESEQLFSICQIPLLCWILCTSLKQEMQKGKDLALTCQSTTSVYSSFVFNLFTPEGAE--G
PTPQSQHQLKALCSLAAEGMWT-DTFEFCEDDLRRNGVVDADIPALLGTKILLKYGE-RE
SSYVFLHVCIQEFCAALFYLLKSHLDHPHPAVRC---------VQELLVANFEKARRAHW
IFLGCFLTGLLNKKEQEKLDAFFGFQLSQEIKQQFHQCLKSLGEH---------------
-----------GNPQGQVDSLAIFYCLFEMQDPAFVKQAVNLLQ----------------
-EANFHIIDNVDLVVSA-YCLKYCSSLRKLCFSVQN---------VFKKEDEHSS-----
-----TSDYSLVCWHHICSVLTTSGHLRELQVQDSTLSESTFVTWCNQLRHP------SC
RLQKLGINNVSFSGQSVLLFEVLFYQPDLKYLSFTLTKLSR-DDIRSLCDALNYPAGNVK
ELG---------------------LVNCHLSPIDCEVLAGLLTNNKKLTYLNVSCNQL-D
TGVPLLCEALCSPDTVLVYLMLAFCHLSEQCCQYISEMLLHNKSVRYLDLSANVLKDEGL
RTL---
-------------CEALKHPDCCLDSLCLVKCFITAAGCEDLASALISNQNLKILQVGCN
EIGDVGVQLLCRALTHPDCRLEILGLEECGLTSTCCKDLASVLTCSKTLQQLNLTLNTLD
RTGVVVLCEALRHPECALQVLGLRKTDFDEETQALLTAEEERNPNLTITDDCDTITRGRD
LIARNLGSDSNTCKGQGLGPLLT-------------------------------------

#NLRP4a_rat "gi|109458051|ref|XP_218188.4| PREDICTED: similar to NACHT, leucine rich repeat and PYD containing 4A [Rattus norvegicus]"

------------------------MASFFSDFGLMWYLEELNKKEFMKFKEFLKQEILQL
GLKQVS-------WTEVKKASREDLASLLLKHYEEKQAWDMTFNFFQKINRKDLIKRAKR
EIDG--
---YPKLYRAHLKT
KLTHDSSRIFNINIQDFLKEKFTKDDHDRFETLLQSKGTESKPQV--VVLSGVAGIGKTL
MLKRLMLAWIEGLVFPYKFSYIFYFCCQEVK-QLKTASLAELISREWPDPSAPIEEILSE
PEKLLFIIDSLEGIECDLF----KCDSELCDNCMEKQPVNILLSSLLRKKMLLEASLLIS

TTPETFEKMEDRIEYTHVKIITGLNESDIKMCFHRLFQDRN---------RAQEAFSLVR
ENEQMLTICQVPVLCWMVATCLKTEIEKGRNLVSTCRRTTSLYTTYIFNLFIPQSAH--S
PSKKSQDQLQGLCSLAAEGIWT-DTFVFTEEALRRNGILDSDIPTLLDIGMLGKMGK-FE
NCYIFLHPSLQEACAAIFYLLKSHGDHPIEDVKS---------VEALLFT-FLKKAKVQW
ILGGRFIFGLLHESEQKKLEAFFGHQLSQEIKHQLYQCLETISVN---------------
-----------EELQEQIDGMKLFYCLFEMEDEDFLMEAMNYME----------------
-QINFVAKDYSDVIVAA-YCLKHCYTLKKLSFSTQN---------ILNEEHEHS------
-----CMENLLTCWRHICSVLINSKDIQELQIKDTNLNEPAFSVLYNSLKYC------ND
TLKVLVENNVFFLCEKYLFFELIQNC-NLQHLNLNLTFLSH-SDVKLLCDVLNQAECNIE
KLE---------------------VAACNLSPDDCKMFASILMSSKTLKQLNLSSNIL-G
KGISSLCKSLCHPDCILEHLVLAKCSLSDQCWDYLSDGIRQNKTLNHLDISSNDLKDEGL
KVL---
-------------CRALALPNSVLKSLCLRHCLITTSGCQDLAEVLRNNQNLTSLQISYN
KIEDAGVKLLCDAIKQPNCHLEDLGLEACELTGACCEDLASTFTQCRTLWGINLLNNTLD
YTGLVVLCEALRQQKCTPHVLGLRITDFDNETQAFLVAEQEKNPDLSILSGV--------

#NLRP4b_rat "gi|109458152|ref|XP_344857.3| PREDICTED: similar to NACHT, leucine rich repeat and PYD containing 4B [Rattus norvegicus]"

------------------------MASLFSDFGFMWYWKELNKVEFMKFKELLILEILQM
GLKQIS-------WTEVKNASREDLAILLVKYCEGKQAWDTTFKVLQKISRHDLTERATG
EIAA--
---HANIYRAHLKK
KLTRDCSRKFSISIQN-----FFQDEYDHLENLLIPKVTEEKPQL--VFLKGIAGIGKTI
LLKNLMIVWSEGLVFQNKFSYIFYFCCQDVK-QLKTTSLTELISREWPSSSAPIEEILSQ
PEKLLFIIDSLEGMEWDFT----KQESELCDNCLEKQPVNVLLSSLLKKKILPESSLLIS
ATFETFEELKDWIEYTNVRTITGFKESNIKMYFHSLFQDRN---------RALEAFSFVR
ENEQLFTLCQAPVICCMVATCLKNEIEKGKDPVSICRRTTSLYTTHIFNLFIPPNVQ--Y
PSKKSQDQLQGLCFLAVEGMWT-DISVFSEETLRRNGILDSDIPTLLDIGILEQSRE-SQ
NSYIFFHPSVQEFCAAMFYLLQTHMNHPSPDVLH---------VEKLLFS-FLKEVNTQW
IFLGRFIFGLLNELEHEKLEAFFGYQLSQQLKQELFEWLELLLD----------------
-------------PEVKVNTMKFFYCLFEMEEEVFVQSAMNCRE----------------
-EIDVVAKDYYDFIVAA-YCLNHGSALRDLSISTQN---------VLNEKLNQR------
-----YMENLLMLWHNICSVFARNKDIHILQMKDTIFNEPVFQILYNYLKNS------SC
ILEVLVANDVSFLCDKYLFFDLIQSY-NLELLDLSGTFLSH-SDVVILCNILNKAEYK--

----------------IQELELANCSLSEQSWKYISDVLCQNKTLRHLDISSNDLKDEGL
KVL---
-------------CKALTLPDSVLLTLSLEACELTGACCEDLASTFTQCKTLGWINLVKN
ALDFNGLVVLCKALKQKTSNLKELRLRITDFDNKSQTFLLSEEKGNKFLNIENDE-----

#NLRP4c_rat "gi|109480334|ref|XP_234957.4| PREDICTED: similar to NACHT, leucine rich repeat and PYD containing 4A [Rattus norvegicus]"

------------------------MASFFSDFGIMWYLKELNKREFVKFKEFLKQEILQF
GLTIS--------CTEVKRATREGLANLLLKHYEEKQAWNITFNIFQKLNRTDLIERAKR
EIAG--
---HPKLYQAHLKT

```
KLTHDSSRVFTTIIQDLLKENFTQNDRAYFENLLPSNRTVMRPQV--VVIKGMAGIGKTL
ILKKLMFAWSKGLLFQNNFSYIFYFCCQDVK-QLKTASLAELISREWPGSSAPIEEILSE
PEKLLFLIDSLEGMEWDLS----ECESKLCANCIEMQPVNVVLSSLLMRKILPESSLIIS
TTPETFEKMENRIKYTSVRTVTGLNESDIISCFYNLFQDMN---------RALEVFSLVR
KNEQLFNICQAPIVCCMIAKCLKNEIEKGKDPISLCRHITSLYTTYIFNLFIPQNAQ--Y
PSKKSQDQMQGLCFLAAEGMWT-NTFVFSEESLRRNGVMDSDIPTLLDIGMLVKIRE-SE
SSYIFLHPSVQEVCAAIFYFLKSHEDHPSQYVKC---------IDILIFM-FLKKIKTEW
IFLGCFIFGLLHELEQKKLDAFFGHQLSQEIKHQFYQCLETIRGN---------------
-----------EELQEQIDGMKLFYCLFEMEDEAFLIQAMNCIE----------------
-QINFVAKDYSDIIVAA-YCLKHCSTLKKVSFSTQN---------ILNEEQEYS------
-----SMEKLLTYWNQMCSIFISNKDIQELQIKQTCLSDPAFSVFYNHWKHR------TC
TIKILKANMVSFLCEKHLFFEFIQNH-NLQYLDFSFTCLSH-INVELLCDVLNQAECNVE
KLV---------------------VEHCNLSPDDCQNFAFVLMNSKTLKILNLACNNL-D
KGIYSLCKALCHPNCILENLVLDNCSLSEQCWDYLSDALRQNKILCHLDISNNELKDKGL
KIL---------------------------------------------------------
-------------CKALTLPNCVLKSLCMTHCLITSSGCQDLAEVLRNNQNLKGLQISNN
KLEDAGVKLLCDAIKHPNCQLMDLGLEACEITGASSEDLSSAFLQSNTLGRVNLCGNAFE
ISGLALFPRF--------------------------------------------------
------------------------------------------------------------
------------------------------------------------------------
------------------------------------------------------------
------------------------------------------------------------
-----------------

#NLRP4d_rat "ENSRNOT00000041269 peptide:ENSRNOP00000051463
pep:NOVEL_protein_coding"
------------------------------------------------------------
------------------------------------------------------------
------------------------------------------------------------
------------------------------------------------------------
------------------------------------------------------------
------------------------------------------------------------
------------------------------------------------------------
------------------------------------------------------------
------------------------------------------------------------
------------------------------------------------------------
------------------------------------------------------------
-------------------VWT-NTFVFSEEDLRQNGIVDSDIPTLLDIGMLVKIRE-SE
DSYIFLYPSVQEVCAAIFYLLKSHEDHPSQYVKC---------IDILIFK-FLEKIKTQW
TLLGCFIVGLLHELEQKKLDAFFGHQLSQEIKHQFYQCLETIRGN---------------
-----------GELQEQIDGMKLFYCLFEMEDEAFLVQAMNCME----------------
-QINFVAKDYSDIIVAA-HCLEHCSTLKKVSFSTQN---------IRNEEQEYS------
-----YMEKLLTYWDQMRSIFISNKDIQELQIKQTCLSEPAFSIFYSHWKHH------SG
TIKILKANMVHFLWEKHPLFEFIQNH-NLQCLDFSFTCLSR-TNVKLLCDFLN-------
------------------------------------------------------------
-----------HPNCILENLVLDNCSLNEQCWDYLSDALRQNKILGHLDSSNNDLKDK-G
LKF---------------------------------------------------------
-------------CKALTLPNCVLKSPCMTHCLITSSGCQDLAEVLRNNQNLKDLQISNN
KLEDAGVKLLYDAIKHPNCHLVDLG-----------------------------------
------------------------------------------------------------
------------------------------------------------------------
------------------------------------------------------------
------------------------------------------------------------
------------------------------------------------------------
-----------------

#NLRP4e_rat "gi|62638853|ref|XP_574350.1| PREDICTED: similar to NACHT, leucine
rich repeat and PYD containing 4E [Rattus norvegicus]"
------------------------------------------------------------
------------------------------------------------------------
------------------------MASFFSDFGLMWYLEELNKKEFVKFKEFPMKEILQL
```

RLKQIS-------WTKVKKASREDLANLLLKHYEEKQAWDMTFKIFQKMNRKDLIERAGR
EIAG--
---HSKLYQAHLKK
KLTHDYARKFNIKAQDLFKQKFTQDDCDRFENLLVSKATGKKPHM--VFLQGVAGIGKSL
MLTKLMLAWSEGIVFQNKFSYIFYFCCQDVK-QLKRASLAELISREWPNASAPTAEILSQ
PEKLLFIIDSLEVMECNMS----ERESELCDNCTEKQPVSLLLSSLLRRKMLPESSFLIS
ATPETFEKMEDRIECTNVKIITGFNENNIKMYFRSLFQDKN---------RTLEAFSLVR
ENEQLFNVCQVPVLCWMVATCIKKEIEKGRDPVFICRRTTSLYTTHIFNLFTPQNAQ--Y
PSKKSQDQLQGLCSLAAEGMWT-DTFVFSEEALRRNGILDSDIPTLLDRRILERSKE-SE
SCYIFLHPSLQEVCAAVFYLLKSHLDHPSQDVKS---------VEALLFT-FLKKAKVQW
IFLGCFLFGLLHESEQEKLEMFFGHQLSQEIKHQLYQCLETISVN---------------
-----------EELQEQIDGMKLFYCLFEMEDEAFLMQAMNCME----------------
-QINFVAKDYSDVIVAA-YCLKHCSTLKKLSFSTQN---------ILSEEQEHS------
-----YTEKLLICWHHMCSVLISSKDIHVLQVKDTNLNETAFWVLYNHLKYP------SC
TLKVLVVNNVTFLCDNHLFFELIQNQ-RLQHLNLSLTFLSH-SDVKLLCDVLNQAECNIE
KLM---------------------IAACNLSPDDCKVFASVLISSKMLKHLNLSSNNL-D
KGISSLCKALCHPDCILKHLVLANCSLSEQCWDYLSEVVRRNKTLSHLDISSNDLKDEGL
KVL---
-------------CGALTLPDSGLISLSVRHCLITTSGCQDLAEVLRHNQNLRSLQVSNN
KIEDAGVKLLCDAIKQPNCHLENIGLEACELTGACCKDLASAFVHCKTLWGINLLENALD
HSGLVVLFEALKQQKCTLHVLGLRITDFDKETQEFLIAEEEKNPYLSILSNV--------

#NLRP4f_rat "gi|109458053|ref|XP_001076792.1| PREDICTED: similar to NACHT, leucine rich repeat and PYD containing 4A [Rattus norvegicus]"

------------------------MASFSSDFSLVWYLKELNQKEFMKFKDFLIQEILEL
KLKQIS-------WTEVKKASREGLSNLLLKCYGENQALDMTFKILQKINRKDLTKKATR
EIADS---
--STLGLTDSENSKLYREHLKK
KLSHNCSKMFNINIQDFILETVIQDDYDTFENLLVSKGDEKKPHM--VFLKGMAGIGKTL
MLKNVMLAWAKGLVFQNKFSYTFYFCCQDVK-QLKTASLAELISREWPISSAPIEEILSQ
PEKLLFIIDSLEGMEWDLT----KQESELCGNCTEKQPVSLLLSSLLRRKMLPESSFLIS
ATPETFEKMEDRIRCTDVKTATAFNERSIEICFHRLFQDRN---------TAQKAFSLVR
ENEQLFTLCQAPLLCWMVATCLKKEIEKGKDPVSICRHITSLYTTYIFNSFIPQSAK--Y
PSKKSQDQLQGLCSLAAEGMWT-DTFVFSEEALRRNGIMDSDIPTLLDIGMLGKIRE-FE
NSYLFLHPSVQEICAAIFYLLKSHGVHPSQDVKS---------VETVLFM-FLKKVKTQW
ILWGSFIFGLLHKSEQEKLAVFFGYQLSQKIRHKLYQCLETINGN---------------
-----------VELQEQIDGMKLFYCLFEMEDDAFLVKAVNCMQ----------------
-QINFVANYYSDFFVAA-YCLKRCSTLKKLSFSTQN---------ALNEELEQQ------
-----YRERLLICWNDICSVFVRSKNIQTLQIKDTNFSEPTFRVLYESLKYP------SF
PLSKLVANNVHFFGDNHMFFELIQNH-NLQYLDLSCSSLSP-NGVKLLFDVLNQAECNIE
KLM---------------------VAHCKLSPDDCKIFGSILMSSKTLKILNLASNNL-N
QGISSLCEGLCHPNCVLEYLVLANCSLSEQCWDYLSDVLRKNKTLNHLDISCNDLKDKGI
KIL---
-------------CKALTLPDCFMKSLCLKHCQITTRGCQDLAEVLRNNQNLKHLHVSNN
KLKDAGVKLLCDAIKHPNCHLVDLGLEACEITGASSENLSFAFIQCKTLKRLTLKGNAFE
ISGMVFPPKF--

#NLRP9_dog "gi|73947407|ref|XP_533578.2| PREDICTED: similar to NACHT, leucine rich repeat and PYD containing 9 protein [Canis familiaris]"

```
------------------------------------------------------------
------------------------------------------------------------
-----------------------MTESFFSDFGLLWYLEELKKEEFWKFKELLKQEPLQL
GLKPIP-------WTELKKASREDLAKMLDKHYPGKQAWDVTLNLFLQINRHDLWTKAQE
EIRN--------------------------------------------------------
-------------------------------------------------KPSPYRNHMKE
KFRLIWEKETCLPVPNDFYKETIKHEYENLHAAYRASQAEE--SSPTVVLQGPEGIGKTT
LLRKVMLEWAEGNLWKDRFTFIFFLNGCEMN-MITETSLVELISRDLPLSSEPVEDILSQ
PERILFIMDGFEELKFDLE----LS-TPLCNDQRQRQPMQIILSSLLQRKMLPESSLLIA
LGTEGMRKNYCLLQHPKYITLPGFSEHERKLYFYHFFRERN---------KALKAFSFVR
GNIPLFVFCHNPLVCWLVCTCMKWQLEKGEDLEIVSESTTSLYTSFFISVFQSRNET--C
PPKHSRTRLKGLCTLAAMGVWT-RMFVFCHEDLRRNGISESDTLMWMGMRILQRSG----
EYFTFTHMCLQEFCAAMFYVLKQPKDSPNPAIGS---------VTQLVTA-GVSQVQSPL
SRMITFLFAFSTEKITNLLETSFGFLLSKELKQEITQCLKSLSQCD--------------
-----------P-NQVAVNFQELFSGLFETHEKGFVAQVMDFFE----------------
-EVNIYIGNTEDLVISA-FCLKHCQNLRTLHLCIEN---------VFSDDS--GS-----
-----IINEKLSFWQDFCSVFTTNENFQMLDLDNCKFSEASLAILCKALAQP-----VCK
LQKYNFAS-DFG-SSPALHKAILHNP-HLKHLNLHGSSLSH-VDVRQLCEMLKHPTCSIE
ELM---------------------LGMCDITAEACEEIASVLVCNKKLKLLSLAENPLRN
EGMLMLCDALKHPDCVLETLLLMCCCLTSVACDYISQALLCNKSLSFLDLESNFLKDDGV
ASL---------------------------------------------------------
-------------CEALKHPNCHIEQLWLADCSLTSLCCKNLSDVLVCNEKLKILKLGSN
DIQDAGVKQLCEALKHPDCKVEHLGLDMCQLTTACCEALASALTVCKSLKSLNLHWISLD
RDGAVVLCEALNHLDCALQQLGLDKSVFDKEIQMLLTAVEEKNPHLTISHLLWINKEYRI
RGVLT-------------------------------------------------------
------------------------------------------------------------
------------------------------------------------------------
------------------------------------------------------------
-----------------

#NLRP9_bovine "gi|66954669|ref|NP_001019835.1| NLR family, pyrin domain
containing 9 [Bos taurus]"
------------------------------------------------------------
------------------------------------------------------------
-----------------------MAESFFSDFGLLWYLEELKKEEFWKFKELLKQEPLKL
KLKPIP-------WTELKKASRENVSKLLSKHYPGKLAWDVTLNLFLQISRDDLWRKARN
EIRQ--------------------------------------------------------
-------------------------------------------------KINPYRSHMKQ
KFQVLWEKEPCLLVPEDFYEETTKIEYELLSTVYLDAFKPG-ESSPTVVLHGPEGIGKTT
FLRKVMLEWAKGNLWRDRFSFVFFLTGREMN-GVTDMSLVELLSRDWPESSEPIEDIFSQ
PERILFILDGMEELKFDLD----CN-TDLCEDWEQPQSMQVVLQSLLQKQMLPECSLLLA
LSKMGMRKNYSLLKHMKCIFLLGFSEHQRKLYFSHYFQEKD---------ASSRAFSFVR
EKSSLFVLCQSPFLCWLVCTSLKCQLEKGEDLELDSETITGLYVSFFTKVFRSGSET--C
PLKQRRARLKSLCTLAAEGMWT-CTFLFCPEDLRRNGVSESDTSMWLDMKLLHRSG----
DCLAFIHTCIQEFCAAMFYMFTRPKDPPHSVIGN---------VTQLITR-AVSEHYSRL
SWTAVFLFVFSTERMTHRLETSFGFPLSKEIKQEITQSLDTLSQCD--------------
-----------P-NNVMMSFQALFNCLFETQDPEFVAQVVNFFK----------------
-DIDIYIGTKEELIICA-ACLRHCHSLQKFHLCMEH---------VFPDESGCIS-----
-----NTIEKLTLWRDVCSAFTASEDFEILNLDNCRFDEPSLAVLCRTLSQP-----VCK
LRKFVCNFASNLANSLELFKVILHNP-HLKHLNFYGSSLSH-MDARQLCEALKHPMCNIE
ELM---------------------LGKCDITGEACEDIASVLVHNKKLNLLSLCDNALKD
DGVLVLCEALKNPDCALEALLLSHCCFSSAACDHLSQVLLYNRSLTFLDLGSNVLKDEGV
TTL---------------------------------------------------------
-------------CESLKHPSCNLQELWLMNCYFTSVCCVDIATVLIHSEKLKTLKLGNN
KIYDAGAKQLCKALKHPKCKLENLGLEACELSPASCEDLASALTTCKSLTCVNLEWITLD
YDGAAVLCEALVSLECSLQLLGLNKSSYDEEIKMMLTQVEEMNPNLIISHHLWTDDEGRR
RGILV-------------------------------------------------------
------------------------------------------------------------
------------------------------------------------------------
------------------------------------------------------------
-----------------
```

#NLRP11_human "gi|21450725|ref|NP_659444.1| NLR family, pyrin domain containing 11 [Homo sapiens]"

```
------------------------------------------------------------
------------------------------------------------------------
-----------------------MAESDSTDFDLLWYLENLSDKEFQSFKKYLARKILDF
KLPQFP-------LIQMT---KEELANVLPISYEGQYIWNMLFSIFSMMRKEDLCRKIIG
RRNR--------------------------------------------------------
-------------------------------------------------NQEACKAVMRR
KFMLQWESHTFGKFHYKFFRDVSSDVFYILQLAYDSTSYYSANNLN-VFLMGERASGKTI
VINLAVLRWIKGEMWQNMISYVVHLTSHEIN-QMTNSSLAELIAKDWPDGQAPIADILSD
PKKLLFILEDLDNIRFELN----VNESALCSNSTQKVPIPVLLVSLLKRKMAPGCWFLIS
SRPTRGNNVKTFLKEVDCCTTLQLSNGKREIYFNSFFKDRQ---------RASAALQLVH
EDEILVGLCRVAILCWITCTVLKRQMDKGRDFQLCCQTPTDLHAHFLADALTSEAGL--T
ANQYHLGLLKRLCLLAAGGLFL-STLNFSGEDLRCVGFTEADVSVLQAANILLPSNT-HK
DRYKFIHLNVQEFCTAIAFLMAVPNYLIPSGSRE---------YKEKREQ------YSDF
NQVFTFIFGLLNANRRKILETSFGYQLP-MVDSFKWYSVGYMKHLD--------------
-----------RDPEKLTHHMPLFYCLYENREEEFVKTIVDALM----------------
-EVTVYLQSDKDMMVSL-YCLDYCCHLRTLKLSVQRIFQ------NKEPLIRPTA-----
-----SQMKSLVYWREICSLFYTMESLRELHIFDNDLNGISERILSKALEHS------SC
KLRTLKLSYVSTASGFEDLLKALARNRSLTYLSINCTSISL-NMFSLLHDILHEPTCQIS
HLS---------------------LMKCDLRASECEEIASLLISGGSLRKLTLSSNPLRS
DGMNILCDALLHPNCTLISLVLVFCCLTENCCSALGRVLLFSPTLRQLDLCVNRLKNYGV
LHV---------------------------------------------------------
-------------TFPLLFPTCQLEELHLSGCFFSSDICQYIAIVIATNEKLRSLEIGSN
KIEDAGMQLLCGGLRHPNCMLVNIGLEECMLTSACCRSLASVLTTNKTLERLNLLQNHLG
NDGVAKLLESLISPDCVLKVVGLPLTGLNTQTQQLLMTVKERKPSLIFLSETWSLKEGRE
IGVTPASQPGSIIPNSNLDYMFFKFPRMSAAMRTSNTASRQPL-----------------
------------------------------------------------------------
------------------------------------------------------------
------------------------------------------------------------
-----------------
```

#NLRP11_chimpanzee "gi|114679217|ref|XP_524402.2| PREDICTED: NACHT, leucine rich repeat and PYD containing 11 [Pan troglodytes]"

```
------------------------------------------------------------
------------------------------------------------------------
-----------------------MAESDSTDFDLLWYLENLSDKEFQSFKKYLARKILDF
KLPQFP-------LIQMT---KEELANVLPISYEGQYIWNMLFSIFSMMRKEDLCRKIIG
RRNR--------------------------------------------------------
-------------------------------------------------NQEACKAVMRR
KFMLQWESHTFGKFHYKFFRDVSSDVFYILQLAYDSTSYYSANNLN-VFLMGERASGKTM
VINLAVLRWIKGEMWQNMISYVVHLTSHEIN-QMTNSSLAELIAKDWPDGQAPIADILSD
PKKLLFILEDLDNIRFELN----VNESALCSNSTQKVPIPVLLVSLLKRKMAPGCWFLIS
SRPTRGNNVKSFLKEVDCCTTLQLSKEKREIYFNSFFKDRQ---------RASAALQLVH
EDEILVGLCRVAILCWITCTVLKRQMDKGRDFQLCCQTPTDLHAHFLADALTSEAGL--T
ANQYHLGLLKRLCLLAAGGLFL-STLNFSGEDLRCVGFTEADVSVLQAANILLPSST-HK
DRYKFIHLNVQEFCTAIAFLMAVPNYLIPSGSRE---------YKEKREQ------YSDF
NQVFTFIFGLLNANRRKILETSFGYQLP-MVDSFKWYSVGYMKYLD--------------
-----------RDPEKLTHHMPLFYCLYENREEEFVKTIVDALM----------------
-EVTVYLRSDKDMMVSL-YCLDYCCHLRTLKLSVQHIFQ------NKEPLIRPTA-----
-----SQMKSLVYWREICSLFYTMESLWELHIFDNDLNGISERILSKALEHS------SC
KLRTLKLSYVSTASGFEDLLKALARNRSLTYLSINCTSISL-NMFSLLHDILHEPTCQIS
HLS---------------------LMKCDLRASECEEIASLLISGGSLRKLTLSSNPLRS
DGMNILCDALLHPNCTLISLVLVFCCLTENCCSALGRVLLFSPTLRQLDLCVNRLKNYGV
LHV---------------------------------------------------------
-------------TFPLLFPTCQLEELHLSGCFFSSDICQYIAIVIATNEKLRSLEIGSN
KIEDAGMQLLCGGLRHPNCMLVNIGLEECMLTSACCRSLASVLTTNKTLERLNLLQNHLG
NDGVAKLLESLISPDCVLKVVGLPLTGLNTQTQQLLMTVKERKPSLTFLSETWSLKEGRE
IGVTPASQPGSIIPNSNLDYMFFKFPRMSAAMRMSNTASRQPL-----------------
------------------------------------------------------------
```


#NLRP13_human "gi|110624785|ref|NP_789780.2| NACHT, leucine rich repeat and PYD containing 13 [Homo sapiens]"

-----------------MNFSVITCPNGGTNQGLLPYLMALDQYQLEEFKLCLEPQQLMD
FWSAPQGHFPRIPWANLRAADPLNLSFLLDEHFPKGQAWKVVLGIFQTMNLTSLCEKVRA
EMK---------------------------------------ENVQTQELQDPTQEDLEM
LEAAAGNMQTQGCQDPNQEELDELEEETGNVQAQGCQDPNQEEPEMLEEA-DHRRKYREN
MKAELLETWDNISWPKDHVYIRNTSKDEHEELQRLLDPNRTRAQAQTIVLVGRAGVGKTT
LAMQAMLHWANGVLFQQRFSYVFYLSCHKIR-YMKETTFAELISLDWPDFDAPIEEFMSQ
PEKLLFIIDGFEEIIISESRSESLDDGSPCTDWYQELPVTKILHSLLKKELVPLATLLIT
IKTWFVRDLKASLVNPCFVQITGFTGDDLRVYFMRHFDDSS---------EVEKILQQLR
KNETLFHSCSAPMVCWTVCSCLKQPKVRYYDLQSITQTTTSLYAYFFSNLFSTAEVD--L
ADDSWPGQWRALCSLAIEGLWS-MNFTFNKEDTEIEGLEVPFIDSLYEFNILQKIND-CG
GCTTFTHLSFQEFFAAMSFVLEEPREFPPHSTKP--------QEMKMLLQHVLLDKEAYW
TPVVLFFFGLLNKNIARELEDTLHCKISPRVMEELLKWGEELGKA---------------
-----------ESASLQFHILRLFHCLHESQEEDFTKKMLGRIF----------------
-EVDLNILEDEELQASS-FCLKHCKRLNKLRLSVSSHILERDLEILETS-----------
-----KFDSRMHAWNSICSTLVTNENLHELDLSNSKLHASSVKGLCLALKNP------RC
KVQKLTCKSVTPEWVLQDLIIALQGNSKLTHLNFSSN-KLGMTVP-LILKALRHSACNLK
YLC---------------------LEKCNLSAASCQDLALFLTSIQHVTRLCLGFNRLQD
DGIKLLCAALTHPKCALERLELWFCQLAAPACKHLSDALLQNRSLTHLNLSKNSLRDEGV
KFL---
-------------CEALGRPDGNLQSLNLSGCSFTREGCGELANALSHNHNVKILDLGEN
DLQDDGVKLLCEALKP-HRALHTLGLAKCNLTTACCQHLFSVLSSSKSLVNLNLLGNELD
TDGVKMLCKALKKSTCRLQKLG--------------------------------------

#NLRP13_chimpanzee "gi|114679233|ref|XP_524405.2| PREDICTED: NACHT, leucine rich repeat and PYD containing 13 isoform 2 [Pan troglodytes]"

-----------------MNFSVITCPNGGTNQGLLPYLMALDQYQLEEFKLCLEPQQLMD
FWSAPQGHFPRIPWANLRAADPLNLSFLLDEHFPKGQAWKVVLGIFQTMNLTSLCEKVRA
EMK---------------------------------------ENVQTQELQDPTQEDLEM
LEAAAGNMQTQGCQDPNQEELEELEEETGNVQAQGCQDPNQEEPEMLEEA-DHRRKYREN
MKAELLETWDNISWPKDHVYIRNTSKDEHEELQRLLDPNRTRAQAQTIVLVGRAGVGKTT
LAMQAMLHWANGVLFQQRFSYVFYLSCHKIR-YMKETTFAELISLDWPDFDAPIEEFMSQ
PEKLLFIIDGFEEIIISESRSESLDDGSPCTDWYQELPVTKILHSLLKKELVPLATLLIT
IKTWFVRDLKASLVNPCFVQITGFTGDDLRVYFMRHFDDSS---------EVEKILQQLR
KNETLFHSCSAPMVCWTVCSCLKQPKVRYYDLQSITQTTTSLYAYFFSNLFSTAEVD--L
ADDSWPGQWRALCSLAIEGLWS-MNFTFNKEDTEIEGLEVPFIDCLYEFNILQKIND-CG
GCTTFTHLSFQEFFAAMSFVLEEPREFPPHSTKP--------QEMKMLLQHVLLDKEAYW
TPVVLFFFGLLNKNIARELEDTLHCKISPRVMEELLKWGEELGKA---------------
-----------ESASLQFHVLRLFHCLHESQEEDFTKKMLGRIF----------------
-EVDLNILEDEELQASS-FCLKHCERLNKLRLSVSSHILERDLEILETS-----------
-----KFDSRMHAWNSICSTLVTNENLHELDLSNSKLHASSVKGLCLALKNP------RC
KVQKLTYKSVTPEWVLQDLIIALQGNSKLTHLNLSSN-KLGMTVP-LILKALRHSACNLK
YLC---------------------LEKCNLSAASCQDLALFLTSIQHVTRLCLGFNRLQD
DGIKLLCAALTHPKCALERLELWFSQLAAPACKHLSDALLQNRSLTHLNLSKNSLRDEGV
KFL---
-------------CEALGRPDGNLQSLNLSGCSFTREGCRELANALSHNHNVKILDLGEN
DLQDDGVKLLCEALKP-HRALHTLGLAKCNLTTACCQHLFSVLSSSKSLVNLNLLGNELD

```
TDGVKMLCKALKKSTCRLQKLG--------------------------------------
------------------------------------------------------------
------------------------------------------------------------
------------------------------------------------------------
------------------------------------------------------------
-----------------

#NLRP13_dog "gi|73947403|ref|XP_541402.2| PREDICTED: similar to NACHT, leucine
rich repeat and PYD containing 13 [Canis familiaris]"
------------------------------------------------------------
------------------------------------------------------------
-----------------MSSSANIYVDNGSYDKLLSYLMGLDQSQLEEFKLCLQSPQLLL
EN------FRKIPWANLKAIDPINLLCLLSEYFSEKQMWEVTLSIFENMNLTSLCMEIGA
TMN---------------------------------------GEWQKQETKRYTQVIVGS
VESIIGHFQPRYLNIPS-------LFVSEMEQALGPQDPNQEAAEMPEEEKVHRRRYRER
MKAKILMMWDNMPWPEDHIYLRNVTEKEHDELKSLLYPNRTGAQPQTIILEGIAGVGKTT
LAMKAMLHWAEGFLFQQRFSYVFFISCHKVK-DMKDTTFAGLLSWDWPDSQVPIEELMSH
PERLLFVIDGFEEMDMPSN----LDNSPPCTDWYQQLPVNRILFYLLKKELVPMATLLIT
TKEYRTKDLKKLLLNPWFVQILGFTEGDREEYFIRYFGDQK---------KAKKVLYWVR
KNETLFYFCSAPLVCWTVCSCLKRQMVRNPHFQISTQTTTSLYVHFFSSLFATAEVS--L
SEQNWPGQWSALCSLAAEGMWF-RNFTFTKEDLKHKCLEAHLIGSLLRLNILRKVND-CE
ECVTFTHQSFQVFLGAMFYVLWGTKGSIGGPAKH--------EEMRVLLNNALVDRNFYW
NQMALFFFGLLKRSLIRELEDTLCCKVGQRIMDELLEWAEELKSY---------------
-----------DIVLNRFEFLHFFSCLYETQEENFVRQILSHLL----------------
-EADINIFGNQQLQVSS-FCLKHCRSLSKLRLSVTSSILQTELTFSVET-----------
-----LEPHSKIYQWQDICSVFCNGHVSELDLSNSKLNTSSMKKLCYELRNP------RC
KLRKLTCKSITPVKILKELVLVLHGNNRLTHLNLSSN-NLGVPVSTMIFKTLRHSACNLQ
YLC---------------------LEKCNLSAAIYQDLALHVSSTQRTTRLCLGFNPLQD
DGVRLLCAALTHPECALERLVLWFCQLGAPSCRYLSEALLKNKSLTHLNLRRNKLRDEGV
KFL---------------------------------------------------------
-------------CKALSHPDCSLQNLDLSDCSFTADGCQELANALRHNHNMKILDIRNN
DVQDNGVKHLCEVLQDPYCELNTLGLEKCNLTPACCQHLSSVLTSSKSLINLNLLENDLE
PSGVSILWKALKKSMCKLQKLG--------------------------------------
------------------------------------------------------------
------------------------------------------------------------
------------------------------------------------------------
------------------------------------------------------------
-----------------

#NLRP13_bovine "gi|119925439|ref|XP_608304.3| PREDICTED: similar to NACHT,
leucine rich repeat and PYD containing 13 [Bos taurus]"
------------------------------------------------------------
------------------------------------------------------------
-----------------MASPAGISWHDGARDQLLSHLRGLDPWQLEDFKLALQCPELLP
EG------ARRIPWADLRAAGPADLLCLLEERFPGRRTWEAALRVFEDLRLSSLCERMRA
ELHGWFCILTVGPGAAGMATRRQHFFLPNSGYLGNQSLRTHPSSCHTALQRELSSGCFCI
LAVGPGAAVIVVLRELVMDVLNQYIVEPEMSQVCRLQDPNQEEPETLEET-AHRRRYRER
LRTRILALWDRTPWPEDHIYLRHVTQREHAELRGLLRPRGAGAPPLTMLLEGAAGVGKTT
LATKLVLHWAEGVLFRGRFSYVFYISGHWLA-KLGDISFAGLLALDWPDSQVPVEEFRAH
PERLLFVIDGAEEVTLGSK----GSASRPGADWYQELPAASILVRLLKKELVPEATLLVT
AGPPGGRALRGLLLRPCRVDIPGFTEGDRREYLRRFLGDPD---------VAEEAWRRMQ
GSETLVRLCAAPLACWAVCAGLKRQLAGSPAPAPGAPTPTGLYAQLFCSLLAGAEPG--L
RTGSSAGQWRAFCSLAAEGLWL-AAFTFAGDALERWRLEAPFIDGLLRLQILRRVSD-CE
HCVTFAHRSFQEFFGALFYVLWGAQGSLGGVPRH--------QEMRRWLNHAFADANPYW
RQMVRFFFGLLGTDLARQLEEAVGCRMSPGVADEVLDWAEELERC---------------
-----------GAVSGRFDFLWLFQCLHETQDENLARRVLNRLP----------------
-VADLDIQGCEHLRVSS-FCLKHCQKLRKLRLSVSHRVLEKKRTSGLGTPE---------
----MRVADFRMHQWEDICSVFCSGNLSQLDLSNSKLSTASMMRLCLKLGNP------QC
RLQKLAWKSMAPVEGLRKLGLLLRGDRHLTHLDLSSN-SLDAAVSRGVFRMLGHSACGLK
YLW---------------------LEKGNLSAAACEDLASLLTSTPRLTRLCLGLNPLGD
EGVQLLCGSLTRPECVLQRLELWCCRLSTPSCRHLSDALLRSRSLTHLNLRRNSLGDGGV
```

```
KLL---------------------------------------------------------
-------------SSALGRADCALQSLNLSHCSLTVAGCRELAHALKHNGHLKILDVGNN
DIQDEGVKELCSVLKSPSCVLQTLGLEKCSLTAACCRPLSSVLGSSKSLENLNLLGNNLG
PDGVSRLWMPLRKPTCKLRKLG--------------------------------------
------------------------------------------------------------
------------------------------------------------------------
------------------------------------------------------------
------------------------------------------------------------
-----------------

#NLRP8_human "gi|33667040|ref|NP_789781.2| NLR family, pyrin domain containing 8 [Homo sapiens]"
------------------------------------------------------------
----------------------------------------------------MSDVNPPS
DTPIPFSSSSTHSSHIPP-WTFSCYPGSPCENGVMLYMRNVSHEELQRFKQLLLTELS-T
GTMPIT-------WDQVETASWAEVVHLLIERFPGRRAWDVTSNIFAIMNCDKMCVVVRR
EINAILPTLEPEDLNVGETQVNLEEGE---------------------------------
-----------------------------------------------SGKIRRYKSNVME
KFFPIWDITTWPGNQRDFFYQGVHRHEEYLPCLLLPKRPQGRQPK-TVAIQGAPGIGKTI
LAKKVMFEWARNKFYAHKRWCAFYFHCQEVN-QTTDQSFSELIEQKWPGSQDLVSKIMSK
PDQLLLLLDGFEELTSTLID----RLEDLSEDWRQKLPGSVLLSSLLSKTMLPEATLLIM
IRFTSWQTCKPLLKCPSLVTLPGFNTMEKIKYFQMYFGHTE---------EGDQVLSFAM
ENTILFSMCRVPVVCWMVCSGLKQQMERGNNLTQSCPNATSVFVRYISSLFPTRAEN--F
SRKIHQAQLEGLCHLAADSMWH-RKWVLGKEDLEEAKLDQTGVTAFLGMSILRRIAG-EE
DHYVFTLVTFQEFFAALFYVLCFPQRLKNFHVLS--------HVNIQRLIASPRGSKSYL
SHMGLFLFGFLNEACASAVEQSFQCKVSFGNKRKLLKVIPLLHKC---------------
-----------DPPSPGSGVPQLFYCLHEIREEAFVSQALNDYH----------------
-KVVLRIGNNKEVQVSA-FCLKRCQYLHEVELTVTLNFMNVWKLSSSSHPGSEAP-----
-----ESNGLHRWWQDLCSVFATNDKLEVLTMTNSVLGPPFLKALAAALRHP------QC
KLQKLLLRRVNSTMLNQDLIGVLTGNQHLRYLEIQHVEVES-KAVKLLCRVLRSPRCRLQ
CLR---------------------LEDCLATPRIWTDLGNNLQGNGHLKTLILRKNSLEN
CG------AYYLSVAQLERLSIENCNLTQLTCESLASCLRQSKMLTHLSLAENALKDEGA
KHI---------------------------------------------------------
-------------WNALPHLRCPLQRLVLRKCDLTFNCCQDMISALCKNKTLKSLDLSFN
SLKDDGVILLCEALKNPDCTLQILELENCLFTSICCQAMASMLRKNQHLRHLDLSKNAIG
VYGILTLCEAFSSQKKREEVIFCIPAWTRITSFSPTPHPPDFTGKSDCLSQINP------
------------------------------------------------------------
------------------------------------------------------------
------------------------------------------------------------
------------------------------------------------------------
-----------------

#NLRP8_chimpanzee "gi|114679237|ref|XP_512922.2| PREDICTED: NACHT, leucine rich repeat and PYD containing 8 [Pan troglodytes]"
------------------------------------------------------------
----------------------------------------------------MSDVNPPS
DTPIPFSSSSTHSSHIPP-WTFSCYPGSPCENGVMLYMRNVSHEELQRFKQLLLTELS-T
GTMPIT-------WDQVETASWAEVVHLLIERFPGRRAWDVTSNIFAIMNCDKMCVLVRR
EINAILPTLEPEDLNVGETQVNLEEGE---------------------------------
-----------------------------------------------SGKIRRYKSNVME
KFFPIWDFTTWPGNQRDFFYQDVHRHEEYLPCLLLPKRPQGRQPK-TVVIQGAPGIGKTI
LAKKVMFEWARNKFYAHKRWCAFYFHCQEVN-QMTDQSFSELIEQKWPGSQDLVSKIMSK
PDQLLLLLDGFEELTSTLID----RLEDLSEDWRQKLPGSVLLSSLLSKTMLPEATLLIM
IRFSSWQTCKPLLKCPSLITLPGFNTMEKIKYFQMYFGHTE---------EGDRVLSFAM
ENTILFSMCRVPVVCWMVCSGLKQQMERGNNLTHACPNATSVFVRYISSLFPTRAEN--F
SRKIHQAQLEGLCHLAADSMWH-RKWVLGKEDLEEAKLDQTGVTAFLGMSILRRIAG-EE
DHYVFTLVTFQEFFAALFYVLCFPQRLKNFHVLS--------HVNIQRLIASPRGSKSYL
SHMGLFLFGFLNEACASAVEQSFQCKVSFGNKRKLLKVIPLLHKC---------------
-----------DPPSPGSGVPQLFYCLHEIREEAFVSQALNDYH----------------
-KVVLRIGNNKEVQVSA-FCLKRCQYLHEVELTVTLNFMNVWKLSSSSPPGSEAP-----
-----ESNGLHRWWQDLCSVFATNDKLEVLTMTNSVLGPPFLTALAASLRHP------QC
```

KLQKLLLRRVNSTMLNQDLIGVLMGNQHLRYLEIQHVEVES-KAVKLLCRALRSPRCRLQ
CLR---------------------LEDCLATPRIWTDLGNNLQGNGHLKTLILRKNSLEN
CG------AYYLSMAQLERLSIENCNLTQLTCESLASCLRQSKMLTHLSLAENALKDEGA
KHI---
-------------WNALPHLRCPLQRLVLRKCDLTFNCCQDMISALCKNKTLKSLDLSFN
SLKDDGVILLCEALKNPDCTLQILELENCLFTSICCQAMASMLRKNQHLRHLDLSKNAIG
VYGILTLCEAFSSQKKREEVIFCIPAWTRITSFSPTPHPPDFTGKSDCLSQINPYAVQSS
FSGA--

#NLRP5_human "gi|114842393|ref|NP_703148.3| NACHT, LRR and PYD containing protein 5 [Homo sapiens]"

-----------------------------MKVAGGLELGAAALLSASPRALVTLSTGPTC
SILPKNPLFPQNLSSQPCIKMEGDKSLTFSSYGLQWCLYELDKEEFQTFKELLKKKSSES
TTCSIP-------QFEIENANVECLALLLHEYYGASLAWATSISIFENMNLRTLSEKARD
DMKRHSPEDPEA----TMTDQGPSKEKVPGISQAVQQDSATAAETKEQEISQAMEQEGAT
AAETEEQ--------------------------------------GHGGDTWDYKSHVMT
KFAEEED-----VRRSFENTAADWPEMQTLAGAFDSD-RWGFRPR-TVVLHGKSGIGKSA
LARRIVLCWAQGGLYQGMFSYVFFLPVREMQ-RKKESSVTEFISREWPDSQAPVTEIMSR
PERLLFIIDGFDDLGSVLN-----NDTKLCKDWAEKQPPFTLIRSLLRKVLLPESFLIVT
VRDVGTEKLKSEVVSPRYLLVRGISGEQRIHLLLERGIGEH---------QKTQGLRAIM
NNRELLDQCQVPAVGSLICVALQLQDVVGESVAPFNQTLTGLHAAFVFHQLTPRGVVRRC
LNLEERVVLKRFCRMAVEGVWN-RKSVFDGDDLMVQGLGESELRALFHMNILLPDSH-CE
EYYTFFHLSLQDFCAALYYVLEGLEIEPALCPLY-------VEKTKRSMELKQAGFHIHS
LWMKRFLFGLVSEDVRRPLEVLLGCPVPLGVKQKLLHWVS-LLGQQ--------------
-----------PNATTPGDTLDAFHCLFETQDKEFVRLALNSFQ----------------
-EVWLPINQNLDLIASS-FCLQHCPYLRKIRVDVKGIFPRDESAEACP-VVPLWM-----
----RDKTLIEEQWEDFCSMLGTHPHLRQLDLGSSILTERAMKTLCAKLRHP------TC
KIQTLMFRNAQITPGVQHLWRIVMANRNLRSLNLGGTHLKE-EDVRMACEALKHPKCLLE
SLR---------------------LDCCGLTHACYLKISQILTTSPSLKSLSLAGNKVTD
QGVMPLSDALRVSQCALQKLILEDCGITATGCQSLASALVSNRSLTHLCLSNNSLGNEGV
NLL---
-------------CRSMRLPHCSLQRLMLNQCHLDTAGCGFLALALMGNSWLTHLSLSMN
PVEDNGVKLLCEVMREPSCHLQDLELVKCHLTAACCESLSCVISRSRHLKSLDLTDNALG
DGGVAALCEGLKQKNSVLARLGLKACGLTSDCCEALSLALSCNRHLTSLNLVQNNFSPKG
MMKLCSAFACPTSNLQIIGLWKWQYPVQIRKLLEEVQLLKPRVVIDGSWHSFDEDDRYWW
KN--

#NLRP5_chimpanzee "gi|114679239|ref|XP_001139299.1| PREDICTED: NACHT, LRR and PYD containing protein 5 [Pan troglodytes]"

------------------MTHGSNPLPSLLSAAMLLAIRCSRTLPGVERNLVTLSTGPTC
SILPKNPLFPQNLSSQPCIKMEGDKSLTFSSYGLQWCLYELDKEEFQTFKELLKKKSSES
TTCSIP-------QFEIENANVECLALLLHEYYGASLAWATSISIFENMNLRTLSEKARD
DMKRHSSEDPEA----TTTDQGPSKEKVPGISQAVQQDSATAAETKEQEISQAMEQEGAT
AAETEEQEISEAEEAGTIEAKRQ----------------------GHGGDTWDYKSHVMT
KFAEEQD-----VRRSFEKSAADWPEMQTLAGAFNSD-QWGFRPR-TVVLHGKSGIGKSA
LARRIVLCWAQGGLYQGMFSYVFFLPVREMQ-RKKESSVTEFISREWPDSQAPVTEIMSR
PERLLFIIDGFDDLGSVLN-----SDTKLCKDWAEKQPPSILIRSLLRKVLLPESFLIIT
VRDVGIEKLKSEVVSPRYLLVRGISGEQRIHLLLERGIGEH---------QKTQGLRAIM
NNRKLFDQCQVPAVGSLICVALQLQDVVGESVAPFNQTLTGLYAAFVFHQLTPRGVARRC
LNLEERVVLKRFCRMAVEGVWN-RKSVFDGDDLMVQGLGESELRALFHMNILLPDSH-CE
EHYTFFHLSLQDFCAALYYVLEGLEIEPALCPLY-------VEKTKRSMELKQAGFHIHS
LWMKRFLFGLVSEDVRRPLEVLLGCPVPLGVKQKLLHWVS-LLGQQ--------------

```
-------------QKAPGDTLDAFHCLFETQDKEFVRLALNGFQ----------------
-EVWLPINQNLDLIASS-FCLQHCPYLRKIRVDVKGIFPRDESAEACP-VVPLWM-----
----RGKTLIEEQWEDFCSVLGTHPHLRQLDLGSSILTERAMKTLCAKLRHS------TC
KIQTLMFRNAQITAGVQHLWRILMANRNLRSLNLGGTHLKE-EDVRMACEALKHPKCLLE
SLR---------------------LDCCGLTHACYLKISQILTTSPSLKSLSLAGNKVTD
QGVTPLSDALRVSQCALQKLILEDCGITATGCQSLASALVSNRSLTHLCLSNNSLGNEGV
NLL---------------------------------------------------------
-------------CRSMRLPHCSLQRLMLNQCHLDTAGCGFLALALMGNSWLTHLSLSMN
PVEDNGVKLLCEVMREPSCHLQDLELVKCHLTAACCESLSCVISRSRHLKSMDLTDNALG
DVGVAALCEGLKQKNSVLTRLGLKACGLTSDCCEALSLALSCNRHLTCLNLVQNNFSPKG
MMKLCSAFACPTSNLQIIGLWKWQYPVQIRKLLEEVQLLKPQIVIDGSWHSFDEDDRYWW
KN----------------------------------------------------------
------------------------------------------------------------
------------------------------------------------------------
-----------------

#NLRP5_mouse "gi|7106379|ref|NP_035990.1| NACHT, leucine rich repeat and PYD
containing 5 isoform a [Mus musculus]"
------------------------------------------------------------
------------------------------------------------------------
--------------MGPPEKESKAILKARGLEEEQKSERKMTSPENDSKSIQKDQGPEQE
QTSEST-------MGPPEKDSKAILKARGLEEEQKSESTMSPSENVSRAILKDSGSEEVE
QASERKMTSPENDSKSIQKDQGPEQEQTSETLQSKEEDEVTEADKD--------------
----------------------------------------------NGGDLQDYKAHVIA
KFDTSVD-----LHYDS-------PEMKLLSDAFKPY-QKTFQPH-TIILHGRPGVGKSA
LARSIVLGWAQGKLFQKMS-FVIFFSVREIK-WTEKSSLAQLIAKECPDSWDLVTKIMSQ
PERLLFVIDGLDDMDSVLQH----DDMTLSRDWKDEQPIYILMYSLLRKALLPQSFLIIT
TRNTGLEKLKSMVVSPLYILVEGLSASRRSQLVLENISNES---------DRIQVFHSLI
ENHQLFDQCQAPSVCSLVCEALQLQKKLGKRCTLPCQTLTGLYATLVFHQLTLKRPSQSA
LSQEEQITLVGLCMMAAEGVWT-MRSVFYDDDLKNYSLKESEILALFHMNILLQVGHNSE
QCYVFSHLSLQDFFAALYYVLEGLE-EWNQHFCF-------IENQRSIMEVKRT-DDTRL
LGMKRFLFGLMNKDILKTLEVLFEYPVIPTVEQKLQHWVS-LIAQQ--------------
-----------VNGTSPMDTLDAFYCLFESQDEEFVGGALKRFQ----------------
-EVWLLINQKMDLKVSS-YCLKHCQNLKAIRVDIRDLLSVDNTLELCPVVTVQET-----
----QCKPLLMEWWGNFCSVLGSLRNLKELDLGDSILSQRAMKILCLELRNQ------SC
RIQKLTFKSAEVVSGLKHLWKLLFSNQNLKYLNLGNTPMKD-DDMKLACEALKHPKCSVE
TLR---------------------LDSCELTIIGYEMISTLLISTTRLKCLSLAKNRVGV
KSMISLGNALSSSMCLLQKLILDNCGLTPASCHLLVSALFSNQNLTHLCLSNNSLGTEGV
QQL---------------------------------------------------------
-------------CQFLRNPECALQRLILNHCNIVDDAYGFLAMRLANNTKLTHLSLTMN
PVGDGAMKLLCEALKEPTCYLQELELVDCQLTQNCCEDLACMITTTKHLKSLDLGNNALG
DKGVITLCEGLKQSSSSLRRLGLGACKLTSNCCEALSLAISCNPHLNSLNLVKNDFSTSG
MLKLCSAFQCPVSNLGIIGLWKQEYYARVRRQLEEVEFVKPHVVIDGDWYASDEDDRNWW
KN----------------------------------------------------------
------------------------------------------------------------
------------------------------------------------------------
-----------------

#NLRP5_rat "gi|109458087|ref|XP_218237.4| PREDICTED: similar to NACHT-, LRR- and
PYD-containing protein 5 (Maternal antigen that embryos require) (Mater protein)
(Ooplasm-specific protein 1) (OP1) [Rattus norvegicus]"
------------------------------------------------------------
------------------------------------------------------------
------------------------------------------------------------
------------------------------------------------------------
----------------------MSVSLKIRFCSHFIFLLSSYLTSY--------------
----------------------------------------------RGGDLQDYKAHVIA
KFDTSVD-----LHCDG-------PEMKVLSDAFKPH-QKTFRPH-TIILHGRPGVGKSA
LARSIILGWAQGKLYQDMS-YVFLFSVREMK-WTEKSSLAQLMANEWPHSQAPVTKIMSQ
PERLLFVIDGLDDMDSALQH----DDMTLSRDWKDKQPIYILVYSLLRKALLSQSFLIIT
TRNTGLENLRSMVVSPLYILVEGLSASRRSQLVLENIPDDH---------QKIQVFHSVI
```

ENHQLFDQSQAPSFCSLVCEALQLQEKLGKRCTLPCQTLTDLYATLVFHQLTSRGPSQRA
LSKEEQITLVGLCRMALEGVWT-MRSVFYDDDLKSYSLKQSEISALFHVNILLQVGHSSE
QCYVFFDLSLQDFFAALYYVLEGLG-EWNQYFSY-------ITNQRNIMEVKRS-SDSHL
LEMKRFLFGLMNKDTLKTVEVLFRCPVTPPIKQKLQHCLS-LIGQQ--------------
-----------VNGSSPMDTLDAFYYLFESQDEEFVHMALKSFQ----------------
-EVWLLINQKMDLMVSS-YCLQQCQNLKAIRVDISDLFSVDNTPKLCP-VALQKT-----
----QCKPLIIEWWENFCTVLSTHPNLKTLDLGHSILNEWSMKILCLKLRNS------SC
SIQNLTFKNSEVVSGLQYLWMLLVSNRNLKYLNLGNTPMKD-DDIKLACEALKHPSCSLE
TLR---------------------LDSCELTLTGYELISKLLLSASSLKCLSLARNKVGV
KSMTSLGEALSSSTCTLQKLILNECFVSPFPTNAVLPALKILS--KHNLSHLCQVLSLSI
SVL---
-------------CSILGNPVCLSRSLLLNHCNIIKDAYGFLALILANNRKLTHLSLTMN
PVGNSAMKLLCEALKEPTCYLQDLELVDCQLTENCCEDLACMITTTKHLQSLDLGNNALG
DKGVITLCKGLKQSSSSLRRLGLAACGLTSKCCEVLSSALSCNPYLNSLNLVRNDFNTSG
MLKLCSAFQNPASNLWIIGLWKQQFYAQVRRQLEEVQFIKPHVVIDGDWYSIDEDDRNWW
KN--

#NLRP5_bovine "gi|56118314|ref|NP_001007815.1| NACHT, LRR and PYD containing protein 5 [Bos taurus]"

--------------------MREAKIAPLSNYGLQWCFEQLGKEEFQTFKALLKEHASES
AACSFP-------LVQVDRADAESLASLLHEHCRASLAWKTSTDIFEKMSLSALSEMARD
EMKKYLLAEISEDSAPTKTDQGPSMKEVPGPRED--------------------------
---PQDSRDYRIHVMT
TFSTRLD-----TPQRFEEFASECPDAHALSGAFNPDPSGGFRPL-TVVLHGPPGVGKSS
LARRLLLFWAQGDLYKGLFSYVFLLRARDLQ-GSRETSFAELISKEWPDAPVPVEKVLSQ
PERLLIVVDGLEELELTFRD----QDSSLLADWAERQPAPVLAHSLLKKVLLPECALLLT
VQDAGLQRLQALLRSPRYLWVGGLSVENRMQLLLGGGKHCR---------RKTCAWHAGA
DHQEVLDKCQVPVVCALVREALELQGEPGKGLPVPGHTLTGLYATFVFQRLAPKDAGWRA
LSGEERGALKGLCRLAADGVWN-AKFVFDGDDLGVHGLQGPELSALQQASILLPDGH-CG
RGHAFSHLSLQEFFAALFYVLRGVEGDGEGYPLF-------PQSTKSLTELRHIDLNVQL
VQMKRFLFGLVSKEVMRALETLLGCPVRPVAKQQLLHWIC-LVGQH--------------
-----------PAAAASPDLLEAFYCLFEAQDDEFVRLALNGFQ----------------
-EVWLQLNRPMDLTVSS-FCLRRCQHLRKVRLDVRGTP-KDEFAEAWS-GAPQGL-----
----KIK-TLDEHWEDLCSVLSTHPNLRQLDLSGSVLSKEAMKTLCVKLRQP------AC
KIQNLIFKGARVTPGLRHLWMTLIINRNITRLDLTGCRLRE-EDVQTACEALRHPQCALE
SLR---------------------LDRCGLTPASCREISQVLATSGSLKSLSLTGNKVAD
QGVKSLCDALKVTPCTLQKLILGSCGLTAATCQDLASALIENQGLTHLSLSGDELGSKGM
SLL---
-------------CRAVKLSSCGLQKLALNACSLDVAGCGFLAFALMGNRHLTHLSLSMN
PLEDPGMNLLCEVMMEPSCPLRDLDLVNCRLTASCCKSLSNVITRSPRLRSLDLAANALG
DEGIAALCEGLKQKN-TLTRLGLEACGLTSEGCKALSAALTCSRHLASLNLMRNDLGPRG
MTTLCSAFMHPTSNLQTIGLWKEQYPARVRRLLEQVQRLKPHVVISDAWYTEEEEDGPCW
RI--

#NLRP5_dog "gi|73947399|ref|XP_533576.2| PREDICTED: similar to NACHT, LRR and PYD containing protein 5 [Canis familiaris]"

--------------------MREAKLPCFSNYGLQWYFKQLSKEEFQTFKELLMEKASEL
AVCSFP-------WVEVSNANVEHLASLLHEHYKESLAWKISIHIFEEMNLSTLSEKARK
EMKKYSLTEIPE----NSTPKKNDQEPNMKEGPGQRKD----------------------
---VGNTWNYKDYVMT
KFATKLD-----EYHGSENFASDYPEMQTLLGAFNPD-QRGFQPR-TVVLHGKSGIGKSD

LARRVLLSWAQGKLYQGMFSYVFFLGAREMH-CMKEGSFAELISREWPDYQVPMMDIMSQ
PERLLFVVDGFDDLDFAFKD----NDPNLCEDWTDKQPTSVLMHSLLKKVLLPESSLMIT
VRDTGIEKLKSMLTSPRYLLVRGISVEKRIQLLCQHTKNEH---------LKTQVLHSVV
DNHLLFEECQAPILGSLICEALNLQEASGKSLPPTCQTLTGLYATFVFHQLTPRDASRRC
LNQEERAILKNLCLMAMRGVWN-RKFVFYRDDLSVHRLRKSELSALFQTNILLQDGS-HE
RCYTFLHVSLQDFCAALYYILEGLETEWDPYPVF-------MENMRSLVELKQISSNAHL
FQMKRFLFGLMSKEVMGALEVLLGCPVPLVMKQGLLCWIS-LLGQQ--------------
-----------ASTATPPDSLDAFHCLFETQDDEFVLLALNSFQ----------------
-EVWLPINRRMDLMVSS-FCLQRCQYLQKIRMDVCESFLKDEFTEAHP-LAPQGM-----
----PIKTLADEWWESLCLVLSTHPSLQQLDLSGSTLNEWAMKTLCIKLRQP------TC
KIQNLIFKGAQVTLGLRHLWMTLITNRNIKCLNLESTHLKD-EDLMMVCEALRHPNCLLE
SLR---------------------LDHCGLTPACCLVISQILVTSISLKSLSLVGNKVTN
QGIKPLCDALTASQCTLQKMILGKCGLKAPDCQDLASALISNQSLTHLCLSSNSLGSEGM
NLL---
-------------GRAIKFPNCGLQRLILNECNLDVAGCGFLAFALMGNRHLTHLSLSMN
PLEDDGMNLLCEVIMEPSCHLQDLELVKCHLTAGCCKKLSHVIVRNKHLKSLDLAVNALG
DDGVTALCEGLKHRKASLKRLGLEACGLTSGCCEVLASALLCSQRLTSLNLMRNDFSPMG
MTKLCPAFAQPTSNLQIIGLWKWEYPTQIRKLLKEVQLVKPQIVIGDDWYSFDEDDRYWW
KS--

#NLRP8_dog "gi|73947401|ref|XP_853947.1| PREDICTED: similar to NACHT, leucine rich repeat and PYD containing 8 [Canis familiaris]"

--------------------MSSASSTSPFENGVMLYMVYLSKEELQTFKQLLVDERPRP
GSVQIT-------WDQVKTARWGEVVHLLMEYFPGRLAWDVTHDIFAKMNQMELCLRVQM
ELNDILPSLELEDSNPREMPVSLEEGE---------------------------------
---SDKIQEYKLYMID
EYSMIRSKTTWPGNHMDFFYQDIERHERFLPCIFLPKRPQGRQPK-TVVLQGVSGVGKSS
LAGKVMLEWAKNKFYSHKFWYAFYFHCREVA-QVDEQSLSELIAHKWPRAQALMSKIMSK
PDQLLLLFDGFEEVTLTLTD----RPTDLSEDWSQKLPGSVLLTSLLSKIMLPEATLLIT
LRFTSWRKLKPFLKHPSFITLTGFSMVEKVKYFRTYFRNKR---------EADEALSFVM
GNTILFSMCQVPVICWMVCSCLRQQMEKGVDLVQAYPNATAVFVQYLSSLFPTKAGS--L
PSNTHQEQLRGLCYLAAEGMWN-MRWVFDMKDFEQAKLDETAVATFLHVNIFRRVAG-DK
DYYAFADLSFQEFFAALLYVLCFPQRLRNFWVLD--------RVQIICLIAYPGRKKNHL
AQMGLFLFGLLNEKCSLAAEKSFRCKLSLGNKKKLLKVAALPYEC---------------
-----------GPPTLHHGIPQLFYCLHEIQEEAFVSQILSDCQ----------------
-KAALIISRNKDMQASA-FCLKHCRHLRNLELTITLTITQLYALNPDPLQSTEPG-----
-----SSNQCFLWWQDFCSVVRTHENLEVLAVTNTVMEADSVKVLSAALRHP------GC
KLQKLIFRRVNTSMLNEDLLQVLMENWYLRCLEIQGTEVRC-EAMEFLCTALKYPQCYLQ
CLR---------------------LEDCAVTPKSWVELARDLGSNRHLKTLMLRSSCLET
FG------AYYLSLAQLERLLLENCGLTVLSCKSLMSSLMSNKMLTHLSLAENALKDEGA
KQL---
-------------CSALQHPMCPLQRLVLRNCALTSDCCQDMASALGKNKNLRSLDLGFN
SLKDDGVTLLFEALKKSESGLQILELERCLFTSLCCQAMASMLLHNQSLRYLDVSKNDIG
LRGILLLREAFQQHKWKKKVVLETKQNYAPDMVMRLEGPAVKNESLRIIQDWSADCWDEA
RWME--

#NLRP1a_mouse "gi|51921281|ref|NP_001004142.1| NLR family, pyrin domain containing 1A [Mus musculus]"

------MEE-------SQSKQESSTKVAQHEGQEDVDPTFKTKKLMEVELMKHRVQLERN

LKLR--
------------------TFPGARTKQVKEALYPLLTWSSKSKNLFQNFTKLLLFKKLCQ
RG---SENLVRESWYPCVPEE---EAHMIDIQDLFGPNLGTQKKPQLVIIEGAAGIGKST
LARLVKRAWKEGKLYRNDFHHVFFFSCRELA-QYEQLSLAELIVQGQEVPTAPIRQILSH
PEKLLFILDGIDEPAWVLAD----QNPELCLHWSQTQPVHTLLGSLLGKSILPGASFLLT
TRTTALQKFIPSLEQPCQVEVLGFTLFERKNYFYKYFGKKK---------GGVTTFTLVK
SNSALLTLCEVPWVCWLVCTCLKKQMEQGGELSLTS-QTTTALCLKYLSLTIPG------
QHMRT--QLRDLCSLAAEGVCQ-RRTLFSESDLCKQGLDEHAIASFLKIGVLQKQASSLS
--YSFAHLCLQEFFAAMSYILDDSEERH-ADMKND--------RIVETLVERYGRQNLFE
APTVRFLFGLLSKEELKKIEKLFSCSLHGK--TKLKLLWHILGKSQPHQP----------
------------------PCLGLLHCLYENQDMELLTHVMHDLQGTIVPGPDDLAHTVLQ
TNVKHLVIQTDMDLMVVTFCIKFCCHVRSLQLNRKVQQGHKFTAPGMVLYRWTPITDA--
------------SWKIFFSNLKLARNLEELDLSGNPLSYYAVHSLCTTLRKR------GC
QLKTLCTLPEDMNKAEVKNTEAKGSLGEKAPGPRNDIFPFPPA----HCPYNQTSTSEHL
PCR---------------------LVECGLTSTYCSLLASVLSARSSLTELDLQLNDLGD
GGVKMLCEGLRNPACNLSILWLDQASLSDQVIAELRTLEAKNPKLLISSTWKPHVMVPTM
NMDKEEVGDSQALLKQQRQQSGMVGWLLKEDNVKKIKQNATN----TYAHTHTHTHAELV
PLKTFHPCSPVSPGDKHMEPLGTEDEFWGPTGPVTTEVVDRERNLYRVQLPMAGSYHCPS
TGLHFVVTRAVTIEIEFCAWSQYLDKTPLQQSHMVVGPLFDIKAEQGAVTAVYLPHFVAL
QEGIVDSSLFHVAHFQEHGMVLETPARVEQHYAVLENPSFSPMGILLRMIPAVGHFIPIT
STTLIYYHLYLEDVTFHLYLVPNDCSIRKAIDDEEMKFQFVRINKPPPVDALYLGSRYIV
SSSKL--VEIIPKELELCYRSPGESQLFSEIDIGHMDSEIKLQIKDKRHMNLKWEALLKP
G----KISLRPETLRVEERDTKGAFLCSFPVSSLSLDAPSLLHFMDQHREQLVARVTSVD
PLLDKLHGLVLSEDSYEVVRSETTNQDKMRKLFSLSRSWSWDCKDQFYQALKETHPHLVM
DILEKLGGVSVKS----

#NLRP1b_mouse "gi|110665740|ref|NP_001035786.1| NLR family, pyrin domain containing 1B [Mus musculus]"

------MEQ-------SQPKKKSRTKVAQHEGQLNLNPTFKTRKRKEVELMKRRPKPEGH
LKLG--
------------------TIPKVHIKQKGETLDP--TWSRKRKNLVQKLTNLLLFEKLCS
RG---SENLIRKSWYSCEEEE---RGHMIEIQDLFGPNRGTHKKPQLVIIEGAAGIGKST
LARQVKRAWKEGQLYRNHFQHVFFLSCRELA-QYEQLSLAELIAQGQEVPTVPIRQILSH
PKELLFILDGIDEPAWVLAD----QNPELCLHWSQRQPVHTLLGSLLGKSILPGASFLLT
ARTTALQKIIPYVGQPRRVEVLGFSKFEREVYFRKYFVKES---------DAIAAFRLVV
SNPVLLTLCEVPWVCWLVCTCLKKQMEQGGELSLTS-QTTTALCLKYLSLTIPG------
QHMRT--QLRALCSLAAEGICQ-RRTLFSESDLCKQGLAEDAIATFLKIGILQKQASSLS
--YSFAHLCLQEFFAVMSYILEDSDERC-DGMEFK--------RTVETLIEVYGRHTLCE
EPTVHFLFGLVNEQGMREMKKIFDCKLPLG--TELKMLKSTLG--NPTYQ----------
------------------HHLGLLHCLYESQEEVLLTYVLCNLH-LTGPDKNYMEATVSQ
TNVKHLVIQTDMELMVVTFCIQFCCHVRSLRVNMKGQQGHKLTVASMVLYRWTPITNA--
------------SWKILFYNLKFNSNLEGLDLSGNPLSYSAVQYLCDAMIYP------GC
QLKTLW--
------------------------LVECGLTPTYCSLLASVLSACSSLRELDLQLNDLCD
DGVRMLCEGLRNRACNLRILRLDLYSLSAQVITELRTLEENNLKLHISSIWMPQMMVPTE
NMDEEDILTSFKQQRQQS--
-------------GANPMEILGTEEDFWGPIGPVATEVVYRERNLYRVQLPMAGSYHCPS
TRLHFVVTRAVTIEIEFCAWSQFLDKTPLQQSHMVVGPLFDIKAEQGAVTAVYLPHFVSL
KDTKASTFDFKVAHFQEHGMVLETPDRVKPGYTVLKNPSFSPMGVVLRIIPAARHFIPIT
SITLIYYRVNQEEVTLHLYLVPNDCTIQKAIDDEEMKFQFVRINKPPPVDNLFIGSRYIV
SGSEN--LEITPKELELCYRSSKEFQLFSEIYVGNMGSEIKLQIKNKKHMKLIWEALLKP
G----DLRP-----------------ALPRIAQALKDAPSLLHFMDQHREQLVARVTSVD
PLLDKLHGLVLNEESYEAVRAENTNQDKMRKLFNLSRSWSRACKDLFYQALKETHPHLVM
DLLEKSGGVSLGS----

#NLRP1c_mouse "gi|85986637|ref|NP_001034323.1| NLR family, pyrin domain containing 1C [Mus musculus]"

------------------------MYMKQKKENLYSTQPSKGENLSQNFTNLLLSQKLCP
QG---SKTFARKSWHQCVPEE---RGHMIEIQDLFGQNLNKQEKAQLVVIVGAAGIGKST
LARQVRNAWKEGQLYRDHFQHVFFFSCTELS-KSKQLSLAEFIAQGQEVPTAPIRQILSH
PEELLFILDGIDEPAWVLKD----QDPELCLHWSQMQPVHTLLGSLLGKSILPGASFLLT
ARTTALKKFISNFKQPWQVEVLGFTMLERKNYFYKYFEKEI---------EAIAAFRLIK
SNSVLLTLCEVPWVCCLVCNCLKKQMDQGRVISLTS-QTTTALCLKYLSLTIPG------
KHRRT--QLRALCSLAAEGIWK-RRTLFSESDLCKQGLEEDAIATFLKIGVLKKQASSLS
--YSFAHLCLQEFFAAMSYILEDSEGRH-GDMKIN--------RTVRILVKRYGRQNLFE
APTVRFLFGLLSKEGFKEMQKVFSCKLSGK--SKLMIR-HILQKLQPHQP----------
------------------SCLGLLHCLYEKQEMELLTLMMHDLRGTIMHGLINIAHTVLQ
RNLKNLVIQTDMDLMVVTFFIRLCSHVRSLQLNRKGQQGYKLMAPGMVLYRWTPITDS--
------------SWKIFFFNLKFTTNLEELDLSGNPLSYSAACSLCKALRHP------GC
QLKTLW--
------------------------LVECGLTSTYCSLLASVLSACSSLTELNLRLNYLGN
RGVKLLCEGLRNPACNLSTLWLDVASLREEVITELRTLEAKNLKLLISRACKSHVIVPTK
NMDKEEVGDSLDLLKQQRQQS---------------------------------------
-------------GDKHMEPLGTDYDFWGSTGPVATEVVDRERNLYRVQLPMAGSYHSPS
TGLHFVVTRAVTIEIEFCAWSQYLDKTPLKHSHMVAGPLFDIKAEQGTVSAVYLSRFVSL
QEGIVDSSLFHVAHFQEHGIIPRNTSKGGTALCSTGKPKLLPNGNSTENDSGCRTLHPHH
FHHTDLLSPLSRGCHLSPLLGPQ-------------------------------------

#NLRP1_rat "gi|109488421|ref|XP_340836.3| PREDICTED: similar to NACHT, leucine rich repeat and PYD containing 1 [Rattus norvegicus]"

------MEE-------SQSKQESNTRVAQHGSQQDVDPTFQTKRALEKERSKPRPRPLPR
GTIP--
------------------EIYEKRKETIS------HTQSMEQKYLFQNFTKLLLLQKCCP
GG---SEKLVRESWHPCVPEE---GGHMIEIQDLFDPNLDTEKKPQLVIIEGAAGIGKST
LARQVKRAWDEGQLYRDRFQHVFFFSCRELA-QCKQLSLAELIAQGQEVPTAPTRQILSR
PEKLLFILDGIDEPAWVLED----QNPELCVHWSQAQPVHTLLGSLLGKSILPEASLMLT
ARTTALQKLVPSLGQPHRVEVLGFSEFERKDYFYKYFAKER---------NTIIDFNLIG
SIPVLLTLCEVPWVCWLLCTCLEKQMQQGEVLSLTS-QTTTALCLKYLSLTIPG------
QHLST--QLRTLCSLAAEGICQ-RRTLFSKSDLCKQGLAEDAIATFLKIGVLQRQPSSLS
--YSFAHLCLQEFFAAMSYILEDSEEAH-GDMGND--------RTVETLVERYGRQNLFE
APTVRFLLGLLNTREMREMENIFACKFPWE--TKLKLLQSIIG-EPFCQP----------
------------------CHLGLFHCLYENQEEELLTETMLCFP-LTASGPNHMEATVFQ
TNVKRLVIQTDMELMVVTFCITFS-HVRSLRLKGKGQQEYKLTAPAMVLYRWTPISEA--
------------SWKVLFSNLKCTRNLEELDLSGNPLSYSAVRSLCTALRQP------GC
RLKTLW--
------------------------LVDCGLTSRCCSFLASMLSAHSRLAELDLRLNDLGD
NGVRQLCEGLRNPACNLSILRLDQASLSEQVITELRALETKNPKLFISSTWMSHMTMPTE
NTDGEESLTSSKQQQQQS--
-------------GDKHMEPLGTDDDFWGPSGPVSTEVVDRERNLYRVRLPMAGSYHCPS
TGLHFVVTRAVTIEIGFCAWSQFLHETPLQHSHMVAGPLFDIKAEHGAVTAVCLPHFVSL
QEGKVDSSLFHVAHFQDHGMVLETPARVEPHFAVLENPSFSPMGVLLRMIPAVGHFIPIT
SITLIYYRLYLEDITFHLYLVPNDCTIRKAIDEEELKFQFVRINKPPPVDALYVGSRYIV
SSSKE--VEILPKELELCYRSPRESQLFSEIYVGNIGSGINLQLTDKKYMNLIWEALLKP
GDLRP---------------------ALPRMASAPKDAPALLHFVDQHREQLVARVTSVD
PLLDKLHGLVLSEEDYETVRAEATNQDKMRKLFRGSRSWSWDCKDHFYQALKETHPHLIM
DLLEKSGGVSVRL----

#NLRP1_bovine "ENSBTAT00000027232 peptide:ENSBTAP00000027232
pep:NOVEL_protein_coding"
--MAGEARSLEDLEEKKKQKLKELRLWLPDEEPRLQSPEVVHAQPEKPRSRKTMTILSVM
KEVQHECMAIHTKGPHVNEQGACTKTLEETDLNSALYSSFSHSPSAPNLESPSWPTSTKV
LTFCKPKQPV--LNPLKWSSSPLIFASGYRKEDLSPSIYQG--FPSSPYHESPSQELPND
PTSTAVLRA-------CEIPRHSCPEPREKEGPGTVWPLVETSGNSCTEILLLENINSQN
GRDGIQPPLSLMPLL---
-------PPSTHLMFCFPCHRSRQRYQDQRRETPCSTWSWKNEDLHPKFMQLLLLHTPYH
KD---YESLNRESWDHGVVEG---QGHLIEVRDLFGSDLGSQEGPHTVILHGVAGIGKST
LARHIRRAWEEGQLYRNHFQHVFYFNCRELA-TSKLTSLEELITKDQSTTVAPAGQIQSQ
PEQLLFILDGLDELECVSEE----KRAELCLHRSQQQSVQTLLGSLVEKTIFPEASLLIT
ARTAALQKFIPSLKQPCWVEVLGFSESSRKDYFYEYFTEES---------QATRAFSVVE
SNQALLTMCLTPLVSWLACTCLKQQMEAGEELSLKS-QTMTALYLRYLAQAIQA------
QPLGT--QLRGFCTLAAEGICQ-GKTLFSPGDLKKHGLDGVISATPLKMDVLQKHPTSLN
--YSFIHLCFQEFFAAVSFALGDIRDKS-DHPNST--------GSMEKLVEVYGINNLFG
APTVHFLSGLLSEQGAREMENIFQCKLSWE--RKRESLLRWAELELRR------------
----------------EHYSLQLFHCLYEIQDEEFLTQAMAHFQ----------------
--GMRVCIRTSMELLVFTFCIRFCSHVWRLQLNEGRQHPGACRPTDTVLLSWVPATEA--
------------CWQVLCSVLQVTGNLKELDLSRNILSRSAVQSLDEALRCP------GC
QLETLRLASCSLTAESCKDIASGLSTSQTLMQLELSFNTLLDAGAEHLCQGLRQPTCKLQ
RLL---------------------LAGCGLTSGCCQDLASMLRVSPSLMELNLLQNDLDD
LGVRLLCEGLRHPSCQLICLWLDQTQLSEEVTKMLRALERQKPQLLVSGIWKPSLRILHE
GPGGAEMS-DTSSLKRQRQASARQKNLKLAAALS----------------APEGSSPQAA
QVRLFCPSSPAPPEDEHMESLGTEDDLWGPTGPVATEAVDEERSLYRVHLPMAGFYHWPN
TGLRFEVRGPVTVEIEFCAWDQFLNRNFPQRSWLAAGPLFDIKAEPGAVAAVFLPHFVAF
RGNDVDISQFQVAHFKEEGMLLEKPARVTPCYAVLENPSFSPMGVLLRMVQTALRFIPIT
STVLLYHYLQPEEVTFHLYLIPSDCSIQKAIDNEEKMFQFVRIHKPPPLTPLYMGSRYIV
SGSEK--LEILPEELELCYRSPRESQLFSEFYVRHLRSGIRLQIRSKKDGTVVWEALVKP
GDLRRAVTPVS---------------PGLRA-----------------------------

#NLRP1_dog "ENSCAFT00000024581 peptide:ENSCAFP00000022816
pep:NOVEL_protein_coding"
MACRVQWQLAWYLEMMGKEELKEFQLRLLEQQFWGDPPHALRAQLGKARGLEVASRLVAQ
YGEQQAWVLALRTWEEMGLSRLCAQSRAEAGLIPLSPG----SPMAPSMQSPNSPISTEV
LRFIQDPRTR--NSGQPLPPLLPNTSGHMWNEDPSSCKYQEGLPDSPEFQGSPHQESPSA
PMPTAVLGG-------WEAPPQPSPVPGKQEAPKAGWPVAGTSGNHNPGERNRSQTESPQ
GRS---
-------VPYLYVPSFPPLAGITERYQHQRRKKPYPTWSWEDKDLHQIFIQLLLLHQPYP
RG---HESLTKGRWHHGAEK----QGHLIEVEDLFGPGLGAQEEPQTVILHGVAGIGKST
LARQIRTAWKEGQLYKDRFRHVFYFNCRELA-RSETMSLTELITKAQAGPVLPAEQILSQ
PGRLLFILDDLDEPRWVLGE----QESELHSSWSKQRPVHALLGSLLGKTVLPEVSLLVT
VRTTALRRLVPSVGQLCWVEVLGFSESGRREYFYKYFKNKS---------QATQAFSLVE
LNPTLLTMCLVPLVSWLLCTCLKKQMQRGEELSLGSSQTTTALWLYYLSQALPA------
QLLGA--QLRGFCSLAAEGFWQ-GTNLLSRRDLRKHGFDEATISTFLKTGILRKHPSPLN
--YHLAHRCLQEFFAAVSCVLGGEDGGS-PHPDSI--------RGAEKLLEVYEGHDLFG
APTTHFLFGLLSEQGEREMEAVFQCKLPRE--RKRD-LLRWAEAGLWPRLS---------
--------------APQPCSLHLLHCLYEIQDEDLLTRAMAHFH----------------
--GRSMCVQTGVEFLVCTFCFKFCSHVKRLQLNESRWDGQAWRPSSVALFSWVPVTDA--
------------CWQALFSTLRASGSLKELDLSGNHLSHPAVQSLCKVLKFP------CC
HLETVRLAGCGLTAEGCSDLASALSASPALAELELSFNLLLDAGAQHLSRGLRQPACRLR
RLL---------------------LAGCSLTSRCCGALASALSTSPLLTELDLQQNELGD
VGVRLLCGGLGHPTCQLTLLWLDQTQLSEEMTQMLRALQEEKPQLFVCSKWKPRATVPTE
GLSRGETS-GTSSLKQQRQGSGRKSNAVAQARQPSLDRESGA--DSCSAPFPERSCPQVG
QVQLGRPPSPAAPWDLNTEPLRIGDGFWGPLGPVAPEWLAEDGSLYRVHLPVAGSYHWPD
TGLRFEVRGPATIDIEFCVWDQHLPRAGLWHSWMVAGPLFDIKAEPGAVATVCLPHFVDL
QGSSVDTSLFYAAHLKDEGMLVETPARVEADHVVLENPSFSPIGVLLRTIHAALRFLPVT
CVVLLYHHLRPGEVAFHLYLIPSDYSIRKAIDNEEKKFQFVQIHKPPPLASLYMGSRYTV
SGSEK--LEIIPKELELCYRSPREPQLFSEFYVSHLGSGIRLQVRDKKDDTVVWEALVKP
GDLRPVASLAP---------------PAPAARPFPTDAPAWLHFVDRHREQLVARVTSVD

PVLDMLHGQVLSEEQYERVRAEATTPSQMRKLFSFSRSWDRACKDQVYQALKEAHPHLIM
ELWEKWGGNSGKQAPPG

#NLRP1_human "gi|14719829|ref|NP_127497.1| NLR family, pyrin domain containing 1 isoform 1 [Homo sapiens]"
MAGGAWGRLACYLEFLKKEELKEFQLLLANKAHSRSSSGETPAQPEKTSGMEVASYLVAQ
YGEQRAWDLALHTWEQMGLRSLCAQAQEGAGHSPSFPYS----PSEPHLGSPSQPTSTAV
LMPWIHELPAGCTQGSERRVLRQLPDTSGRRWREISASLLYQALPSSPDHESPSQESPNA
PTSTAVLGS-------WGSPPQPSLAPREQEAPGTQWPLDETSGIYYTEIREREREKSEK
GRPP--------WAA---
-------VVGTPPQAHTSLQPHHHPWEPSVRESLCSTWPWKNEDFNQKFTQLLLLQRPHP
RS---QDPLVKRSWPDYVEEN---RGHLIEIRDLFGPGLDTQE-PRIVILQGAAGIGKST
LARQVKEAWGRGQLYGDRFQHVFYFSCRELA-QSKVVSLAELIGKDGTATPAPIRQILSR
PERLLFILDGVDEPGWVLQE----PSSELCLHWSQPQPADALLGSLLGKTILPEASFLIT
ARTTALQNLIPSLEQARWVEVLGFSESSRKEYFYRYFTDER---------QAIRAFRLVK
SNKELWALCLVPWVSWLACTCLMQQMKRKEKLTLTS-KTTTTLCLHYLAQALQA------
QPLGP--QLRDLCSLAAEGIWQ-KKTLFSPDDLRKHGLDGAIISTFLKMGILQEHPIPLS
--YSFIHLCFQEFFAAMSYVLEDEKGRG-KHSNCI--------IDLEKTLEAYGIHGLFG
ASTTRFLLGLLSDEGEREMENIFHCRLSQG--RNLMQWVPSLQLLLQP------------
------------------HSLESLHCLYETRNKTFLTQVMAHFE----------------
--EMGMCVETDMELLVCTFCIKFSRHVKKLQLIEGRQHRSTWSPTMVVLFRWVPVTDA--
------------YWQILFSVLKVTRNLKELDLSGNSLSHSAVKSLCKTLRRP------RC
LLETLRLAGCGLTAEDCKDLAFGLRANQTLTELDLSFNVLTDAGAKHLCQRLRQPSCKLQ
RLQ---------------------LVSCGLTSDCCQDLASVLSASPSLKELDLQQNNLDD
VGVRLLCEGLRHPACKLIRLGLDQTTLSDEMRQELRALEQEKPQLLIFSRRKPSVMTPTE
GLDTGEMSNSTSSLKRQRLGSERAASHVAQANLKLLDVSKIFPIAEIAEE----SSPEVV
PVELLCVPSPASQGDLHTKPLGTDDDFWGPTGPVATEVVDKEKNLYRVHFPVAGSYRWPN
TGLCFVMREAVTVEIEFCVWDQFLGEINPQHSWMVAGPLLDIKAEPGAVEAVHLPHFVAL
QGGHVDTSLFQMAHFKEEGMLLEKPARVELHHIVLENPSFSPLGVLLKMIHNALRFIPVT
SVVLLYHRVHPEEVTFHLYLIPSDCSIRKAIDDLEMKFQFVRIHKPPPLTPLYMGCRYTV
SGSGSGMLEILPKELELCYRSPGEDQLFSEFYVGHLGSGIRLQVKDKKDETLVWEALVKP
GDLMPATTLIP---------------PARIAVPSPLDAPQLLHFVDQYREQLIARVTSVE
VVLDKLHGQVLSQEQYERVLAENTRPSQMRKLFSLSQSWDRKCKDGLYQALKETHPHLIM
ELWEKGSKKGLLPLSS-

#NLRP1_chimpanzee "gi|114666001|ref|XP_001167230.1| PREDICTED: death effector filament-forming Ced-4-like apoptosis protein isoform 8 [Pan troglodytes]"
MAGGAWGRLACYLEFLKKEELKEFQLLLANKAHSRSSSGETPAQPEKTSGMEVASYLVAQ
YGEQRAWDLALHTWEQMGLRSLCTQAQEGAGHSPSFPYS----PSEPHLGSPSQPTSTAV
LMPWIHELPAGCTQGSERRVLRQLPDTSGRRWREISASLLYQALPSSPDHESPSQESPNA
PTSTAVLGS-------WGSPPQPSLAPREQEAPGTQWPLDETSGIYYTEIREREREKSEK
GRPP--------WAA---
-------VVGTPPQAHASLQPHHHPWEPSVRESLCSTWPWKNEDLNQKFTQLLLLQRPHP
RS---QDPLVKRSWPDYVEEN---RGHLIEIRDLFGPGLDTQE-PRVVILQGAAGIGKST
LARQVKEAWGRGQLYGDRFQHVFYFSCRELA-QSKVVSLAELIGKDGTATPAPIRQILSR
PERLLFILDGVDEPGWVLQE----PSSELCLHWSQPQPADALLGSLLGKTILPEASFLIT
ARTTALQNLIPSLEQARWVEVLGFSESSRKEYFYKYFTDER---------QAIRAFRLVK
SNKELWALCLVPWVSWLACTCLMQQMKQKEKLTLTS-KTTTTLCLHYLAQALQA------
QPLGP--QLRDLCSLAAEGIWQ-KKTLFSPDDLRKHGLDGAIISTLLKMGILQEHPIPLS
--YSFIHLCFQEFFAAMSYVLEDKKGRG-KHSNCI--------IDLEKMLEAYGIHGLFG
ASTTRFLLGLLSDEGEREMENIFHCRLSQG--RNLMQWVPSLQLLLQP------------
------------------HSLESLHCLYETRNKTFLTQVMAHFE----------------
--EMGMCVETDMELLVCTFCIKFSHHVKKLQLIEGRQHRSTWSPTMVVLFRWVPVTDA--
------------YWQILFSVLKVTRNLKELDLSGNLLSHSAVKSLCKTLRRP------RC
LLETLRLAGCGLRAEDCKDLAFGLRANQALTELDLSFNVLTDAGAKHLCQRLRQPSCKLQ
RLQ---------------------LVSCGLTSDCCQDLASVLSASPSLKELDLQQNNLDD
VGVRLLCEGLRHPACKLIHLGLDQMTLSDEMRQELRALEQEKPQLLIFSRRQGETQTPTD
GLEVSEPGKNITGLQQPSDGSERAASHVAQANLKLLDVSKIFPIAEIAGKSHEESSPEVV
PVELLCVPSPASQGDLHMKPLGTDDDFWGPTGPVATEVVDKEKNLYRVHFPVAGSYRWPN
TGLCFVVREAVTVEIEFCVWDQFLGEINPQHSWMVAGPLLDIKAEPGAVEAVHLPHFVAL
QGGHVDTSLFQVAHFKEEGMLLEKPARVELHHIVLENPSFSPLGVLLKMIHNALRFIPVT

SVVLLYHRLHPEEVTFHLYLIPSDCSIRKAIDDLEMKFQFVRIHKPPPLTPLYMGCRYTV
SGSGSGMLEILPKELELCYRSPGEDQLFSEFYVGHLGSGIRLQVKDKKDETLVWEALVKP
GRNTSQPWNLR---------------CNRDARRC--------------------------

#NLRP2_human "gi|8923473|ref|NP_060322.1| NLR family, pyrin domain containing 2 [Homo sapiens]"

-----------------------MVSSAQMGFNLQALLEQLSQDELSKFKYLITTFSLAH
ELQ-------KIPHKEVDKADGKQLVEILTTHCDSYWVEMASLQVFEKMHRMDLSERAKD
EVRE---AALKSFNKRKPLS
LGITRKERPPLDVDEMLERFKTEAQAFTETKGNVICLGKEVFKG-----KKPDKDNRCRY
ILKTKFREMWKSWPGDSK-EVQVMAERYKMLIPFSNPRVLPGPFSYTVVLYGPAGLGKTT
LAQKLMLDWAE-DNLIHKFKYAFYLSCRELS-RLGPCSFAELVFRDWPELQDDIPHILAQ
ARKILFVIDGFDELGAAP----GALIEDICGDWEKKKPVPVLLGSLLNRVMLPKAALLVT
TRPRALRDLRILAEEPIYIRVEGFLEEDRRAYFLRHFGDED---------QAMRAFELMR
SNAALFQLGSAPAVCWIVCTTLKLQMEKGEDPVPTCLTRTGLFLRFLCSRFP--------
QGAQLRGALRTLSLLAAQGLWA-QTSVLHREDLERLGVQESDLRLFLDGDILRQDRV-SK
GCYSFIHLSFQQFLTALFYTLEKEEEEDRDGHT------WDIGDVQKLLSGVERLRNPDL
IQAGYYSFGLANEKRAKELEATFGCRMSPDIKQELLRCDISCKG----------------
------------GHSTVTDLQELLGCLYESQEEELVKEVMAQFK----------------
-EISLHLN-AVDVVPSS-FCVKHCRNLQKMSLQVIKENLPENVTASESDAEVERS-----
----QDDQHMLPFWTDLCSIFGSNKDLMGLAINDSFLSASLVRILCEQIASD------TC
HLQRVVFKNISPADAHRNLCLALRGHKTVTYLTLQGNDQDD--MFPALCEVLRHPECNLR
YLG---------------------LVSCSATTQQWADLSLALEVNQSLTCVNLSDNELLD
EGAKLLYTTLRHPKCFLQRLSLENCHLTEANCKDLAAVLVVSRELTHLCLAKNPIGNTGV
KFL---
-------------CEGLRYPECKLQTLVLWNCDITSDGCCDLTKLLQEKSSLLCLDLGLN
HIGVKGMKFLCEALRKPLCNLRCLWLWGCSIPPFSCEDLCSALSCNQSLVTLDLGQNPLG
SSGVKMLFETLTCSSGTLRTLRLKIDDFNDELNKLLEEIEEKNPQLIIDTEKHHPWAERP
SSHDFMI---

#NLRP2_chimpanzee "gi|114679066|ref|XP_001175071.1| PREDICTED: NACHT, leucine rich repeat and PYD containing 2 isoform 2 [Pan troglodytes]"

-----------------------MVSSAQMGFNLQALLEQLSQDELSKFKYLITTFSLAH
ELQ-------KIPHKEVDKADGKQLVEILTSHCDSYWVEMASLQVFEKMHRMDLSERAKD
EVRE---AALKSFNKRKPLS
LGITRKERPPLDVDEMLERLKTEALDK---------------------------DNRCRY
ILKTKFREMWKSWPGDSK-EVQVMAERYKMLIPFSNPRVLPGPFSYTVVLYGPAGLGKTT
LAQKLMLDWAE-DNLIHKFKYAFYLSCRELS-RLGPCSFAELVFRDWPELQDDIPHILAQ
ARKILFVIDGFDELGAAP----GALIEDICGDWEKKKPVPVLLGSLLNRVMLPKAALLVT
TRPRALRDLRILAEEPIYVRVEGFLEEDRRAYFLRHFGDED---------RAMRAFELMR
SNADLFQRGSAPAVCWIVCTTLKLQMEKGEDPVPTCLTRTGLFLRFLCSRFP--------
QGAQLRGALRTLSLLAAQGLWA-QTSVLHREDLERLGVQESDLRLFLDGDILRQDRV-SK
GCYSFIHLSFQQFLTALFYALEKEEEEDRDGHT------SDIGDVQKLLSGVERLRNPDL
IQAGYYYFGLANEKRAKELEATFGCRMSPDIKQELLRCDISCKG----------------
------------GHSTVTDLQELLGCLYESQEEELVKEVMAQFK----------------
-EISLHLN-AVDVVPSS-FCVKHCRNLQKMSLQVIKENLPENVTASESDAEVERS-----
----QDDQHMLPFWTDLCSIFGSNKDLMGLAINDSFLSASLVRILCEQIASD------TC
HLQRVVFKNISPADAHRNLCLALRGHKTVTYLTLQGNDQDD--MFPALCEVLRHPECNLR
YLG---------------------LVSCSATTQQWADLSLALEVNQSLTCVNLSDNELLD
EGAKLLYTTLRHPKCFLQRLSLENCHLTEANCKDLAAVLVVSRELTHLCLAKNPIGNTGV
KFL---

```
-------------CEGLRYPECKLQTLVLWNCDITSDGCCDLAKLLQEKSSLSCLDLGLN
HIGVKGMKFLCEALRKPLCNLRCLWLWGCSIPPFSCEDLCSALSCNQSLVTLDLGQNPLG
SSGVKMLFETLTCSSGTLRTLRLKIDDFNDELNKLLEEIEEKNPQLIIDTEKHHPWEERP
SSHDFMI-----------------------------------------------------
------------------------------------------------------------
------------------------------------------------------------
------------------------------------------------------------
-----------------

#NLRP2_mouse "gi|112821677|ref|NP_808358.2| NACHT, leucine rich repeat and PYD
containing 2 [Mus musculus]"
------------------------------------------------------------
------------------------------------------------------------
-----------------------MEHFDPLGFNLKDVLRKLDKTELNNFKRTLRSCSLPD
TMK-------QINKLTMDLANGAQLAEILTDHCPSAWIKRVTVQILEEINRVDLAELVVK
QIEE-------------------------------------------AVLKVPEEKVSSK
PREPSGTLTFPWNFVQGAKRPEDKQKE--------------------------------E
WKTRYTAKWKQNFWPKCNKEIYVVTESYKTLLALCNP-KIETPFAHAIVLHGPPGSGKTT
MAKQLMLEWSE-SKQAQIFSCAFYISCREVN-NTKPCTFAHLLSMDNPSWRDCVIRDLIL
GKEFLFVVDGFDELTFPAG----ALIRDLCGDWNTVKPVEVLLGSLLKRKMAPHATLLVT
TRTQSLHQIFVMMDQPLLVETLGFLEQEKQEYFQKYFEDEEGEEEDKGEGKALRALKEVR
CNADLYQMASLPTACGIFCLCLELRMKKGEDLSLTCQTYTSMFLNFLCEVFSSETCED-H
LNEEFQILFKKICILAANSLLE-QVPILCEEDFLTLKLNLNNLHPMVCRHILFKDSS-ST
HCLSFICLGIQQLLAAIIFVQELGQESKGVSKYS----------IQNMLSREARLKNPDL
SGLLPFVFGLLNETRIQELKTTFGCQISTEVKRKFLECESGENKP---------------
-------------LLLLMNMQEILSCLYESQEEGFVKEAMVLFE----------------
-DISLHLKTSTDLIHAS-FCLKNSQNLQTMSLKVEKAVFPENVAALESTAKHQRS-----
----PDEQRMLTFWTDFCDTFNSNKKLVFLDIHESFLNSSALEILCEKLPSA------SC
CLQKVVLKNISPDDAYEKLCLIFNGYKTISHLILQGGNLDS--MHHSLCEVLKNPACNLK
FLS---------------------LGSCSTAAQKWDDFFPVLKVNQSLIFLDLTDNSLLD
KSAKLLCNIWKEPKCILQRVSLENCQLTEACCKDLSSVLMVSRTLTHLSLANNKLGDNGV
KNL---------------------------------------------------------
-------------CESISCTECNLQTLVLWSCNITNAGCHYLSKMLKQTLSLKHLDLGLN
RIGTKGAKFLCEALKNPKSKLKSLWLCGCSITPLNCQDFSETLRSNKSLNTLDLSQNVLG
TDAIKTFCEALKLQICPLQMLRLKFDEAKPSIQNLIQEMKVSHPQLRISSDQVDLKNP--
PLPHFIF-----------------------------------------------------
------------------------------------------------------------
------------------------------------------------------------
------------------------------------------------------------
-----------------

#NLRP2_rat "gi|109461167|ref|XP_001070923.1| PREDICTED: similar to NACHT,
leucine rich repeat and PYD containing 2 [Rattus norvegicus]"
------------------------------------------------------------
------------------------------------------------------------
-----------------------MEHSDQLGFNLKDVLKKLDKTELNNFKSTLKSCSLPQ
TMK-------QISKLTMDLANGAQLAEILTDYCPNDWVKRVTFKILEEINRADLAELVMK
QVEE-------------------------------------------AASKASEEKVSSK
PREPLKTSLFPQDFVQGDERPEHKHKK--------------------------------E
WRNMYTEKWKKNFWPKCNKEIYVDTESYRTLLTLCNP-KIEMPFAYTIVLHGPPGSGKTT
MARRLMLEWSE-SKQAQSFSCAFYISCRKLN-NSKPCTFAHLLSMNNPSWRYYMIRRLVL
SEQFLFVVDGFDELTFPAG----ALLRDICGDWNTVKPVEVLLSSLLMRKMEPHSTLLVT
TQTQSLHQIYLMVDQPVLVETQGFLEPEKQEYFQQYFEDEEGEEEGEGEGKALRALKEVK
CNEALFQMASLPAACGIFCLCLELGMKKGEDLALTCQTYTSMFLNFLCRVFLQEACDG-H
LNKQLQILFKKICILAADSLLE-QVPILCEEDFLKLKLNLNKLHPVVYRCIFLEDSS-CT
NCLSFICLSIQQLLAAIIFVQELGQESKDVSKYS----------IQKMLSREARLKNPNL
SGVLPFVFSLLNKTHIQELETIFGCQISMGVKRKFLECELGENKP---------------
-------------FLLLMDLQEIFSCLYESQEEEFVKEAMALFG----------------
-EVSLHLKTNMDLVHAS-FCLKNSQNLQTMFLKVEKAVFPENFATLESTAEDQRF-----
----QDKKCMLTLWTDFCDTFHSNEKLAFLEIHESFLNSSSLEILCEKLSSA------PC
HLQKVVLKNISPDDAYEKLCLIFCGYETISHLTLQGGKLDS--MLHSLCKVVKNPACNLK
```

FLS---------------------LGSCSTTAQKWDDFFPALKANQSLISLDLTDNNLLD
KGVRLLCNTWKQSKCILQRVSLENCHLTEVCCKDLSSVLMVSQTLTHLSLAKNELGDNGV
KKL---
-------------CESLSFPKCKLQTLVLWSCNITSNGCHYLSKMLGQAPSLKHLDLGLN
RIGTTGAKFLCEALKSPRSNLKSLWLCGCSIIPTNCKDFSEILRSNKSLNTLDLSQNVMG
TDAVKIFCESLKLQICPLQTLRLKFDEANPSIQKLIQEMKESHPQLSITSDQVDPKKKSH
SFPHFIF---

#NLRP2_dog "ENSCAFT00000004144 peptide:ENSCAFP00000003831
pep:NOVEL_protein_coding"

-----------------------MEPSAQLGFNLQPLLEQFAQDELSQFKSLLRPLSLQD
ELQ-------HIPATEVEEADGKQLAEILINHCPSHWVEMVTIQIFDKMNRTDLSERAKD
ELRV---KIMKRSNHRKPEE
TWITRTTELKCANLEEPPEHVEEKDSAFTQVQEEDVTNPEEAKEGLKGEKPGKQDKYISI
LKEKVQQMWKKNFWPGASENIHTVTQKYETLIPFCNPKMLAGPFPHTVVLHGPAGVGKTT
LAKKCMLDWTE-DSLAQTCPTAFYLSCKALS-RKGMCSFIELLAQSAPDLQDALAQVLTQ
AQKLLFVIDAFEELRVPSGV--EALVHDLCVDWKTQRPASTLLGSLLKRKLLPTAALLVT
TRPEALRELRLMVEQPLFVEIEGLSECDRKAYFLRHFGEEA---------QALRAFHLMK
SNATLFQMGAAPAVCWMVCTCLKLQMDEGEDPGPTCQTTTSLLLRFLCSQFAPAQGS--C
LGLHLRSTLHALCLLATEGVWT-QTSVFDGHDLLRLGVHESDLHPFLNRNILQKSRD-CE
GCYCFLHLSVQQFLAAALYILGSQDHQDLPAGSPS----WDAGNVQRLLSKEEGLKNPYL
APVGHFLFGLTNEKRARELGMTFGCLVSTKVKQELLECRVGSPEN---------------
-----------RPFSSVTDTKETLHCLYESQEEQLVKEAMVQVT----------------
-EVSLVFKDTSDIMHAS-FCLKHCESLQKMWLQIEKGMFLENDAVSESEDQADRS-----
----QNDQHVLPFWIDFCSMFDSNKNLIFLDISQSFLSASSVRILCEKITSA------TH
NLQKVVLKNIFPADAYRNFCIAFGGHETLTHLTLQGNDQND--MLPILCEILRHPKCNLQ
YLR---------------------LVSCSATTQQWAHLSSSLKINQSLTCLNLTANELPD
ESAKLLCTTLRHPKCFLQRLSLENCQLTEACCKELSSALIVNQRLTHLSLAKNTLGDDGV
KLL---
-------------CEGLSYPDCQLQMLVLWYCSITSGGCNHLSTLLQQNSNLTHLDLGLN
HIGITGLKFLCEALKNPMCNLKCLWLWGCAITPFSSEILSSALGRNQSLVTLDLGQNSLG
YSGIKMLCDTLKLHCSSLRTLRLKIDESDPRIQKLLKEIQEYDPQLTIESDDQDPTNNRP
SSHDFIF---

#NLRP7_human "gi|75709196|ref|NP_996611.2| NACHT, leucine rich repeat and PYD
containing 7 isoform 2 [Homo sapiens]"

------------------------MTSPQLEWTLQTLLEQLNEDELKSFKSLLWAFPLED
VLQ-------KTPWSEVEEADGKKLAEILVNTSSENWIRNATVNILEEMNLTELCKMAKA
EMME---DGQVQEIDNPELG
DAEEDSELAKPGEKEGWR--NS
MEKQSLVWKNTFWQGDIDNFHDDVTLRNQRFIPFLNPRTPRKLTPYTVVLHGPAGVGKTT
LAKKCMLDWTD-CNLSPTLRYAFYLSCKELS-RMGPCSFAELISKDWPELQDDIPSILAQ
AQRILFVVDGLDELKVPP----GALIQDICGDWEKKKPVPVLLGSLLKRKMLPRAALLVT
TRPRALRDLQLLAQQPIYVRVEGFLEEDRRAYFLRHFGDED---------QAMRAFELMR
SNAALFQLGSAPAVCWIVCTTLKLQMEKGEDPVPTCLTRTGLFLRFLCSRFP--------
QGAQLRGALRTLSLLAAQGLWA-QMSVFHREDLERLGVQESDLRLFLDGDILRQDRV-SK
GCYSFIHLSFQQFLTALFYALEKEEGEDRDGHA------WDIGDVQKLLSGEERLKNPDL
IQVGHFLFGLANEKRAKELEATFGCRMSPDIKQELLQCKAHLHAN---------------
------------KPLSVTDLKEVLGCLYESQEEELAKVVVAPFK----------------

```
-EISIHLTNTSEVMHCS-FSLKHCQDLQKLSLQVAKGVFLENYMDFELDIEFERCTYLTI
PNWARQDLRSLRLWTDFCSLFSSNSNLKFLEVKQSFLSDSSVRILCDHVTRS------TC
HLQKVEIKNVTPDTAYRDFCLAFIGKKTLTHLTLAGHIEWERTMMLMLCDLLRNHKCNLQ
YLR---------------------LGGHCATPEQWAEFFYVLKANQSLKHLRLSANVLLD
EGAMLLYKTMTRPKHFLQMLSLENCRLTEASCKDLAAVLVVSKKLTHLCLAKNPIGDTGV
KFL---------------------------------------------------------
-------------CEGLSYPDCKLQTLVLQQCSITKLGCRYLSEALQEACSLTNLDLSIN
QIARG-------------------------------------------------------
---LWILCQALENPNCNLKHLRLKTYETNLEIKKLLEEVKEKNPKLTIDCNASGATAPPC
CDFFC-------------------------------------------------------
------------------------------------------------------------
------------------------------------------------------------
------------------------------------------------------------
-----------------
```

```
#NLRP7_chimpanzee "gi|114678963|ref|XP_512902.2| PREDICTED: NACHT, leucine rich repeat and PYD containing 7 [Pan troglodytes]"
------------------------------------------------------------
------------------------------------------------------------
------------------------MTSPQLEWTLQTLLEQLNEDELKSFKSLLWAFPLED
VLQ-------KTPWSEVEEADGKKLAEILVNTSSENWIRNATVNILEEMNLTELCKMAKA
EMME-------------------------------------------DGQVQEIDNPELG
DAEEDSELAKPGEKEGWR----------------------------------------NS
MEKQSSVWKNTFWQGDIDNFHDAVTLRNQRFIPFLNPRTPGKLTPYTVVLHGPAGVGKTT
LAKKCMLDWTD-CNLSPTVRYAFYLSCKELS-RMGPCSFAELISKDWPELQDDIPSILAQ
AQRILFVVDGLDELKVPP----GALIQDICGDWEKKKPVPVLLGSLLKRKMLPKAALLVT
TRPRALRDLQLLAQQPIYVRVEGFLEEDRRAYFLRHFGDED---------QAMRAFELMR
SNAALFQLGSAPAVCWIVCTTLKLQMEKGEDPVPTCLTRTGLFLRFLCSRFP--------
QGAQLRGALRTLSLLAAQGLWA-QMSVFHREDLERLGVQESDLRLFLDGDILRQDRV-SK
GCYSFIP---------------------------------------------------DL
IQVGHFLFGLANENRAKELEATFGCRMSPDIKQELLRCKAHLHAN---------------
------------KSLSVTDLREVLGCLYESQEEELAKVVVAPLK----------------
-EISIHLTNTSEVRHCS-FSLKHCQDLQKLSLQVAKGVFLENYMDFELDIEFERCTYLTI
PTWARQDLRSLRLWTDFCSLFSSNSNLKFLEVKQSFLSDSSVRILCDHVTRS------TC
HLQKVEIK------------------NTLTHLTLAGHIEWERTMMLMLCDLLRNRKCNLQ
YLR---------------------L-----------------------------------
----------------------ENCRLTEASCKDLAAVLVVSKKLTHLCLAKNPIGDTGV
KFL---------------------------------------------------------
-------------CEGSSYPDCKLQTLV--------------------------------
------------------------------------------------------------
------------------------------------------------------------
------------------------------------------------------------
------------------------------------------------------------
------------------------------------------------------------
------------------------------------------------------------
-----------------
```

```
#NLRP3_human "gi|34878693|ref|NP_004886.3| NLR family, pyrin domain containing 3 isoform a [Homo sapiens]"
------------------------------------------------------------
------------------------------------------------------------
------------------------MKMASTRCKLARYLEDLEDVDLKKFKMHLEDYPPQK
GCIPLPRG-------QTEKADHVDLATLMIDFNGEEKAWAMAVWIFAAINRRDLYEKAKR
DE-----PKWGSD----------------------------NARVSNPTVICQEDSIEEE
WMGLLEYLSRISICKMKKDYRKKYRKYVRSRFQCIEDRNARLGESVSLNKRYTRLRLIKE
HRSQQEREQELLAIG--KTKTCESPVSPIKMELLFDPDDEHSEPVHTVVFQGAAGIGKTI
LARKMMLDWASGTLYQDRFDYLFYIHCREVS-LVTQRSLGDLIMSCCPDPNPPIHKIVRK
PSRILFLMDGFDELQGAFDE----HIGPLCTDWQKAERGDILLSSLIRKKLLPEASLLIT
TRPVALEKLQHLLDHPRHVEILGFSEAKRKEYFFKYFSDEA---------QARAAFSLIQ
ENEVLFTMCFIPLVCWIVCTGLKQQMESGKSLAQTSKTTTAVYVFFLSSLLQPRGGS---
QEHGLCAHLWGLCSLAADGIWN-QKILFEESDLRNHGLQKADVSAFLRMNLFQKEVD-CE
```

KFYSFIHMTFQEFFAAMYYLLEEEKEGRTN--VPGSRLKLPSRDVTVLLENYGKFEKGYL
IFVVRFLFGLVNQERTSYLEKKLSCKISQQIRLELLKWIEVKAKA---------------
-----------KKLQIQPSQLELFYCLYEMQEEDFVQRAMDYFP----------------
-KIEIN-LSTRMDHMVSSFCIENCHRVESLSLGFLHNMPKEEEEEEKE-----GRHLD--
---MVQCVLPSSSHAACSHGLVNSHLTSSFCRG---------------------------
------------------LFSVLSTSQSLTELDLSDNSLGD-PGMRVLCETLQHPGCNIR
RLW---------------------LGRCGLSHECCFDISLVLSSNQKLVELDLSDNALGD
FGIRLLCVGLKHLLCNLKKLWLVSCCLTSACCQDLASVLSTSHSLTRLYVGENALGDSGV
AIL---
-------------CEKAKNPQCNLQKLGLVNSGLTSVCCSALSSVLSTNQNLTHLYLRGN
TLGDKGIKLLCEGLLHPDCKLQVLELDNCNLTSHCCWDLSTLLTSSQSLRKLSLGNNDLG
DLGVMMFCEVLKQQSCLLQNLGLSEMYFNYETKSALETLQEEKPELTVVFEPSW------

#NLRP3_chimpanzee "ENSPTRT00000004039 peptide:ENSPTRP00000003727 pep:NOVEL_protein_coding"

------------------------MKMASTRCKLARYLEDLEDVDLKKFKMHLEDYPPQK
GCIPLPRG-------QTEKADHVDLATLMIDFNGEEKAWAMAVWIFAAINRRDLYEKAKR
DE-----PKWGSD----------------------------NACVSNPTVICQEDSIEEE
WMGLLEYLSRISICKMKKDYRKKYRKYVRSRFQCIEDRNARLGESVSLNKRYTRLRLIKE
HRSQQEREQELLAIG--KTKACESPVSPIKMEMLFDPDDEHSEPVHTVVFQGAAGIGKTI
LARKIMLDWALGTLYQDRFDYLFYIHCREVS-LVTQRSLGDLIMSCCPDPNPPIHKIVRK
PSRILFLMDGFDELQGAFDE----HIGPLCTDWQKAERGDILLSSLIRKKLLPEASLLIT
TRPVALEKLQHLLDHPRHVEILGFSEAKRKEYFFKYFSDEA---------QARAAFSLIQ
QNEVLFTMCFIPLVCWIVCTGLKQQMESGKSLAQTSKTTTAVYVFFLSSLLQPRGGS---
QEHRLCAHLWGLCSLAADGIWN-QKILFEESDLRNHGLQKADVSAFLRMNLFQKEVD-CE
KFYSFIHMTFQEFFAAMYYLLEEEKEGRTN--VPGSRLKLPSRDVTVLLENYGKFEKGYL
IFVVRFLFGLVNQERTSYLEKKLSCKISQQIRLELLKWIEVKAKA---------------
-----------KKLQIQPSQLELFYCLYEMQEEDFVQRAMDYFP----------------
-KIEIN-LSTRMDHVVSSFCIENCHRVESLSLGFLHNMPKEEEEEEKE-----GRHPD--
---MVQCVLPGSSHAACSHGLG--------------------------------------

--------------------------RCGLSHECCFDISLVLSSNQKLVELDLSDNALSD
FGIRLLCVGLKHLLCNLKKLWLVN------------------------------------

-------------------------------SGLTSVCCSALSSVLSTNPNLTHLYLRGN
TLGDKGIKLLCEGLLHPDCKLQVLELDNCNLTSHCCGDLSTLLTSSQSLRKLSLGNNDLG
DLGVMMFCEVLKQQSCLLQNLGLSEMYFNYETKSALETLQEEKPELTVVFEPSW------

#NLRP3_rat "gi|109488152|ref|XP_220513.4| PREDICTED: similar to cold autoinflammatory syndrome 1 homolog [Rattus norvegicus]"

--------------------------MMSVRCKLAQYLEDLEDVDLKKFKMHLEDYPPEK
GCVPIPRG-------QMEKADHLDLATLMIDFNGEEKAWGMAVWIFAAINRRDLWEKAKK
DQ-----PEWNDA------------------------------CTSNLSMVCQEDSLEEE
WIGLLGYLSRISICKKKKDYCKIYRRHVRSRFYSIKDRNARLGESVDLNRRYTQLQLVKE
HPSKQEREHELLTIG--RTKMWDRPMSSLKLELLFEPEDEHLEPVHTVVFQGAAGIGKTI
LARKIMLDWALGKLFKDKFDYLFFIHCREVS-LRAPKSLADLIISCWPDPNPPVCKILCK
PSRILFLMDGFDELQGAFDE----HIEEVCTDWQKAVRGDILLSSLIRKKLLPKASLLIT

```
TRPVALEKLQHLLDHPRHVEILGFSEAKRKEYFFKYFSNEL---------QAREAFRLIQ
ENEILFTMCFIPLVCWIVCTGLKQQMETGKSLAQTSKTTTAVYVFFLSSLLQSRGGI---
EEHLFSAYLPGLCSLAADGIWN-QKILFEECDLRKHGLQKTDVSAFLRMNVFQKEVD-CE
RFYSFSHMTFQEFFAAMYYLLEEEEEGVTVRKGPEGCSDLLNRDVKVLLENYGKFEKGYL
IFVVRFLFGLVNQERTSYLEKKLSCKISQQVRLELLKWIEVKAKA---------------
-----------KKLQRQPSQLELFYCLYEMQEEDFVQSAMGHFP----------------
-KIEIN-LSTRMDHVVSSFCIKNCHRVKTLSLGFLHNSPKEEEEEKRG-----SQPLD--
---QVQCVFPDPHVACSSRLVNCCLTSSFCRG----------------------------
------------------LFSSLSTNQSLTELDLSDNTLGD-PGMRVLCEALQHPGCNIQ
RLW---------------------LGRCGLTHQCCFNISSVLSSSQKLVELDLSDNALGD
FGVRLLCVGLKHLLCNLQKLWLVSCCLTSACCQDLALVLSSNHSLTRLYIGENALGDSGV
QVL---------------------------------------------------------
-------------CEKMKDPQCNLQKLGLVNSGLTSLCCSALTSVLKTNQNLTHLYLRSN
ALGDMGLKLLCEGLLHPDCKLQMLELDNCSLTSHSCWDLSTILTHNQSLRKLNLSNNDLG
DLCVVTLCEVLKQQGCLLQSLQLGEMYLNCETKRTLEALQEEKPELTVVFEISW------
------------------------------------------------------------
------------------------------------------------------------
------------------------------------------------------------
------------------------------------------------------------
-----------------

#NLRP3_mouse "gi|22003870|ref|NP_665826.1| NLR family, pyrin domain containing 3
[Mus musculus]"
------------------------------------------------------------
------------------------------------------------------------
--------------------------MTSVRCKLAQYLEDLEDVDLKKFKMHLEDYPPEK
GCIPVPRG-------QMEKADHLDLATLMIDFNGEEKAWAMAVWIFAAINRRDLWEKAKK
DQ-----PEWNDT------------------------------CTSHSSMVCQEDSLEEE
WMGLLGYLSRISICKKKKDYCKMYRRHVRSRFYSIKDRNARLGESVDLNSRYTQLQLVKE
HPSKQEREHELLTIG--RTKMRDSPMSSLKLELLFEPEDGHSEPVHTVVFQGAAGIGKTI
LARKIMLDWALGKLFKDKFDYLFFIHCREVS-LRTPRSLADLIVSCWPDPNPPVCKILRK
PSRILFLMDGFDELQGAFDE----HIGEVCTDWQKAVRGDILLSSLIRKKLLPKASLLIT
TRPVALEKLQHLLDHPRHVEILGFSEAKRKEYFFKYFSNEL---------QAREAFRLIQ
ENEVLFTMCFIPLVCWIVCTGLKQQMETGKSLAQTSKTTTAVYVFFLSSLLQSRGGI---
EEHLFSDYLQGLCSLAADGIWN-QKILFEECDLRKHGLQKTDVSAFLRMNVFQKEVD-CE
RFYSFSHMTFQEFFAAMYYLLEEEAEGETVRKGPGGCSDLLNRDVKVLLENYGKFEKGYL
IFVVRFLFGLVNQERTSYLEKKLSCKISQQVRLELLKWIEVKAKA---------------
-----------KKLQWQPSQLELFYCLYEMQEEDFVQSAMDHFP----------------
-KIEIN-LSTRMDHVVSSFCIKNCHRVKTLSLGFFHNSPKEEEEERRG-----GRPLD--
---QVQCVFPDTHVACSSRLVNCCLTSSFCRG----------------------------
------------------LFSSLSTNRSLTELDLSDNTLGD-PGMRVLCEALQHPGCNIQ
RLW---------------------LGRCGLSHQCCFDISSVLSSSQKLVELDLSDNALGD
FGIRLLCVGLKHLLCNLQKLWLVSCCLTSACCQDLALVLSSNHSLTRLYIGENALGDSGV
QVL---------------------------------------------------------
-------------CEKMKDPQCNLQKLGLVNSGLTSICCSALTSVLKTNQNFTHLYLRSN
ALGDTGLRLLCEGLLHPDCKLQMLELDNCSLTSHSCWNLSTILTHNHSLRKLNLGNNDLG
DLCVVTLCEVLKQQGCLLQSLQLGEMYLNRETKRALEALQEEKPELTIVFEISW------
------------------------------------------------------------
------------------------------------------------------------
------------------------------------------------------------
------------------------------------------------------------
-----------------

#NLRP3_dog "gi|73962271|ref|XP_848377.1| PREDICTED: similar to Cold
autoinflammatory syndrome 1 protein (Cryopyrin) (NACHT-, LRR- and PYD-containing
protein 3) (PYRIN-containing APAF1-like protein 1) (Angiotensin/vasopressin
receptor AII/AVP-like) isoform 3 [Canis familiaris]"
------------------------------------------------------------
------------------------------------------------------------
--------------------------MASVRCKLARYLEDLEDADFKKFKMHLQDYPSQK
GFSPLPRS-------QTEKADHMDLATLMIDFNGEEKAWAMAVWIFAAINRRDLYEKAKR
```

DE-----LEWGSG----------------------------CPRLYNSHLECREESLEEE
WMGLLGYLSRISICKKKKDYCKKYKKHVRSRFQCIKDRNARLGETVNLNKRYTRLRLVKE
HQSQQEREHELLAIGRTSAKTLDSPMSSVNVELLFEPDDQHLEPVHTVVFQGSAGIGKTI
LARKIMLDWASDKIYQDRFDYLFYIHCREVS-LGTRRSLGDLIVSCCPDPNPPICKIVSK
PSRILFLMDGFDELQGAFDE----HTEALCTNWQKVERGDILLSSLIRKRLLPEASLLIT
TRPVALEKLQHLLDRPRHVEILGFSEAKRKEYFFKYFSDEQ---------QATEAFRLIQ
ENEILFTMCFIPLVCWIVCTGLKQQMDSGKNLAQTSKTTTSVYIFFLSSLLQSHRET---
QKHQVSASLRGLCSLAADGIWN-QKILFDECDLRNHGLQKADVSAFLRMNLFQKEVD-CE
KFYSFIHMTFQEFFAAMYYLLEEEQGRMRN-LPWSSSKLPNRDVKVLLENYGKFEKGYL
IFVVRFLFGLVNQERTSYLEKKLSCKISQQIRLELLKWIEEKAKG---------------
-----------KNLQIQPSQLELFYCLYEMQEEDFVRKAMGHFP----------------
-KIEIS-LSTRMDHVVSSFCIENCCRVESLSLRLLHNSPKEEEEEEEEENEKEMQHYD--
---VDHCVLLDPHTTYAYRLVNCQVTVSLWQGL---------------------------
------------------FSVLSRNSLSLTELNLSDNALGD-QGVNVLCEMLQHPGCNIR
KLW---------------------LGQCCLSYRCCFNISSVLSNNQKLEELDLSHNALGD
FGIRLLCVGLKHLFCNLNKLWLVSCCLTPACCEDLASVLSTNQSLTRLYLGENSLEDAGV
GVL---
-------------CEKAKHPQCKLQRLGLVNSGLTPGCCPALASMLSTNQKLTHLYLRGN
ALGDTGVKLLCEGLLHPNCKLQILELDGCSLTSHCCWDLSTLLTSSKSLRELSLGSNDLG
DLGVMLLCEVLKQQGCILQSLKLCEMYFNYDTKCALETLQEEKPELTIVFEPFGSDRSRL
PDASVWAPCHLGVHKWERALLHPG------------------------------------

#NLRP3_bovine "gi|76625172|ref|XP_581687.2| PREDICTED: similar to cryopyrin [Bos taurus]"

------------------------MRMVSVRCKLARYLEDLEDIDFKKFKMHLEDYPSQK
GCTSIPRG-------QTEKADHVDLATLMIDFNGEEKAWAMAKWIFAAINRRDLYEKAKR
EE-----PEWGEWN---------------------------EDKNANISVLSQEESLEEE
WMGLLGYLSRISICRKKKDYCKKYRKYVRSKFQCIKDRNARLGESVNLNKRFTRLRLIKE
HRSQQEREHELLAIGRTWAKIQDSPVSSVNLELLFDPEDQHSEPVHTVVFQGAAGIGKTI
LARKIMLDWASEKLYQDRFDYLFYIHCREVS-LGTQRSLGDLIASCCPGPNPPIGKIVSK
PSRILFLMDGFDELQGAFDE----HTEALCTNWRKVERGDILLSSLIRKRLLPEASLLIT
TRPVALEKLQHLLGQARHVEILGFSEARRKEYFLKYFSDEQ---------QAREAFRLIQ
ENEILFTMCFIPLVCWIVCTGLKQQMDSGKSLARTSKTTTAVYIFFLSSLLQSQGGS---
QENHNSATLWGLCSLAADGIWN-QKILFQECDLRNHGLQKADVSAFLRMNLFQKEVD-CE
KFYSFIHMTFQEFFAAMYYLLEEDNHGEMRN-TPQACSKLPNRDVKVLLENYGKFEKGYL
IFVVRFLFGLINQERTSYLEKKLSCKISQKIRLELLKWIEAKANA---------------
-----------KTLQIEPSQLELFYCLYEMQEEDFVQRAMSHFP----------------
-KIEIK-LSTRMDHVVSSFCIENCRHVESLSLRLLHNSPKEEEEEEE------VRHSH--
---MDRSVLSDFEVAYSQGLVNYLTSSICRG-----------------------------
------------------IFSVLSNNWNLTELNLSGNTLGD-PGMNVLCETLQQPGCNIR
RLW---------------------LGQCCLSHQCCFNISSVLSNNQKLVELDLSHNALGD
FGIRLLCVGLRHLFCNLKKLWLVSCCLTSASCEDLASVLSTNHSLTRLYLGENALGDSGV
GIL---
-------------CEKVKNPHCNLQKLGLVNSGLTSGCCPALSSVLSTNQNLTHLYLQGN
ALGDMGVKLLCEGLLHRNCKLQVLELDNCSLTSHCCWDLSTLLTSNQSLRKLCLGNNDLG
DLGVMLLCEVLKQQGCLLKSLRLCEMYFNYDTKRALETLQEEKPELTIVFEPSR------

#NLRP6_chimpanzee "gi|114635347|ref|XP_521701.2| PREDICTED: NACHT, leucine rich repeat and PYD containing 6 [Pan troglodytes]"

```
------------------------------------------------------------
------------MDQPEAPCSSTGPRLAVARELLLAALEELSQEQLKRFRHKLRDVGPDG
RS--IPWGR-------LERADAVDLAEQLAQFYGPEPALEVARKTLKRADARDVAAQLQE
QRLQREFCAGGP------------------------------------------------
--------SCLGRGWAALLPASRRSPGAVPAAARVKERNARSVKITKRFTKLLIAPESAA
PEEEAL--GPAEEPEPGRARR----SDTHTFNRLFR-RDEEGRRPLTVVLQGPAGIGKTM
AAKKILYDWAAGKLYQGQVDFAFFLPCGELLERPGTRSLADLILDQCPDRGAPVPQMLAQ
PQRLLFILDGADELPALGGP----EAAPCTDPF-EAASGARVLGGLLSKALLPTALLLVT
TRAAAPGRLQGRLCSPQCAEVRGFSDKDKKKYFYKFFRDER---------RAERAYRFVK
ENETLFALCFVPFVCWIVCTVLRQQLELGRDLSRTS-KTTTSVYLLFITSVLSSAPVADG
PRLQG--DLRNLCRLAREGVLG-RRAQFAEKELEQLELRGSKVQTLFLSKKELPGVLETE
VTYQFIDQSFQEFLAALSYLLEDGGVPR-TAAGGV--------GTLLRGD---TQPHSHL
VLTTRFLFGLLSAERMRDIQRHFGCMVSERVKQEALRWVQGQGQ--GCPGVAPEVTEGAK
GLEDTEEPEEEEEGEEPNYPLELLYCLYETQEDAFVRQALCGLP----------------
ELALQRVRFCRMDVAVLSYCVRCCPAGQALRLISCRLVAAQEKKKKSLGKRLQAS-----
-----------------LGGSSSSRGTTK------QLPASLLHPLFQAMTDP------LC
HLSSLTLSHCKLPDAVCRDLSEALRAAPALTELGLLHNRLSEAGLRMLSEGLAWPQCRVQ
TVR---------------------VQLPDPQRGLQ-YLVSVLRQSPALTTLDLSGCQLPA
PMVTYLCAVLQHQGCGLQTLSVAQQPPELCPCRLEVGSSPSLPVRNETIPEAMAQAQRRP
VSEIFGKGD---------------------------------------------------
------------------------------------------------------------
------------------------------------------------------------
------------------------------------------------------------
------------------------------------------------------------
------------------------------------------------------------
------------------------------------------------------------
------------------------------------------------------------
-----------------

#NLRP6_rat "gi|109459602|ref|XP_577848.2| PREDICTED: similar to NACHT-, LRR- and
PYD-containing protein 6 (PYRIN-containing APAF1-like protein 5-like)
(Angiotensin II/vasopressin receptor) [Rattus norvegicus]"
------------------------------------------------------------
------------------------------------------------------------
-MCDPGGSGTGPIQVASVITLPIPSSEAVARELLLAALQDLSQEQLKRFRHKLRDAPLDG
RS--IPWGR-------LEHSDAVDLTDKLIEFYAPEPAVDVTRKILKKADIRDVSLRLKE
QQLQRLGSSSA-------------------------------------------------
-------------LLTVSEYKKKYREHVLRQHAKVKERNARSVKINKRFTKLLIAPGSGA
GEDELLG--TSGEPEPERARR----SDTHTFNRLFRGNDDEGPRPLTVVLQGPAGIGKTM
AAKKILYDWAGGKLYHSQVDFAFFMPCGELLERPGTRSLADLILEQCPDRTAPVRRILAQ
PHRLLFILDGADELPTLAAP----EATPCRDPF-EATSGLRVLSGLLSQELLPSARLLVT
SRNATLGRLQGRLCSPQCAEVRGFSDKDKKKYFFKFFRDER---------KAERAYRFVK
ENETLYALCFVPFVCWIVCTVLLQQMELGRDLSRTS-KTTTSVYLLFITSMLKSAGTN-G
PRVQG--ELRMLCRLAREGILK-HQAQFSEKDLERLKLQGSQVQTMFLSKKELPGVLETV
VTYQFIDQSFQEFLAALSYLLDAEGAPG-NSAGSV--------QMLLNSD---AGLRGHL
ALTTRFLFGLLSTERIRDIGNHFGCVVPGRVKQDTLRWVQGQSQPKVATVGAEKKDELKD
EEAEEEEEEEEEEEEEELNFGLELLYCLYETQEDDFVRQALSSLP----------------
EMVLERVRLTRMDLEVLSYCVQCCPDGQALRLVSCGLVAAKEKKKKKKSFRR--------
--------------QPCSFSPSSSQSTGK------QPPASLLRPLCEAMITQ------QC
GLSILTLSHCKLPDAVCRDLSEALKVAPSLRELGLLQNRLTEAGLRLLSQGLAWPKCKVQ
TLR---------------------IQMPGLQEVIH-YLVIVLQQSPVLTTLDLSGCQLPG
TVVEPLCSALKHPKCGLKTLSLTSVELTENPLRELQAVKTLKPDLAIIHSKLGTHPQPLK
G-----------------------------------------------------------
------------------------------------------------------------
------------------------------------------------------------
------------------------------------------------------------
------------------------------------------------------------
------------------------------------------------------------
------------------------------------------------------------
------------------------------------------------------------
-----------------
```

#NLRP6_human "gi|21264320|ref|NP_612202.1| NLR family, pyrin domain containing 6 [Homo sapiens]"

```
------------------------------------------------------------
------------------------------------------------------------
------------MDQPEAPCSSTGPRLAVARELLLAALEELSQEQLKRFRHKLRDVGPDG
RS--IPWGR-------LERADAVDLAEQLAQFYGPEPALEVARKTLKRADARDVAAQLQE
RRLQRLGLGSG-------------------------------------------------
------------TLLSVSEYKKKYREHVLQLHARVKERNARSVKITKRFTKLLIAPESAA
P-EEAL--GPAEEPEPGRARR----SDTHTFNRLFR-RDEEGRRPLTVVLQGPAGIGKTM
AAKKILYDWAAGKLYQGQVDFAFFMPCGELLERPGTRSLADLILDQCPDRGAPVPQMLAQ
PQRLLFILDGADELPALGGP----EAAPCTDPF-EAASGARVLGGLLSKALLPTALLLVT
TRAAAPGRLQGRLCSPQCAEVRGFSDKDKKKYFYKFFRDER---------RAERAYRFVK
ENETLFALCFVPFVCWIVCTVLRQQLELGRDLSRTS-KTTTSVYLLFITSVLSSAPVADG
PRLQG--DLRNLCRLAREGVLG-RRAQFAEKELEQLELRGSKVQTLFLSKKELPGVLETE
VTYQFIDQSFQEFLAALSYLLEDGGVPR-TAAGGV--------GTLLRGD---AQPHSHL
VLTTRFLFGLLSAERMRDIERHFGCMVSERVKQEALRWVQGQGQ--GCPGVAPEVTEGAK
GLEDTEEPEEEEEGEEPNYPLELLYCLYETQEDAFVRQALCRFP----------------
ELALQRVRFCRMDVAVLSYCVRCCPAGQALRLISCRLVAAQEKKKKSLGKRLQAS-----
-----------------LGGGSSSQGTTK------QLPASLLHPLFQAMTDP------LC
HLSSLTLSHCKLPDAVCRDLSEALRAAPALTELGLLHNRLSEAGLRMLSEGLAWPQCRVQ
TVR---------------------VQLPDPQRGLQ-YLVGMLRQSPALTTLDLSGCQLPA
PMVTYLCAVLQHQGCGLQTLSLASVELSEQSLQELQAVKRAKPDLVITHPALDGHPQPPK
ELISTF------------------------------------------------------
------------------------------------------------------------
------------------------------------------------------------
------------------------------------------------------------
------------------------------------------------------------
------------------------------------------------------------
------------------------------------------------------------
------------------------------------------------------------
-----------------
```

#NLRP6_mouse "gi|124487067|ref|NP_001074858.1| NLR family, pyrin domain containing 6 [Mus musculus]"

```
------------------------------------------------------------
------------------------------------------------------------
---------------MDAAGASCSSVDAVARELLMATLEELSQEQLKRFRHKLRDAPLDG
RS--IPWGR-------LERSDAVDLVDKLIEFYEPVPAVEMTRQVLKRSDIRDVASRLKQ
QQLQKLGPTSV-------------------------------------------------
-------------LLSVSAFKKKYREHVLRQHAKVKERNARSVKINKRFTKLLIAPGTGA
VEDELLG--PLGEPEPERARR----SDTHTFNRLFRGNDEESSQPLTVVLQGPAGIGKTM
AAKKILYDWAAGKLYHSQVDFAFFMPCGELLERPGKRSLADLVLDQCPDRAWPVKRILAQ
PNRLLFILDGADELPTLPSS----EATPCKDPL-EATSGLRVLSGLLSQELLPGARLLVT
TRHAATGRLQGRLCSPQCAEIRGFSDKDKKKYFFKFFRDER---------KAERAYRFVK
ENETLFALCFVPFVCWIVCTVLQQQLELGRDLSRTS-KTTTSVYLLFITSMLKSAGTN-G
PRVQG--ELRTLCRLAREGILDHHKAQFSEEDLEKLKLRGSQVQTIFLNKKEIPGVLKTE
VTYQFIDQSFQEFLAALSYLLEAERTPG-TPAGGV--------QKLLNSD---AELRGHL
ALTTRFLFGLLNTEGLRDIGNHFGCVVPDHVKKDTLRWVQGQSHPKGPPVGAKKTAELED
IEDAEEEEEEE---EDLNFGLELLYCLYETQEEDFVRQALSSLP----------------
EIVLERVRLTRMDLEVLNYCVQCCPDGQALRLVSCGLVAAKEKKKKKKSLVKRLKGSV--
------------TGTNLVLSLYSSQSTKK------QPPVSLLRPLCETMTTP------KC
HLSVLILSHCRLPDAVCRDLSEALKVAPALRELGLLQSRLTNTGLRLLCEGLAWPKCQVK
TLR---------------------MQLPDLQEVIN-YLVIVLQQSPVLTTLDLSGCQLPG
VIVEPLCAALKHPKCSLKTLSLTSVELSENSLRDLQAVKTSKPDLSIIYSK---------
------------------------------------------------------------
------------------------------------------------------------
------------------------------------------------------------
------------------------------------------------------------
------------------------------------------------------------
------------------------------------------------------------
```


#NLRP6_bovine "gi|119919524|ref|XP_581339.3| PREDICTED: similar to PYRIN-containing APAF1-like protein 5 [Bos taurus]"
---MGLGPAAGRACCLWSVCDP
WSGCYSAGRPQRAEETGSDVCGQREATCVGGTRQELGAGKSEEATCPTLLCHFLLTACLT
ATPAVLDLVIDSAGQLEASCSSMEPRAAAARELLLGALEDLSQEQLKRFRHKLRDERVDG
RS--IPWGR-------LERADTLDLVEQLVHFYGPERALDVAKKTLKRADVRDVAARLKE
RRLQSLGPSSP---
------------ALLSVSEYKKKYREHVLRQHAKVKERDARSVKINKRFTKLLIAEE---
-------------PEAERARR----SDTHTFNRLFG-RDEEGERRLTVALQGPAGIGKTM
AARKILYDWAAGKLYHSQVDFAFFLSCRELLERPGTCSLADLILDQCPDREAPVQQMLAQ
SERLLFILDGVDEMLAPEPA----EAAPCRDPH-EAASGARVLGSLLSKELLPAARLLVT
RRGATPGRLQARLCSPQCAEVCGFSDKDRKKYFYKFFQDEW---------KAEHAYRSVK
ENEMLFSLCFVPGVCWIVCTVLRQQLQRGQDLWRTS-KTTTAVYLLFVASVLRSAPAADA
AQLQR--ELRKLCRLACDGVLG-GRARFSEEDLERLQLCGSKVQTQFLSKKEMPGVLETE
VTYQFIDQSFQEFLAALSYLLEDEGAPR-APAGSI--------EALLQGD---PELRGHL
NLTTRFLFGLLNTERMCEIKRHFGCTVSERVKQRVLRWVQDQGQ--GRPRAAPEGTEEIL
APQDTEE-SEEEEGEQLNYPLELLYCLHETQEDAFVHQALRGLP----------------
ELVLERVHFSRMDLAVLSYCVRCCPAGQALRLVSCRLATAQEKKKKSLMKRLQGS-----
-----------------LGG-SSSRATTR------KSPGSPLRPLCEAMTDR------QC
GLNSLTLSRCKLPDSVCRDLSEALREAPALAELGLFHNGLSEAGLRVLSEGLASPQCRVQ
TLR---------------------VQQPGLQEALQ-YMVGKLQQIPALTTLDLSGCQLSG
PTVTCLCTTLKCPGCHLQTLRLTSVELSEQALQELQAVETANPRLVITHPALDAHPKPPT
VPISAL--

#NLRP6_dog "ENSCAFT00000010444 peptide:ENSCAFP00000009685 pep:NOVEL_protein_coding"

------------MDPPEACCRKVRPTLAVARDLLQATFEDLNQEQLKRFRHKLRDATLDG
RN--IPWGR-------LDGLDAMDLAAQLTHFYGPEPALDVTRKTLKRADMLDVAARLKE
QQLQRLGPSSS---
------------TLRSVSEYKKKYRDHVLQQHAKVKERNARSVKINKRFTKLLIAREGAA
LPDEALGCTKELEPEPQRARR----SDTHTFNRLFG-RDEEGQRPLTVVLQGPAGIGKTM
AAKKILYDWAAGKLYHGQVDFAFFLPCRELLERPGACSLADLVLDQCPQRKAPLKPMLAR
PERLLFILDGADDLPPLAP-----HAAPCTDPF-QAADAARVLGGLLGKTLLPAARLLVT
ARTTAPGRLLDRLCSPQCAEVCGFSDKDKKKYFHKFFRDER---------LAERAYRFVK
ENETLFALCFVPFVCWIVCTVLRQQLELGQDLSRTS-KTTTSVYLLFIISVLSSAPAADA
PRMRA--ELRKLCRLAREGTLG-HRAQFAEKDLERLELRGSKVQTLFLSKKELPGVLQTD
VTYQFMDQSFQEFFSALSYLLEEE--------------------RDLRAL---LQGESHL
TLTTRFLFGLLNPDRVSDMQRHFGVAVPERVKQDVLLWMQEQGQ--GRRRAAPEKTAGTD
AAEDAEEPEEEE--CEPNYPLELLYCLYETQEDAFVRQALRCLP----------------
ELELERVRLSRMDLVILSYCVRCCPPGQALRLVSCGLAPAQ-KKKKSLMKRLHSS-----
-----------------LGGSSSSQASKR------KPQPSPLRPLFEAMTDE------HC
GLNSLTLCRCKLPDSVCHDLSEALREAPALTELGLLHNQLSEAGLRVLLEGLAWPQCRVQ
KLR---------------------VQQPSLQEAPQ-YIISILRQS-VASTLYLGDCQLPG
PVVTSLCAALQHPGCRLQTLTLTSVELSKESLKELQAVKTAKPGLVIIHPALDSHPKPSK
GLSDAL--

#NLRP8_bovine "gi|119925441|ref|XP_582571.3| PREDICTED: similar to NOD16 [Bos taurus]"

--MGDMNLSS
DPSSCPSSPLSPSGVSPPSLESSSSSTLPFRNGVMPFMLSVSAEHLQRFKQLLVEENPRP
GCSPLT-------WDQLKSARCGELVHLLTEYFPGQRAWEMARDIFAKMNQTELCLQTQR
ELNEILPNLEPEALSLRKRELTLEEDE---------------------------------
---SDKIREYKARVMS
EHSTLWDRTSWPGNNVDFFYQEPCREDTLLQCLLLPRKPQGRQPK-TVVLQGDAGVGKTT
VAKKVMQQWAENKFYAHKGWLAFYLHCQEMD-RPEEQSFSELIACKLSGSPALASRILSH
PEHLLLLLDGFEELTLTLID----RREDLSEDWSQKMPGSVLLTSLLSKRMLPDATIIVF
LRFSLWKTVGPFLNSPSLITLMGFSAPERSRYFRSYFKNRR---------DADAALRFAM
GNAVLFSMCRVPVICWLVCSCLKQQMERGSSLPRAFPNATAVFIHYLSSLLPTRVRH--V
VGETPQEQLKRLCSLAVEGMWK-NQWVFSEMDLKLARLDDRDVEVFLGVRVLRRAVA-GG
DLIAFAHPSFQEFFAALLFVLCFPQRLRNFQVLD--------RFRIFQLLAHPGRRKNHL
AGMALFLFGLLNDTCALAVEQLFGCKVSLGNKRKLLKVATMPPDG---------------
-----------DPLTPQHGLPQLFYCLHEIREEVFVGQILHNHR----------------
-KALLVISKIRDLQVSA-FCLKFCRQLRELEVTISWAVAKATSLSPGPLPSPQPE-----
-----GSDLRSLWWQDFCSVFKTHESLEVLTVRDSVMDTEAVETLAAALRHP------HC
NLRKLIFKRVGSLVVSEGIIRVLVENQYLRHLQIQDTEVGC-QVIDALCNTLKHPWCFLQ
FLSPYSVRWFFILKVVWRISVERRLEGCPFDPSNGADLTRSLKRNIHLKTLMLRRGSLER
GEE-----CPPVLAPQLERLSLENCDLTPLSCESLAFSLVSNQSLTHLSLAENALQDDGV
KQL---
-------------WNILQHFPCPLQRLVLRNCALTSECCQDIASALDKNKTLRSLDLGAN
RLRDSGVVLLCQPLLNPDSGLQVLELEECQFTSVCCPALTSVLLHNRTLRYLDLSGNGVG
LQGAKLLQDAALKRVLRPEVVLYVPPLRVRQPQAWEGRRLVGRATLGPSCSFLSSLGAHQ
LTFGCTCNR---

#NLRP14_bovine "ENSBTAT00000002954 peptide:ENSBTAP00000002954 pep:NOVEL_protein_coding"

-------------------MTHLSSSSFFSDFGLLLYLEELNKEELIRFKSFLKNETLEP
RSCRIP-------WSEVKKAKRKDLADLMSKYYPGEQAWEVALSIFGKMNLKDLCQRAKA
EINGTAQTMRTEDTEAREVQGDQEAVL---------------------------------
--GDGAEYRIQIRE
KFRNMLDKNHLLGKSGDFCHEIAQEGRELLERLFGEEVGTEEQPQ-TVVLQGAAGIGKTT
LVRMMMLDWAQGNLYQQKFTYVFYLNAREIN-QLRERSFVQLLSKDWPSTEGPIERIMSQ
PSRLLFIIDSFDELNFAFEE----PEFVLCADWTQVHPVSFLMSSLLRKVMLPESSLLVT
TKLTAWRKLKPLLKNRHSIQLLGMSEDARQEYIYQFFEDQS---------WALQVFSCLR
NSVMLFNMCKVPAVCWVVCNCLEQQMEKGADITLTCKTTTSLFACCISSLLTQVDRS--F
PGLPSQTQLRSLCHLAAKGVWT-MTYVFYKENLRRHGLVKSDVSIFLDTNILQKDTE-YE
NCYVFTHLHIQEFFAAMFYVLEGNWDARDDSLQS-------FENLELVLESSSY-KDPHL
SQMKCFLFGLLNEDRMKQLEETFNCTMSLEVKWTILQWME-TLGSS--------------
-----------EQLPSHLVFLELFFHLYETQDEVFVSQAMRYFQ----------------
-KVVINICEEIHLLLSS-FCLKHCQCLRTMKLMVTGVFEKKMLNSSFPPETWQR------
-----AGSHIFHWWQDLCSVLHTNKHLREVILCHSNLDELAMKIFNLELRHP------NC
KLQKLRLRFTSFPDLCQGISGSLTHNQNLIHLDLKGSDIGD-DGVKSLCEALKHPDCKLQ
SLS---------------------LESCDLTTVCCLNISKALVRSQSLLSLNLSTNNLLD
DGVKLLCEGLMHPKCNLEKLSLESCGLTVACCEDLSLVLISNKRLTHLCLADNMLGDGGV

KFM---
-------------SEALKHPQCILQSLVLRRCHFTSLSSESLSASLLHNKSLMHLDLGSN
CLQDDGVKLLCDAFRHPSCSLQDLELMGCVLTSACCLDLASAILNNPNLQSLDLGNNDLR
DDGVKFLFEALRHPNCNIQRLGLEHCGLTSLCCQDLSSTLSSNQGLIKISLTLNTLQCEE
IMKLSEVLRSTECKLQVLGLCKEALDEEAQKLLEAVASSNLRLAVKQDCNDHEEEDGSWW
RCF---

#NLRP14_dog "gi|73988784|ref|XP_542482.2| PREDICTED: similar to NACHT, LRR and PYD containing protein 14 [Canis familiaris]"

-------------------MTDGSSSSFFPDFGLLLYLEELNKEELNKFKSFLKNETVEP
RSCQIP-------WVEVKKAKREDLANLMKKYYPGEQAWDVALKIFGKMNLKDLCERATA
EINWTARTMVTEGARTQEEQDDQEAVQ---------------------------------
--SDGTEYRIQIKE
KFRIMWDKKCLFGGPEDFHPGIAQEDRELLEHLFDVDVKTGEQPQ-TVVLQGAAGVGKTS
LVRKAIVDWAEGNLYQQKFSYVFYLNAREIN-QLRERSFVQMISKDWPSTEGPIERIMTQ
PSSLLFIIDSFDELNFAFEE----PDFVLCEDWTQVHPVSFLMSSLLRKVMLPESFLLVT
TRLTACKKLKPLLKNQHSVELLGMSKDARKEYIYQFFEDKK---------RASQVFSSLR
SNEMLFSMCKVPLVCWAICTCLEQQIEMGGDVSLTCKTTTALFTRYISSLFPPVDGN--C
PSLPNQTQLKSLCHLAAKGVWT-MTSVFYREDLRKHGLTKSDVSIFLDMNILQKDTE-YE
NCYVFTHLHVQEFLAAMFYMLRDNWETRNNLFDS-------FEDLKLLLESKSS-KDPHL
MQMKCFLFGLLNEDLLKQLETTLKCRLSLEIKGKILQWLE-ILGNI--------------
-----------KCFPAELEFLELFLCLYETQDEAFISQAMRSFQ----------------
-KVVIDVCGKVHLLVSS-FCLKHCQCLQTIKLSVTVVFEKTLNSSPPAEMCLEF------
-----SELIIIHCWQDLCSVLHTNEHLRELDLCHSNLDELAMKTFYQELRHP------NC
KLQRLLMRFLSFPGGCQDIASSLTHNQNLMHLDLKGSDIGD-DGVKSLCEALKHPECKLQ
NLS---------------------LESCGLTTLCCLNISKALIRSQSLRFLNLSTNHLLD
DGVKLLCEALGHPKCHLERLSLESCGLTVAGCEDLSLALISNKRLTHLCLADNILGDDGV
KLV---
-------------NDALKHPQCKLQSLVLRRCHFTSLSSEHLSSALLCNKSLIHLDLGSN
WIQDDGIKLLCDAFRHPSCNLQDLELMGCVLTSMCCLDLASAILNNSHLQNLDLGHNDLR
DDGVKILCEALRHPNCNIQRLGLEYCGLTSLCCQDLSYTLRSNQNLIKINLKQNTLGYEG
MMKLCEVLKSPECKLKVLG---

#NLRP14_chimpanzee "gi|114635946|ref|XP_521822.2| PREDICTED: NACHT, LRR and PYD containing protein 14 [Pan troglodytes]"

-------------------MADSSSSSFFPDFGLLLYLEELNKEELNTFKLFLK-ETMEP
EHGLTP-------WTEVKKARREDLANLMKKYYPGEKAWSVSLKIFGKMNLKDLCERAKE
EINWSAQTIGPDDAKAGETQEDQEAVL---------------------------------
--GDGTEYRIRIKE
KFCITWDKKSLAGKPEDFHHGIAEKDRKLLEHLFDVDVKTGEQPQ-IVVLQGAAGVGKTT
LVRKAVLDWAEGNLYQQRFKYVFYLNGREIN-KLKERSFAQLISKDWPSTEGPIEEIISQ
PSSLLFIIDSFDELNFAFEE----PEFALCEDCTQEHPVSFLMSSLLRKVMLPEASLLVT
TRLTTSKRLKQLLKNHHYVELLGMSEDAREEYIYQFFEDKR---------WAMKVFSSLK
SNEMVFSMCQVPLVCWATCTCLKQQMEKGGDVTLTCQTTTALFTCYISSLFTPVDGG--S
PSLPNQAQLRRLCQVAAKGIWT-MTYVFYRENLRRLGLTKSDVSSFMDSNIIQKDAE-YE
NCYVFTHLHVQEFFAAMFYMLKGSWEAGNPSCQP-------FEDLKSLLQSTSY-KDPHL
TQMKCFLFGLLNEDRVKQLERTFNCKMSLKIKSKLLQCME-VLGKS--------------
-----------DYSPSQLGFLELFHCLYETQDKAFISQAMRCFP----------------
-KVAINICEKIHLLVSS-FCLKHCRCLRTIRLSVTVVFEKKILKTSLPANTWD-------
------GDRITHCWQDLCSVLHTNEHLRELDLCHSNLDKSAMNILHHELRHP------NC

```
KLQKLLLKFITFPDGCQDISTSLIHNKNLMHLDLKGSDIGD-NGVKSLCEALKHPECKLQ
TLR---------------------LESCNLTVFCCLNISNALIRSQSLIFLNLSTNNLLD
DGVQLLCEALRHPKCYLERLSLESCGLTEAGCEYLSLALISNKRLTHLCLADNVLGDGGV
KLM---------------------------------------------------------
-------------SDALQHAQCTLKSLVLRRCHFTSLSSEYLSTSLLHNKSLTHLDLGSN
WLQDNGVKLLCDVFRHPSCNLQDLELMGCVLTNACCLDLASVILNNPNLRSLDLGNNDLQ
DDGVKILCDALRYPNCNIQRLGLEYCGLTSLCCQDLSSALICNKRLIKMNLTQNTLGYEG
IVKLYKVLKSPKCKLQVLGLCKEAFDEEAQKLLEAVGVSNPHLIIKPDCNYHNEEDVSWW
RCF---------------------------------------------------------
------------------------------------------------------------
------------------------------------------------------------
-----------------

#NLRP14_human "gi|28827813|ref|NP_789792.1| NLR family, pyrin domain containing
14 [Homo sapiens]"
------------------------------------------------------------
------------------------------------------------------------
-------------------MADSSSSSFFPDFGLLLYLEELNKEELNTFKLFLK-ETMEP
EHGLTP-------WNEVKKARREDLANLMKKYYPGEKAWSVSLKIFGKMNLKDLCERAKE
EINWSAQTIGPDDAKAGETQEDQEAVL---------------------------------
------------------------------------------------GDGTEYRNRIKE
KFCITWDKKSLAGKPEDFHHGIAEKDRKLLEHLFDVDVKTGAQPQ-IVVLQGAAGVGKTT
LVRKAMLDWAEGSLYQQRFKYVFYLNGREIN-QLKERSFAQLISKDWPSTEGPIEEIMYQ
PSSLLFIIDSFDELNFAFEE----PEFALCEDWTQEHPVSFLMSSLLRKVMLPEASLLVT
TRLTTSKRLKQLLKNHHYVELLGMSEDAREEYIYQFFEDKR---------WAMKVFSSLK
SNEMLFSMCQVPLVCWAACTCLKQQMEKGGDVTLTCQTTTALFTCYISSLFTPVDGG--S
PSLPNQAQLRRLCQVAAKGIWT-MTYVFYRENLRRLGLTQSDVSSFMDSNIIQKDAE-YE
NCYVFTHLHVQEFFAAMFYMLKGSWEAGNPSCQP-------FEDLKSLLQSTSY-KDPHL
TQMKCFLFGLLNEDRVKQLERTFNCKMSLKIKSKLLQCME-VLGNS--------------
-----------DYSPSQLGFLELFHCLYETQDKAFISQAMRCFP----------------
-KVAINICEKIHLLVSS-FCLKHCRCLRTIRLSVTVVFEKKILKTSLPTNTWD-------
------GDRITHCWQDLCSVLHTNEHLRELDLYHSNLDKSAMNILHHELRHP------NC
KLQKLLLKFITFPDGCQDISTSLIHNKNLMHLDLKGSDIGD-NGVKSLCEALKHPECKLQ
TLR---------------------LESCNLTVFCCLNISNALIRSQSLIFLNLSTNNLLD
DGVQLLCEALRHPKCYLERLSLESCGLTEAGCEYLSLALISNKRLTHLCLADNVLGDGGV
KLM---------------------------------------------------------
-------------SDALQHAQCTLKSLVLRRCHFTSLSSEYLSTSLLHNKSLTHLDLGSN
WLQDNGVKLLCDVFRHPSCNLQDLELMGCVLTNACCLDLASVILNNPNLRSLDLGNNDLQ
DDGVKILCDALRYPNCNIQRLGLEYCGLTSLCCQDLSSALICNKRLIKMNLTQNTLGYEG
IVKLYKVLKSPKCKLQVLGLCKEAFDEEAQKLLEAVGVSNPHLIIKPDCNYHNEEDVSWW
WCF---------------------------------------------------------
------------------------------------------------------------
------------------------------------------------------------
-----------------

#NLRP14_mouse "gi|50872155|ref|NP_001002894.1| NLR family, pyrin domain
containing 14 [Mus musculus]"
------------------------------------------------------------
------------------------------------------------------------
------------------------------------------------------------
------------------------------------------------------------
---MKTEDDEMEYEASKEETVSEDKDF---------------------------------
------------------------------------------------DDGIDYRTVIKE
NIFTMWYKTSLHGEFATLNCVITPKDQNLLQHIFDEDIQTSEAPQ-TVVLQGAAGIGKTT
LLKKAVLEWADGNLYQQ-FTHVFYLNGKEIS-QVKEKSFAQLISKHWPSSEGPIEQVLSK
PSSLLFIIDSFDELDFSFEE----PQFALCKDWTQISPVSFLISSLLRKVMLPESYLLVA
TRSTAWKRLVPLLQKPQRVKLSGLSKNARMDYIHHLLKDKA---------WATSAIYSLR
MNWRLFHMCHVCHMCQMICAVLKGQVEKGGRVEETCKTSTALFTYYICSLFPRIPVG--C
VTLPNETLLRSLCKAAVEGIWT-MKHVLYQQNLRKHELTREDILLFLDAKVLQQDTE-YE
NCYMFTHLHVQEFFAALFYLLRENLEEQDYPSEP-------FENLYLLLESNHI-HDPHL
EQMKCFLFGLLNKDRVRQLEETFNLTISMEVREELLACLE-GLEKD--------------
```

```
-----------DSSLSQLRFQDLLHCIYETQDQEFITQALMYFQ----------------
-KIIVRVDEEPQLRIYS-FCLKHCHTLKTMRLTARADLKNMLD---TAEMCLEG------
-----AAVQVIHYWQDLFSVLHTNESLIEMDLYESRLDESLMKILNEELSHP------KC
KLQKLIFRAVDFLNGCQDFT-FLASNKKVTHLDLKETDLGV-NGLKTLCEALKCKGCKLR
VLR---------------------LASCDLNVARCQKLSNALQTNRSSVFLNLSLNNLSN
DGVKSLCEVLENPNSSLERLALASCGLTKAGCKVLSSALTKSKRLTHLCLSDNVLEDEGI
KLL---------------------------------------------------------
-------------SHTLKHPQCTLQSLVLRSCSFTPIGSEHLSTALLHNRSLVHLDLGQN
KLADNGVKLLCHSLQQPHCNLQELELMSCVLTSKACGDLASVLVNNSNLWSLDLGHNILD
DAGLNILCDALRNPNCHVQRLGLENCGLTPGCCQDLLGILSNNKSVIQMNLMKNALDHES
IKNLCKVLRSPTCKMEFLALDKKEILKKKIKKFLVDVRINNPHLVIGPECPNTESGCWWN
YF----------------------------------------------------------
------------------------------------------------------------
------------------------------------------------------------
-----------------

#NLRP14_rat
------------------------------------------------------------
------------------------------------------------------------
------------------------------------------------------------
-------------------------------------------------------QKEGD
GWSVRILTVKLKKFDHVCLWFSILTDL---------------------------------
------------------------------------------------NYGIDYRTIIKE
NFFIIWDKTSLLGESATLNCVTTHKDQKLLEHIFDVNIQTSKSPQ-TVVLYGAAGIGKTT
LLKKAVLEWADGNLYQQ-FTHVFYLSGREIS-QEKEKNFAQLISKHWPSSEGPIEQIL--
------------------------------------------------------------
------------------------------------------------------------
------------------------QVEKGGNVEMTCQTSTALFTYYICSLFPRIAGS--S
VTLPNETLLRSLCQAAVEGIWT-MKHVLYQQNLRKHELTREDLSLFLEANVLQQDTE-CE
NCYTFTHLQVQEYFAALFYLLRENPEEKDHPLEP-------FENVHLLLESNHF-HDPHL
EQMKCFLFGLLNKDRVQQLEETFNLTISRAVREELLVCLE-ALEKD--------------
-----------DSPLSQLRIQDLLHCIYETQDEEFITQALTYFQ----------------
-KIMVKVDEESKLLIYS-YCLKHCHSLQTVRLTARADLRNMLDP--TAEICLRS------
-----VSVVFISYVEDVWWTVNSKDNLIVCEHWCHWMYESFLLIS-EHLSHI------VF
SQMSLVFRAVCFLNSCYDFS-FLAFNNTVTHLDLKDTDLGD-NGVKTLCEALKYQGCKLR
VLR---------------------LESCDLNVTHCQNLSKALQSNRSLVFLNLSTNSLSN
DGVKSLCEVLENPNCPLERLALASCGLTKVGCEVLSSALTKTK-----------------
------------------------------------------------------------
--------------------------------------------------SLIHLDLGFN
KLADSGVKLLCQTLQQPNCNLQELE-----------------------------------
------------------------------------------------------------
------------------------------------------------------------
------------------------------------------------------------
------------------------------------------------------------
------------------------------------------------------------
-----------------

#NLRP9a_mouse "gi|115299743|ref|NP_001041684.1| NACHT, LRR and PYD containing
protein 9a isoform 1 [Mus musculus]"
------------------------------------------------------------
------------------------------------------------------------
-------------------------MMDSSGYGLLQYLQKLSDEEFQRFKEHLRKEPEKF
KLKPIS-------WTKIKNTSKEDLVMQLYTHYP-GKAWDMVLSLFLQVNREDLSTMAQT
ERRD--------------------------------------------------------
-------------------------------------------------KQTKYKEFMKN
TFQHIWTMETNTYIPDRSYHEFIEVQYRALQDIFDCESEP-----VTVVVSGSRGGGKTT
FLRKAMLDWASRNLLQNRFQYVFYFSVFSLN-NITELSLAELISSTLPESSETVDDILSD
PKRILFILDGFDYLKFDLE----LR-TNLCNDWRKKLPIQIVLSSLLQKIMLPECSLLLE
LGNASLSNIIPLLQYPREIIMSGFSEQTIEIYCVSFFNTQ----------TGVEIFKNLK
SIKPLFNLCRCPHLCWMICSTIKWQYERREVASRFGRTLGLLYTIFMVSAFKSTYAR--N
PSKQNRARIRTLCTLAVEGMWK-QVYVFDSDDLRRNGISESDKKVWLRMKFLQNQG----
```

SNIVFYHSTLQWYFAVLFYFLQY-KDTRHPVIGN---------LAQLLGE-IYAHKQNQW
FHTRILLFGMATEQVNSLLEPCFGCISSKEVRQEIIRYIKSLSQQE--------------
-----------CNEKLVVHPQNLFFCILDNQEERFVRQLMDRFE----------------
-EMTVDISDVDDMSATP-YCLHRAPKVKNLHLHIQKRVFLEIHDPEYGDLELFKL-----
-----DQKLLAKHWTTLCTFLCN---LHVLDLDSCHFNEKAIEVLCNCLPLTSLVPLTGF
KLHRLLCSFTTNFGDGLLFCTFLHLP-HLKYMNLYGTNLSN-DAVERLCSALKFSTCGVE
ELL---------------------LGKCDISSEACGIIAASLIN-SEVKHLSLVENPLKN
KGVMSLCEMLKDPSCVLESLMLSYCCLTFIACGHLYEALLSNEHLSLLDLGSNFLEDTGV
NLL---
-------------CEALKDPNCTLKELWLPGCYLTSECCEEISAVLTCNTNLKTLKLGNN
NIQDTGVKRLCEALCHPNCEMQCLGLDMCDFTSDCCEDLALVLTTCNTLKSLNLDWNAFD
HSGLEMLCKALNHKACNLEVLGLDKSLFSEESQTLLQAVEKKNKNLKVLHFPWLKEELEK
RGVRLVWNSKN---

#NLRP9b_mouse "gi|52145307|ref|NP_918947.2| NACHT, LRR and PYD containing protein 9b [Mus musculus]"

-------------------------MAGSSGYGLLKLLQKLSDEEFQRFKELLREEPEKF
KLKPIS-------WTKIENSSKESLVTLLNTHYP-GQAWNMMLSLFLQVNREDLSIMAQK
KKRH--
---KQTKYKKFMKT
TFERIWTLETNTHIPDRNYHLIVEVQYKALQEIFDSESEP-----VTAIVAGTTGEGKTT
FLRKAMLDWASGVLLQNRFQYVFFFSVFSLN-NTTELSLAELISSTLPESSETVDDILSD
PKRILFILDGFDYLKFDLE----LR-TNLCNDWRKRLPTQIVLSSLLQKIMLPGCSLLLE
LGQISVPKIRHLLKYPRVITMQGFSERSVEFYCMSFFDNQ----------RGIEVAENLR
NNE-VLHLCSNPYLCWMFCSCLKWQFDREEEGYFKAKTDAAFFTNFMVSAFKSTYAH--S
PSKQNRARLKTLCTLAVEGMWK-ELFVFDSEDLRRNGISESDKAVWLKMQFLQTHG----
NHTVFYHPTLQSYFAAMFYFLKQDKDICVPVIGS---------IPQLLGN-MYARGQTQW
LQLGTFLFGLINEQVAALLQPCFGFIQPIYVRQEIICYFKCLGQQE--------------
-----------CNEKLERS-QTLFSCLRDSQEERFVRQVVDLLE----------------
-EITVDISSSDVLSVTA-YALQKSSKLKKLHLHIQKTVFSEIYCPDHCKTRTSIG-----
-----KRRNTAEYWKTLCGIFCN---LYVLDLDSCQFNKRAIQDLCNSMSPTPTVPLTAF
KLQSLSCSFMADFGDGSLFHTLLQLP-HLKYLNLYGTYLSM-DVTEKLCAALRCSACRVE
ELL---------------------LGKCGISSKACGIIAISLIN-SKVKHLSLVENPLKN
KGVMSLCEMLKDPSCVLQSLMLSYCCLTFIACGHLYEALLSNKHLSLLDLGSNFLEDTGV
NLL---
-------------CEALKDPNCTLKELWLPGCFLTSQCCEEISAVLICNRNLKTLKLGNN
NIQDTGVRQLCEALSHPNCNLECLGLDLCEFTSDCCKDLALALTTCKTLNSLNLDWKTLD
HSGLVVLCEALNHKRCNLKMLGLDKSAFSEESQTLLQDVEKKNNNLNILHHPWFEAERNK
RGTRLVWNSRN---

#NLRP9c_mouse "gi|110815824|ref|NP_001036077.1| NLR family, pyrin domain containing 9C [Mus musculus]"

-------------------------MVDSSSYGLLQYFQKLSDEEFQRFKELLQKEQEKF
KLKPLS-------WTKIKNTSKEDLVTQLYTHYP-RQVWDMVLNLFLQVNRKDLSTMAQI
ERRD--
---KQNKYKEFMKN
LFQYIWTSETNTYMPDRSYNTIIDRQYKALLDIFDSESDP-----ATAVVLGTRGKGKTV
FLRKAMLDWASGVLLQNRFQYVFFFSVFSLN-NTTELSLAELISSKLPECSETLDDILSN
PKRILFVLDGFDYLKFDLE----LR-TNLCNDWRKRLPTQNVLSSLLQKIMLPECSLLLE

```
LGESSCSKIIPLLQNPREIIMSGLSEQSIYFYCVSFFKIQ----------LGVEVFKDLK
KNEPLFTLCSNPSMLWMICSSLMWGHYSREEVISSSESTSAIHTIFIMSAFKSIFGL--G
SSKYKRFKLKTLCTLAVEGMWK-QVYVFDSEDLRRNKISESDKTVWLKMKFLQIQG----
NNIMFYHSTLQWYFATLFYFLKQDKDTYHPVIGS---------LPQLLGE-IYAHKQNQW
THAQTFFFGIATKHVITLLKPCFGNISFKTIRQEIIRYLKSLSQPE--------------
-----------CNEKLVHP-KKLFFCLIENQEERFVSQVMNLFE----------------
-EITVDISDSDDLGAAE-YSLLRASKLKNLHLHIQKKVFSEIHDPEYGSLENFKL-----
-----DQKFSAINWTMLSILFCN---LHVLDLGSCHFNKKVIEVLCNSLSPTPNMPLTVF
KLQRLLCSFMTNFGDGSLFCTFLQIP-QLKYLNLYGTDLSN-DVVEMLCSALKCSTCRVE
ELL---------------------LGKCDISSEACGIMATFLIN-SKVKHLSLVENPLKN
KGVMFLCKMLKDPSCVLESLMLSYCCLTFIACGHLYEALLSNKHLSLLDLGSNFLEDIGV
NLL---------------------------------------------------------
-------------CEALKYPNCTLKELWLPGCYLTSECCEEISAVLTCNKNLKTLKLGNN
NIQDTGVKRLCEALCHPKCKVQCLGLDMCELSNDCCEDLALALITCNTLKSLNLDWNALH
HSGLVMLCEALNHKKCKLNMLGLDKSSFSEESQTFLQAVEKKNNNLNVLHFPWVEDELKK
RGVRLVWNSKN-------------------------------------------------
------------------------------------------------------------
------------------------------------------------------------
------------------------------------------------------------
-----------------

#NLRP9b_rat "gi|62639014|ref|XP_218248.3| PREDICTED: similar to NACHT, LRR and
PYD containing protein 9b [Rattus norvegicus]"
------------------------------------------------------------
------------------------------------------------------------
-------------------------MVDTSGYGLLKHLRKLSDGEFWSFKELLRKEPEKF
KLKPIS-------WMKIENASKEELVMLLNTHYP-KQAWDMALSLFLQVNREDLSIMAQK
KRRY--------------------------------------------------------
-------------------------------------------------KQTKYKKFMKT
TFQSIWTLESNICIPDRSYHLIVEHQYRKLQNIFDSESEP-----VTAVVTGPTGEGKTV
FLRKAMLDWASGILWQNRFQYVFFFSVLSLN-NTTELSLAELISSKLPESSETLNDILSD
PKKILFILDGFDYLKFDLE----LR-TNLCNDWRKILPTQIILSSLLQKIMLPESSLLLE
LGHISLPKIFPLLQYPRDITIQGFSERCLKTYFISFFNTE----------KGIEVFENLK
SNQ-MLKLCSNPYLCWMFCSCLKWQFDREEEAYFQAKTDSVFFTSFMVSAFKSAYAS--N
PPKQNRAQLKSLCTLAVEGMWK-QLFVFDSEDLRRNGISESDKAVWLRMKFLQNHD----
NHIVFYHPTLQLYFASMFYFLKQDKDTHVPVIGS---------IPQLLRK-IYARDHTQW
LQIGIFLFGLATEQVASLLKPYFGFIQHRDVRQEVMRYLKSLSQRE--------------
-----------CCEKLERP-QNLFACLRDNKEEKFVREVVDLFE----------------
-EITVDITNSHVLSIIA-NHLQKSSKLKKLHLHIQKRVFLEIHDPEYSDSETFTQ-----
-----DKKNAAEYWKKLCHIFVN---LHVLDLDSCNFNKQVIEELCNVLSPSPKIPLMAF
KLESLLCSFMTNFGDGSLFHTLLQLP-HLKYLNLYGTNLSN-SRIENLCSALRRSTCKVE
ELL---------------------LGKCDISSEACGIIATSLMN-SKVKHLSLVENPLKN
KGVMSLCEMLKDPSCVLETLMLSYCCLTFIACGHLYEALVSNKHLSLLDLGSNFLEDTGV
NLL---------------------------------------------------------
-------------CEALKDPNCILKELWLSGCFLTSECCEEISAVLTCNNNLKTLKLGNN
DIEDTGVKHLCEALSHPNCKLECLGLDLCKFTSDCCEDLASALTTCKTLNSLNLDWKTLE
HSGVVALCEALNHKKCNLKMLGLDKSAFSVESQTLLQAVEKKNNNLSILHYPWVEEERKK
RGVRLVWNSKN-------------------------------------------------
------------------------------------------------------------
------------------------------------------------------------
------------------------------------------------------------
-----------------

#NLRP9a_rat "gi|34854707|ref|XP_218250.2| PREDICTED: similar to NACHT, LRR and
PYD containing protein 9b [Rattus norvegicus]"
------------------------------------------------------------
------------------------------------------------------------
-------------------------MVDTSGYGLLKHLRKLSDGEFWSFKELLRKEPEKF
KLKPIS-------WMKIENASKEELVMLLNTHYP-KQAWDMALSLFLQVNREDLSIMAQK
KRRY--------------------------------------------------------
-------------------------------------------------KQTKYKKFMKT
```

TFQSIWTLESNICIPDRSYHLIVEHQYRKLQNIFDSESEP-----VTAVVTGPTGEGKTV
FLRKAMLDWASGILWQNRFQYVFFFSVLSLN-NTTELSLAELISSKLPESSETLNDILSD
PKKILFILDGFDYLKFDLE----LR-TNLCNDWRKILPTQIILSSLLQKIMLPESSLLLE
LGHISLPKIFPLLQYPRDITIQGFSERCLKTYFISFFNTE----------KGIEVFENLK
SNQ-MLKLCSNPYLCWMFCSCLKWQFDREEEAYFQAKTDSVFFTSFMVSAFKSAYAS--N
PPKQNRAQLKSLCTLAVEGMWK-QLFVFDSEDLRRNGISESDKAVWLRMKFLQNHD----
NHIVFYHPTLQLYFASMFYFLKQDKDTHVPVIGS---------IPQLLRK-IYARDHTQW
LQIGIFLFGLATEQVASLLKPYFGFIQHRDVRQEVMRYLKSLSQRE--------------
-----------CCEKLERP-QNLFACLRDNKEEKFVREVVDLFE---------------
-EITVDITNSHVLSIIA-NHLQKSSKLKKLHLHIQKRVFLEIHDPEYSDSETFTQ-----
-----DKKNAAEYWKKLCHIFVN---LHVLDLDSCNFNKQVIEELCNVLSPSPKIPLMAF
KLESLLCSFMTNFGDGSLFHTLLQLP-HLKYLNLYGTNLSN-SRIENLCSALRRSTCKVE
ELL---

#NLRP9_human "gi|33519450|ref|NP_789790.2| NLR family, pyrin domain containing 9 [Homo sapiens]"

-----------------------MAESFFSDFGLLWYLKELRKEEFWKFKELLKQPLEKF
ELKPIP-------WAELKKASKEDVAKLLDKHYPGKQAWEVTLNLFLQINRKDLWTKAQE
EMRN--
---KLNPYRKHMKE
TFQLIWEKETCLHVPEHFYKETMKNEYKELNDAYTAAARR-----HTVVLEGPDGIGKTT
LLRKVMLDWAEGNLWKDRFTFVFFLNVCEMN-GIAETSLLELLSRDWPESSEKIEDIFSQ
PERILFIMDGFEQLKFNLQ----LK-ADLSDDWRQRQPMPIILSSLLQKKMLPESSLLIA
LGKLAMQKHYFMLRHPKLIKLLGFSESEKKSYFSYFFGEKS---------KALKVFNFVR
DNGPLFILCHNPFTCWLVCTCVKQRLERGEDLEINSQNTTYLYASFLTTVFKAGSQS--F
PPKVNRARLKSLCALAAEGIWT-YTFVFSHGDLRRNGLSESEGVMWVGMRLLQRRG----
DCFAFMHLCIQEFCAAMFYLLKRPKDDPNPAIGS---------ITQLVRA-SVVQPQTLL
TQVGIFMFGISTEEIVSMLETSFGFPLSKDLKQEITQCLESLSQCE--------------
-----------A-DREAIAFQELFIGLFETQEKEFVTKVMNFFE----------------
-EVFIYIGNIEHLVIAS-FCLKHCQHLTTLRMCVEN---------IFPDDSGCIS-----
-----DYNEKLVYWRELCSMFITNKNFQILDMENTSLDDPSLAILCKALAQP-----VCK
LRKLIFTS-VYFGHDSELFKAVLHNP-HLKLLSLYGTSLSQ-SDIRHLCETLKHPMCKIE
ELI---------------------LGKCDISSEVCEDIASVLACNSKLKHLSLVENPLRD
EGMTLLCEALKHSHCALERLMLMYCCLTSVSCDSISEVLLCSKSLSLLDLGSNALEDNGV
ASL---
-------------CAALKHPGCSIRELWLMGCFLTSDSCKDIAAVLICNGKLKTLKLGHN
EIGDTGVRQLCAALQHPHCKLECLGLQTCPITRACCDDIAAALIACKTLRSLNLDWIALD
ADAVVVLCEALSHPDCALQMLGLHKSGFDEETQKILMSVEEKIPHLTISHGPWIDEEYKI
RGVLL---

#NLRP9_chimpanzee "ENSPTRT00000021478 peptide:ENSPTRP00000019820 pep:NOVEL_protein_coding"

-----------------------MAESFFSDFGLLWYLKELRKEEFWKFKELLKQPLEKF

```
ELKPIP-------WAELKKASKEDVAKLLDKHYPGKQAWEVTLNLFLQINRKDLWTKAQE
EMRN--------------------------------------------------------
-------------------------------------------------KLNPYRKHMKE
TFQLIWEKETCLHVPEHFYKETMKNEYKELNDAYTAAARR-----HTVVLEGPDGIGKTT
LLRKVMLDWAEGNLWKDRFTFVFFLNVYEMN-SIAETSLLELLSRDWPESSEKIEDIFSQ
PERILFIMDGFEQLKFNLQ----LK-ADLSDDWRQRQPMPIILSSLLQKKMLPESSLLIA
LGKLAMQKHYFMLRHPKLIKLLGFSESEKKSYFSYFFGEKS---------KALKVFNFVR
DNGPLFILCHNPFTCWLVCTCVKQRLERGEDLEINSQNTSYLYASFLTTVFKAGSQS--F
PPKVNRARLKSLCALAAEGIWT-YTFVFSRGDLRRNGLSESEGVMWVGMRLLQRRG----
DCFAFMHLCIQEFCAAMFYLLKRPKDDPNPAIGS---------ITQLVRA-SVVQPQTLL
TQVGIFMFGISTEEIVSMLETSFGFPLSKDLKQEITQCLESLSQCE--------------
-----------A-DREAIAFQELFIGLFETQEKEFVTKVMNFFE----------------
-EVFIYIGNIEHLVIAS-FCLKHCQHLTTLRMCVEN---------IFPDDSGCIS-----
-----DYNEKLVYWRELCSMFITNKNFQILDMENTSLDDPSLAILCKALAHP-----VCK
LR-KLFTS-VYFGHDSELFKAVLHNP-HLKLLSLYGTSLSQ-SDIRHLCETLKHPMCKIE
ELI---------------------LGKCDISSEACEDIASVLACNSKLKHLSLVENPLRD
EGMTLLCEALKHPHCALERLMLMG------------------------------------
------------------------------------------------------------
-------------------------------CFLTSDSCKDIAAVLICNEKLKTLKLGHN
EIGDTGVRQLCAALQHPHCKLECLGLQTCPITRACCGDLAAALIACKTLRSLNLDWIALD
VDAVVVLCEALSHPDCALQMLGLHKSGFDEETQKILMSVEEKIPHLTISHGPWIDEEYKI
RGVLL-------------------------------------------------------
------------------------------------------------------------
------------------------------------------------------------
------------------------------------------------------------
-----------------
```

#NLRP10_chimpanzee "ENSPTRT00000006299 peptide:ENSPTRP00000005809 pep:KNOWN_protein_coding"

```
------------------------------------------------------------
------------------------------------------------------------
---------------------MAMAKARNPREALLWALSDLEENDFKKLKFYLRDMTLSE
GQPPLARGE-------LEGLIPVDLAELLISKYGEKEAVKVVLKGLKVMNLLELVQLS-H
ICLH--------------------------------------------------------
------------------DYREVYREHVR-CLEEWQEA-----GVNGRYNQVLLVAKPSS
ESPESLA-------CPIPEQE----LDSVTVEALFDSGEKPSLAPSLVVLQGSAGTGKTT
LARKMVLDWATGTLYPGRFDYVFYVSCKEVVLLLES-KLEQLLFWCCGDNQAPVTEILRQ
PERLLFILDGFDELQRPFEE----KLKKR-----GLSPKESLLHLLIRRHTLPTCSLLIT
TRPLALRNLEPLLKQARHVHILGFSEEERARYFSSYFTDEK---------QADRAFDIVQ
KNDTLYKACQVPGICWVVCSWLQGQMERGKVVLETP-RNSTDIFMAYVSTFLPPDDDGGC
SELSRHRVLRSLCSLAAEGIQH-QRFLFEEAELRKHNLDGP-RLAAFLSSNDYQLGLAIK
KFYSFRHISFQDFFHAMSYLVKEDQSRL-GKESRR--------EVQRLLEVKEQEGNDEM
TLTMQFLLDISKKDSFSNLELKFCFRISPCLAQDLKHFKEQMES----------------
--------------MKHNRTWDLEFSLYEAKIKNLVKGIQMNNV----------------
SFKIKHSNEKKSQSQNLFSVKSSLSHGPKEEQKCPSVHGQKEGKDNIAGTPKEAS-----
--------------------TGKGRGTEE------TPKNTYI------------------
------------------------------------------------------------
------------------------------------------------------------
------------------------------------------------------------
------------------------------------------------------------
------------------------------------------------------------
------------------------------------------------------------
------------------------------------------------------------
------------------------------------------------------------
------------------------------------------------------------
------------------------------------------------------------
------------------------------------------------------------
-----------------
```

#NLRP10_human "gi|28827807|ref|NP_789791.1| NLR family, pyrin domain containing 10 [Homo sapiens]"

```
------------------------------------------------------------
------------------------------------------------------------
---------------------MAMAKARKPREALLWALSDLEENDFKKLKFYLRDMTLSE
GQPPLARGE-------LEGLIPVDLAELLISKYGEKEAVKVVLKGLKVMNLLELVDQLSH
ICLH--------------------------------------------------------
------------------DYREVYREHVR-CLEEWQEA-----GVNGRYNQVLLVAKPSS
ESPESLA-------CPFPEQE----LESVTVEALFDSGEKPSLAPSLVVLQGSAGTGKTT
LARKMVLDWATGTLYPGRFDYVFYVSCKEVVLLLES-KLEQLLFWCCGDNQAPVTEILRQ
PERLLFILDGFDELQRPFEE----KLKKR-----GLSPKESLLHLLIRRHTLPTCSLLIT
TRPLALRNLEPLLKQARHVHILGFSEEERARYFSSYFTDEK---------QADRAFDIVQ
KNDILYKACQVPGICWVVCSWLQGQMERGKVVLETP-RNSTDIFMAYVSTFLPPDDDGGC
SELSRHRVLRSLCSLAAEGIQH-QRFLFEEAELRKHNLDGP-RLAAFLSSNDYQLGLAIK
KFYSFRHISFQDFFHAMSYLVKEDQSRL-GKESRR--------EVQRLLEVKEQEGNDEM
TLTMQFLLDISKKDSFSNLELKFCFRISPCLAQDLKHFKEQMES----------------
--------------MKHNRTWDLEFSLYEAKIKNLVKGIQMNNV----------------
SFKIKHSNEKKSQSQNLFSVKSSLSHGPKEEQKCPSVHGQKEGKDNIAGTQKEAS-----
--------------------TGKGRGTEE------TPKNTYI------------------
------------------------------------------------------------
------------------------------------------------------------
------------------------------------------------------------
------------------------------------------------------------
------------------------------------------------------------
------------------------------------------------------------
------------------------------------------------------------
------------------------------------------------------------
------------------------------------------------------------
------------------------------------------------------------
------------------------------------------------------------
-----------------

#NLRP10_dog "gi|73988806|ref|XP_854074.1| PREDICTED: similar to NACHT, LRR and
PYD containing protein 10 [Canis familiaris]"
------------------------------------------------------------
------------------------------------------------------------
-----------------------MALTRNPREALLWALSDLEEKNFKLFKFHLRDRSLLE
GRLQLARGE-------LEGLSPVDLASRLISLYGALEAVKVVVRVLRVMNLLEPVDQLSH
ICLN--------------------------------------------------------
------------------DYREIYREHVRCCLEERQEE-----SICGSHSQLLLVATSSP
GSPESSA-------CPVLEQE----LDSVLVETLFDPGEKSYQAPPTVVLQGSAGTGKTT
LARKMVLDWATGTLYPGRFDYVFYVSCREVVLLQEG-VLDQLLFWCCGDNQAPVREMLRE
AERLLFILDGFDELQRPFVR----GLKSL-----RLSPMEKVLHCLIRREVLSTCSLLIT
TRPLALQNLNPLLKQSHHIHILGFSEDERRRYFSSYFMDEE---------QARNAFDIVR
GNDVLYKSCQIPGICWVICSWIKEQMEKGREISETP-SNGTDIFMAYVSTFLPPSDNEGC
SKLTRHRVLKGLCSLAAEGIQH-QRFMFEEADLRKHNLDGT-RLAAFLSSMDYQEGLDIK
KFYIFRHISFQEFFHAMSYLVKEDQSQL-GKESRK--------EVKRLLDEERQAENEEM
TLSMHFLLDILKKETSSNFELKFSLKISPLVKQDLKHFKEQMKS----------------
--------------IKHKGAWDLEFSLNQSKIRNLVKGVQISDV----------------
SLKMQRSNKKQPHDRNSFSVKTSLINRWKEKEKCPFVD-----EDNIEKTQMEAS-----
--------------------NGKGRKMEK------QEMLSVGAVGREVEEWD------SR
AEGSGRLESQGQEDGGRQSLKKMER-----------------------------------
------------------------------------------------------------
------------------------------------------------------------
------------------------------------------------------------
------------------------------------------------------------
------------------------------------------------------------
------------------------------------------------------------
------------------------------------------------------------
------------------------------------------------------------
------------------------------------------------------------
------------------------------------------------------------
-----------------
```

#NLRP10_mouse "gi|30425308|ref|NP_780741.1| NACHT, LRR and PYD containing protein 10 [Mus musculus]"

---------------------MALARANSPQEALLWALNDLEENSFKTLKFHLRDVTQFH
----LARGE-------LESLSQVDLASKLISMYGAQEAVRVVSRSLLAMNLMELVDYLNQ
VCLN--
------------------DYREIYREHVR-CLEERQDW-----GVNSSHNKLLLMATSSS
GGRRSPS-------CSDLEQE----LDPVDVETLFAPEAESYSTPPIVVMQGSAGTGKTT
LVKKLVQDWSKGKLYPGQFDYVFYVSCREVVLLPKC-DLPNLICWCCGDDQAPVTEILRQ
PGRLLFILDGYDELQK-------------------SSRAECVLHILMRRREVP-CSLLIT
TRPPALQSLEPMLGERRHVLVLGFSEEERETYFSSCFTDKE---------QLKNALEFVQ
NNAVLYKACQVPGICWVVCSWLKKKMARGQEVSETP-SNSTDIFTAYVSTFLPTDGNGDS
SELTRHKVLKSLCSLAAEGMRH-QRLLFEEEVLRKHGLDGP-SLTAFLNCIDYRAGLGIK
KFYSFRHISFQEFFYAMSFLVKEDQSQQ-GEATHK--------EVAKLVD---PENHEEV
TLSLQFLFDMLKTEGTLSLGLKFCFRIAPSVRQDLKHFKEQIEA----------------
--------------IKYKRSWDLEFSLYDSKIKKLTQGIQMKDV----------------
ILNVQHLDEKKSDKKKSVSVTSSFSSGKVQSPFLGNDKSTRKQKKASNGKSRGAE-----
--------------------EPAPGVRNR------RLASREKGHMEMNDKED------GG
VEEQEDEEGQTLKKDGEMIDKMNG------------------------------------

#NLRP10_rat "gi|109459216|ref|XP_219247.3| PREDICTED: similar to NACHT, LRR and PYD containing protein 10 [Rattus norvegicus]"

---------------------MALARANSPQEALLWALNDLEENSFKTLKFHLRDVTQFH
----LARGE-------LEGLSQVDLASKLISMYGAQEAVRVVGRSLQAMNLMELVDYLNQ
VCLN--
------------------DYREIYREHVL-CLEERQDW-----GVNSSHNKLLLVATSSS
GSLRSPS-------CSDLEQE----LDPVHVETLFAPEAESYSTPPIVVMQGSAGTGKTT
LVKKLVQDWAKGKLYPGQFDYVFYVSCREVVLLPKC-DLPNLICWCCGDDQAPVTEILRQ
PERLLFILDGYDELQK-------------------SSRAERVLHILMRRREVP-CSLLIT
TRPPALQSLEPMLGERRHVHVLGFSEEERETYFCSYFMDKE---------QLRNALEFVQ
NNAVLYKACQVPGICWVVCSWLKGKMARGQEVSETP-SNSTDIFTAYVSTFLPTDGNGDS
SELTRHKVLRGLCSLAAEGMRH-QRLLFEEDVLRKHSLDGP-SLTAFLNCTDYREGLGIK
KLYSFCHISFQEFFYAMSFLVKEDQGQL-GEATHK--------EVAKLVD---QENYEEV
TLSLQFLFDMLKTDNTLSLGLKFCFKIAPSVRQDLKHFKEQVVA----------------
--------------IKYNRSWDLEFSLYDSKIKKLKQGIQMKDV----------------
IFNVQHSDEKKSGKKKPFSVTSRFGKG----PILETGKSTRKQKKASNGKSRETE-----
--------------------EPPAQDGGK------SRLASRGKGNKEINDKD------GG
VYGQEDGEGQEVKKGGEILDEMNG------------------------------------

#NLRP12_rat "gi|109458044|ref|XP_218181.3| PREDICTED: similar to PYRIN-containing APAF1-like protein 7 isoform 2 [Rattus norvegicus]"

---------------------MLPATAKDALHRLSTYLEELEAGELKKFKLYLG-IAEEL
GQDKIPRG-------RMEMAGPLEMAQLMVAHMGTKEAWLLALSTFERIHRKDLWERGQG
EDLVRVTP--SNGLCS
LESQSTCLSDVSPDAPRKDPQITYKDYVRRKFRLMEDRNARLGECVNLSHRYTRLLLVKE
HSNPIWAQQKLLETGWEHSRTRGHQASPIQMETLFEPDEERPEPPRTVVLQGAAGMGKSM
LTHKVMLDWADGRLFQDQFDYVFYISCRELNRSHTQCSVHDLLSSCWPEHGAPLEDLIRA
PDRLLFIIDGFHELHPSFHD----VQGPWCHCWEEKRPTELLLGSLIRRLLLPQLSLLIT
TRPCALEKLHGLLEHPRHVEILGFSEAEREEYFYRYFHNTG---------QASQVFSFMR
DYEPLFTMCFVPMVSWVVCTCLKQQLESGELLRQTSRTTTAVYMFYLLSLMQPKPGT---
PTFKVPANQRGLVSLAAEGLWN-QKILFEEEDLGKHGLDGAEVSTFLNVNIFQKGIK-CE
KFYSFIHLSFQEFFTAMYCALHGRE------------------AVRRALAEYGFSERNFL
AHTVRFLFGLLNEEMRCYLERNLGWTISPQVKEEALAWIQNKARS---------------
-----------EGSTLQHGSLELLSCLYEIQEEDFIQQALSHFQ----------------
-VVVVRNLSTKMEHVVCSFCARYCRGTEVLHLYGSAYSTGAEDGPPEPPGAQTQSTHS--
---QERNILPDIYSAYLSATICTNSNLIELALYRNALGSQGVRLLCQGLRHA-----NCK
LQNLRLKRCHISGSACQDLAAAIIANRNLIRLDLSGNSIGV-LGLELLCEGLQHPMCRLQ
MIQ---------------------LRKCLLEAAAGRALASVLSNNSHLVELDLTGNPLED
LGLKLLCQGLRHPVCRLRTLWLKICHLGQASCEDLASTLKMNQSLMELDLGLNDLGDSGA
LLL---
-------------CEGLRHPDCKLQTLRLGICRLGSDACAGVASVLQVNTCLRELDLSFN
DLGDRGLWLLGEGLRHQTCRLQKLWLDSCGLTSKACEDLSSVLGISQTLNELYLTNNALG
DTGVRLLCKRLRHPGCKLRVLWLFGMDLNKVTHRRMAALRVTKPYLDIGC----------

#NLRP12_chimpanzee "gi|114678929|ref|XP_524387.2| PREDICTED: PYRIN-containing APAF1-like protein 7 isoform 5 [Pan troglodytes]"

---------------------MLRTAGRDGLCRLSTNLEELEAVELKKFKLYLG-TATEL
GEGKIPWG-------RMKKAGPLEMAQLLITHFGPEEAWRLALSTFERINRKDLWERGQR
EDLVRDTP--PGGPSS
LGNQSTCLLEVSLVTPRKDPQETYRDYVRRKFRLMEDRNARLGECVNLSHRYTRLLLVKE
HSNPMQAQQQLLDTGRGHARTVGHQASPIKIETLFEPDEERPEPPRTVVMQGAAGIGKSM
LAHKVMLDWADGKLFQGRFDYLFYINCREMNQSATECSMQDLISGCWPEPSAPLQELIRV
PERLLFIIDGFDELKPSFHD----AQGPWCLCWEEKRPTELLLNSLIRKKLLPELSLLIT
TRPTALEKLHRLLEHPRHVEILGFSEAERKEYFYKYFHNAE---------QAGQVFNYVR
DNEPLFTMCFVPLVCWVVCTCLQQQLEGGGLLRQTSRTTTAVYMLYLLSLMQPKPGA---
PRLQPPPNQRGLCSLAADGIWN-QKILFEEQDLRKHGLDGEDVSAFLNMNIFQKDMN-CE
RYYSFIHLSFQEFFAAMYYILDEGERGAGPD-----------QDVTRLLTEYAFSERSFL
ALTSRFLFGLLNEETRSHLEKSLCWKVSPHIKMDLLQWIQSKAQS---------------
-----------DGSTLQQGSLEFFSCLYEIQK-EFTQQALSHFQ----------------
-VIVVSNIASKMEHMVSSFCLKHCRSAQVLHLYGATYSADGEDRARCSAGTHTLLVQL--
---PERTVLLDAYSEHLAAALCTNPNLIELSLYRNALGSRGVKLLCQGLRHP-----NCK
LQNLRLKRCRISSSACEDLSAALIANKNLTRMDLSGNGVGF-PGMMLLCEGLRHPQCRLQ
MIQ---------------------LRKCQLESGACQEMASVLGTNPHLVELDLTGNALED
LGLRLLCQGLRHPVCRLRTLWLKICRLTAAACDELASTLSVNQSLRELDLSLNELGDLGV
LLL---
-------------CEGLRHPTCKLQTLRLGICRLGSAACEGLSVVLQANHNLRELDLSFN
DLGDWGLWLLAEGLQHPACRLQKLWLDSCGLTAKACKNLYFTLGINQTLTDLYLTNNALG

```
DTGVRLLCKRLSHPGCKLRVLWLFGMDLNKMTHSRLAALRVTKPYLDIGC----------
------------------------------------------------------------
------------------------------------------------------------
------------------------------------------------------------
------------------------------------------------------------
-----------------

#NLRP12_dog "ENSCAFT00000004310 peptide:ENSCAFP00000003989
pep:NOVEL_protein_coding"
------------------------------------------------------------
------------------------------------------------------------
---------------------MPRAPPSSGLCRLSAYLEELEAVELKKFKLFLG-TEAEA
G--RIPWG-------RLEAAGPLDTAQLLVAHCGPHAAWPLALGLFQRINRRDLWEKGRR
EEPVRDTP----------------------------------------------SGDPSS
PGSQSACSLEVFPGALRRDPRETYRDYVRRKFRLMEDRNARLGECVNLSHRYTRLLLVKE
HSNPMWAQQKLLDTGWGQARTVGHQASFIQMETLFEPDEERPEPPRTVVLQGAAGMGKSM
LAHKVMLDWADGRLFQDRFDYLFYINCRKMNQSTAEQSAQDLISSCWPEPSVPLQELVRV
PERLLFIIDGFHELKPSFHD----PQGPWCLCWEEKRPTELLLSSLIRKKLLPELSVLIT
IRPTALEKLHRLLEHPRHVEILGFSEAERKEYFYKYFHNAE---------QAGQVFNFIR
DNEPLFTLCFVPMVCWVVCTCLKQQLEDGGLLRQTSRTTTAVYMLYLLSLMQPKPGS---
PILQSPPNQRGLCSLAADGLWN-QKILFEEQDLRKHGLDGADVSSFLNMNIFQKDIN-CE
KFYSFIHLSFQEFFAAMYYILDPGESRSSPE-----------HNVTRLLAEYEFSERSFL
ALTVRFLFGLLNEETRSYLEKSLCWKVSPHVKVELLEWIQRKAQS---------------
-----------EGSTLQQGSLELFSCLYEIQEEDFIQQALSPFQ----------------
-VVVVNNIATKMEHMISSFCVKNCRSALVLHLHGAAYSPDEDDGGRWASGPQMLPTQI--
---PEKNVLPDAYSKQLAAALSTNPNLTELVLYRSALGSRGVRLLCQGLRHP-----SCK
LQNLSLKRCCVASSACQDLAAALMANQNLRRMDLSSNRLGL-PGLRALCKGLRHPRCKLQ
VIQ---------------------LRKCQLEAEACQEIASVLSTSRHLEELDLTGNALED
LGLKLLCQGLRHPVCRLQILWLKICHLTAAACEDLASTLGVNQSLIELDLSLNDLGDPGV
LLL---------------------------------------------------------
-------------CEGLRHPQCRLQALRLGICRLSSAACKGLCTVLQVNPCLRDLDLSFN
DLGDAGVWPLCEGLRHPTCRLQKLWLDSCGLTAKACEDLSSALGVSQTLRELYLTNNALG
NAGVRLLCKGLSHPGCKLQVLWLFGMELNKMTHRRLAALRVVKPQLDIGC----------
------------------------------------------------------------
------------------------------------------------------------
------------------------------------------------------------
------------------------------------------------------------
-----------------

#NLRP12_human "gi|21955154|ref|NP_653288.1| NLR family, pyrin domain containing
12 isoform 2 [Homo sapiens]"
------------------------------------------------------------
------------------------------------------------------------
---------------------MLRTAGRDGLCRLSTYLEELEAVELKKFKLYLG-TATEL
GEGKIPWG-------SMEKAGPLEMAQLLITHFGPEEAWRLALSTFERINRKDLWERGQR
EDLVRDTP----------------------------------------------PGGPSS
LGNQSTCLLEVSLVTPRKDPQETYRDYVRRKFRLMEDRNARLGECVNLSHRYTRLLLVKE
HSNPMQVQQQLLDTGRGHARTVGHQASPIKIETLFEPDEERPEPPRTVVMQGAAGIGKSM
LAHKVMLDWADGKLFQGRFDYLFYINCREMNQSATECSMQDLIFSCWPEPSAPLQELIRV
PERLLFIIDGFDELKPSFHD----PQGPWCLCWEEKRPTELLLNSLIRKKLLPELSLLIT
TRPTALEKLHRLLEHPRHVEILGFSEAERKEYFYKYFHNAE---------QAGQVFNYVR
DNEPLFTMCFVPLVCWVVCTCLQQQLEGGGLLRQTSRTTTAVYMLYLLSLMQPKPGA---
PRLQPPPNQRGLCSLAADGLWN-QKILFEEQDLRKHGLDGEDVSAFLNMNIFQKDIN-CE
RYYSFIHLSFQEFFAAMYYILDEGEGGAGPD-----------QDVTRLLTEYAFSERSFL
ALTSRFLFGLLNEETRSHLEKSLCWKVSPHIKMDLLQWIQSKAQS---------------
-----------DGSTLQQGSLEFFSCLYEIQEEEFIQQALSHFQ----------------
-VIVVSNIASKMEHMVSSFCLKRCRSAQVLHLYGATYSADGEDRARCSAGAHTLLVQL--
---PERTVLLDAYSEHLAAALCTNPNLIELSLYRNALGSRGVKLLCQGLRHP-----NCK
LQNLRLKRCRISSSACEDLSAALIANKNLTRMDLSGNGVGF-PGMMLLCEGLRHPQCRLQ
MIQ---------------------LRKCQLESGACQEMASVLGTNPHLVELDLTGNALED
LGLRLLCQGLRHPVCRLRTLWLKICRLTAAACDELASTLSVNQSLRELDLSLNELGDLGV
```

LLL---
-------------CEGLRHPTCKLQTLRLGICRLGSAACEGLSVVLQANHNLRELDLSFN
DLGDWGLWLLAEGLQHPACRLQKLWLDSCGLTAKACENLYFTLGINQTLTDLYLTNNALG
DTGVRLLCKRLSHPGCKLRVLWLFGMDLNKMTHSRLAALRVTKPYLDIGC----------

#NLRP12_mouse

---------------------MLPSTARDGLYRLSTYLEELEAGELKKFKLFLG-IAEDL
SQDKIPWG-------RMEKAGPLEMAQLMVAHMGTREAWLLALSTFQRIHRKDLWERGQG
EDLVRVTP--NNGLCL
FESQSACPLDVSPNAPRKDLQTTYKDYVRRKFQLMEDRNARLGECVNLSNRYTRLLLVKE
HSNPIWTQQKFVDVEWERSRTRRHQTSPIQMETLFEPDEERPEPPHTVVLQGAAGMGKSM
LAHKVMLDWADGRLFQGRFDYVFYISCRELNRSHTQCSVQDLISSCWPERGISLEDLMQA
PDRLLFIIDGFDKLHPSFHD----AQGPWCLCWEEKQPTEVLLGSLIRRLLLPQVSLLIT
TRPCALEKLHGLLEHPRHVEILGFSEEARKEYFYRYFHNTG---------QASRVLSFLM
DYEPLFTMCFVPMVSWVVCTCLKQQLESGELLRQTPRTTTAVYMFYLLSLMQPKPGT---
PTFKVPANQRGLVSLAAEGLWN-QKILFDEQDLGKHGLDGADVSTFLNVNIFQKGIK-CE
KFYSFIHLSFQEFFAAMYCALNGRE------------------AVRRALAEYGFSERNFL
ALTVHFLFGLLNEEMRCYLERNLGWSISPQVKEEVLAWIQNKAGS---------------
-----------EGSTLQHGSLELLSCLYEVQEEDFIQQALSHFQ----------------
-VVVVRSISTKMEHMVCSFCARYCRSTEVLHLHGSAYSTGMEDDPPEPSGVQTQSTYL--
---QERNMLPDVYSAYLSAAVCTNSNLIELALYRNALGSQGVRLLCQGLRHA-----SCK
LQNLR---

#NLRP3_chicken_Q5F3J4

---MAGEESTILLEALEG
LTLEDFQEF-------KKKLPHTDIKGGWNIGRDELEKVTHPSSLISYMGDSYREGAAMD
IAIS--
----------LFEEMNQRDLAEKILDEKVKEYKQKYTEHVAREFLQYKEANSCLGENLSV
RDGYTNLTIARKSWDQHGDEPGDVSSDTVTTQTLLEPSKDGQVPPITVLVG-ASGMGKTM
TIRKVMMEWVEG-TLCTQFDYVFCIDCKELS-FSKEVSMVDLISKCCPQQRMPAGRILGN
PEKILFIFDSFEALGLPLAQ----PKDELSTDPTEAKPLETTLLSLLRRTVLPESSVLIA
TRPAALQSLGQCLEGKHYVEILGFSPAAREEYFHRYFGNDN---------KADVAFRFTR
GNEVLYSLCVIPVMSWTVCTVLERELYERNQLLACS-KTTTQMIMFYLSWLMKHRVSNAW
QNLQQ--FLHKLCSLAADGIWK-HKVLFEEKEIEDQGLNQPQLLSLFLNEKGLEKGTDHV
NVYSFSHLHLQELFAAMFYVLEDQDGMVSDSRILA--------KDVNMLLESYHTSRMDL
NVTVRLLFGLVNPKSVEYAGEGIGCRISLQPREDLLRWLQTRPRGTSHPRE---------
--------------VMKIEDLDTFHLLFETNEKSFVQSVLGSFTG---------------
-IALQDVKLTLYDQAALCFCIKQWAGLLSVTLRSCSFHQQHHR-----------------
---------------------QEPAKGLPRQSWRQEELHSPLHPLCQALGHP------GS
SLQSLR--
------------------------LQWCGLTEGDSGALGTLLATLPSLVHLELGDGALGD

DGVRMLCAGLRQPGCQLRVLR---------------------------------------

