# Supplementary material for: Evolution and functional divergence of NLRP genes in mammalian reproductive systems
Source: BMC Evol Biol. 2009 Aug 14;9:202. doi: 10.1186/1471-2148-9-202 (PMC2735741; doi:10.1186/1471-2148-9-202)
Supplement: Additional file 4 — The mouse V1r sampling used for phylogeny. The Table shows the mouse V1r sampling used for phylogenetic analysis (Figure 3). [file 1471-2148-9-202-S4.pdf]

Additional file 4: The mouse *V1R* sampling used for phylogeny

Gene Symbol	Protein symbol	Genomic Location	Protein Accession Number
V1rj2	V1RJ2	7A1 (region I)	NP_598987
V1rj3	V1RJ3	7A1 (region I)	NP_665846
V1rk1	V1RK1	7A1 (region I)	NP_598988
V1rg1	V1RG1	7A1 (region I)	NP_665842
V1rg2	V1RG2	7A1 (region I)	NP_598964
V1rg5	V1RG5	7A1 (region I)	NP_598967
V1re9	V1RE9	7A1 (region II)	NP_665841
V1re10	V1RE10	7A1 (region II)	NP_598990
V1re11	V1RE11	7A1 (region IV)	NP_598991
V1re12	V1RE12	7A3 (region III)	NP_598992
V1re13	V1RE13	7A1 (region I)	NP_665847
V1rd1	V1RD1	7A1 (region IV)	NP_109667
V1rd2	V1RD2	7A1 (region IV)	NP_109666
V1rd4	V1RD4	7A1 (region IV)	NP_109664
V1rd6	V1RD6	7A1 (region IV)	NP_109663
V1rd9	V1RD9	7A3 (region III)	NP_109660
V1rd10	V1RD10	7A1 (region IV)	NP_997426
V1rd13	V1RD13	7A3 (region III and V)	NP_996751
V1rd14	V1RD14	7A3 (region III and V)	NP_109661
V1rd15	V1RD15	7A3 (region III and V)	NP_987074
V1rd16	V1RD16	7A3 (region III and V)	NP_996752
V1rd18	V1RD18	7A3 (region III and V)	NP_997501
V1rd20	V1RD20	7A3 (region III and V)	NP_997429
V1rd21	V1RD21	7A3 (region III and V)	NP_997430
V1rd22	V1RD22	7A3 (region III)	NP_997431

The division of region I-V is described as in Figure 3.
